# Redefining vascular repair: revealing cellular responses on PEUU—gelatin electrospun vascular grafts for endothelialization and immune responses on *in vitro* models

**DOI:** 10.3389/fbioe.2024.1410863

**Published:** 2024-06-05

**Authors:** María A. Rodríguez-Soto, Alejandra Riveros-Cortés, Ian C. Orjuela-Garzón, Inés María Fernández-Calderón, Cristian F. Rodríguez, Natalia Suárez Vargas, Carlos Ostos, Carolina Muñoz Camargo, Juan C. Cruz, Seungil Kim, Antonio D’Amore, William R. Wagner, Juan C. Briceño

**Affiliations:** ^1^ Department of Biomedical Engineering, Universidad de los Andes, Bogotá, Colombia; ^2^ Instituto de Química, Facultad de Ciencias Exactas y Naturales, Universidad de Antioquia, Medellín, Colombia; ^3^ McGowan Institute for Regenerative Medicine and Department of Bioengineering, University of Pittsburgh, Pittsburgh, PA, United States; ^4^ Department of Congenital Heart Disease and Cardiovascular Surgery, Fundación CardioInfantil Instituto de Cardiología, Bogotá, Colombia

**Keywords:** tissue engineered vascular grafts, regenerative medicine, biomaterials, inflammatory response, immunomodulation, M1/M2 macrophage polarization, endothelialization, cell signaling

## Abstract

Tissue-engineered vascular grafts (TEVGs) poised for regenerative applications are central to effective vascular repair, with their efficacy being significantly influenced by scaffold architecture and the strategic distribution of bioactive molecules either embedded within the scaffold or elicited from responsive tissues. Despite substantial advancements over recent decades, a thorough understanding of the critical cellular dynamics for clinical success remains to be fully elucidated. Graft failure, often ascribed to thrombogenesis, intimal hyperplasia, or calcification, is predominantly linked to improperly modulated inflammatory reactions. The orchestrated behavior of repopulating cells is crucial for both initial endothelialization and the subsequent differentiation of vascular wall stem cells into functional phenotypes. This necessitates the TEVG to provide an optimal milieu wherein immune cells can promote early angiogenesis and cell recruitment, all while averting persistent inflammation. In this study, we present an innovative TEVG designed to enhance cellular responses by integrating a physicochemical gradient through a multilayered structure utilizing synthetic (poly (ester urethane urea), PEUU) and natural polymers (Gelatin B), thereby modulating inflammatory reactions. The luminal surface is functionalized with a four-arm polyethylene glycol (P4A) to mitigate thrombogenesis, while the incorporation of adhesive peptides (RGD/SV) fosters the adhesion and maturation of functional endothelial cells. The resultant multilayered TEVG, with a diameter of 3.0 cm and a length of 11 cm, exhibits differential porosity along its layers and mechanical properties commensurate with those of native porcine carotid arteries. Analyses indicate high biocompatibility and low thrombogenicity while enabling luminal endothelialization and functional phenotypic behavior, thus limiting inflammation in *in-vitro* models. The vascular wall demonstrated low immunogenicity with an initial acute inflammatory phase, transitioning towards a pro-regenerative M2 macrophage-predominant phase. These findings underscore the potential of the designed TEVG in inducing favorable immunomodulatory and pro-regenerative environments, thus holding promise for future clinical applications in vascular tissue engineering.

## 1 Introduction

Cardiovascular diseases stand as the foremost cause of global mortality, as documented by the World Health Organization (WHO) (De Hert et al., 2018; Kaptoge et al., 2019; Nowbar et al., 2019; Al-Makki et al., 2022). Concurrently, failures in vascular grafts significantly diminish the efficacy of surgical treatments for conditions like atherosclerosis, aneurysms, coronary artery disease, and carotid artery disease (de Vries et al., 2016; Carrabba and Madeddu, 2018; Rodriguez-Soto et al., 2021). Main causes for vascular graft failure have been identified as thrombogenesis, intimal hyperplasia, and calcification (Allon et al., 2011; de Valence et al., 2012; Cheung et al., 2017; Duque et al., 2017; Yang et al., 2019; Feng et al., 2022; Rodriguez-Soto et al., 2022; Zizhou et al., 2022; Jin et al., 2023). Current vascular grafts often fail due to their inability to replicate the intricate structure of natural blood vessels, which can lead to inflammatory reactions compromising the graft’s function and patency.

Arteries’ native extracellular matrix consists of three distinct layers with specific features and compositions, providing the necessary compliance to regulate blood pressure while ensuring consistent blood flow (van Haaften et al., 2018; Riveros et al., 2022). The intima layer prevents blood clotting, regulates muscle tone, and controls inflammation through endothelial cells. The media layer contains smooth muscle cells arranged in a circular pattern, responding to changes in blood pressure by contracting or dilating. The external layer, rich in collagen fibers, allows stretching and maintains compliance even at high pressures (Fahad et al., 2023; Snyder et al., 2023). Furthermore, during blood vessel formation, bioactive molecules and the structural properties of the extracellular matrix work together to guide tissue formation across the vessel’s layers (Serbo and Gerecht, 2013; Sankaran et al., 2015; Hussey et al., 2018; Li et al., 2019; Chiang et al., 2021; Rodriguez-Soto et al., 2021; Rodriguez-Soto et al., 2022).

Recently, there has been a shift toward designing tissue-engineered vascular grafts (TEVGs) that mimic native artery structures to stimulate blood vessel regeneration and improve treatment outcomes. Approaches such as multilayered structures, bioactive molecule incorporation for cell interactions, and antithrombogenic compound integration are being explored for scaffold effectiveness.

Multilayered tissue-engineered vascular grafts (TEVGs) using natural and synthetic polymers are effective in replicating native vessel functionalities. For instance, to achieve multilayered structures electrospinning technique has been used to create interconnected porous structures, allowing for the customization of mechanical and biological properties by adjusting polymer compositions and combinations (Huang and Niklason, 2014; Gong et al., 2016; Jirofti et al., 2018). Fine-tuning electrospinning parameters such as voltage, speed, time, and polymer concentration enable control over structural properties like fiber size and scaffold thickness (Huang and Niklason, 2014).

To this end, the use of polymer concentration gradients in the development of TEVGs can represent a combined strategy to guide the behavior of the cells repopulating the vascular walls (Park et al., 2019; Ryan et al., 2020; Fahad et al., 2023). For instance, multilayered tissue-engineered vascular grafts (TEVGs) using natural and synthetic polymers appear to be effective in replicating native vessel functionalities. TEVGs based on polycaprolactone–polyethylene glycol methyl ether, polycaprolactone–chitosan electrospun scaffolds, and PU/PET scaffolds have shown to be potential options for vascular regeneration (Gong et al., 2016; Radakovic et al., 2017; Sultana et al., 2017; Khodadoust et al., 2018; Amirian et al., 2020). Likewise, multilayer vascular grafts combining different materials and structures featuring, for instance, a thin, dense, nanofibrous poly-ε-caprolactone core and a thick, porous hydrogel sleeve of genipin-crosslinked collagen-chitosan have also been reported (Madhavan et al., 2013). As the use of natural polymer-derived grafts have demonstrated degradation of scaffolds and the potential for neo-artery formation, indicating prospects for tissue regeneration (Dong et al., 2018), as well as shown compatibility with cell culture and ingrowth, (Madhavan et al., 2013), the incorporation of gradients with natural polymers in the development of TEVGs might also be a strategy to guide the behavior of the cells repopulating the vascular walls (Park et al., 2019; Ryan et al., 2020; Fahad et al., 2023).

On the other hand, ensuring hemocompatibility stands as a crucial requirement to prevent thrombogenesis and mitigate immune responses. Different strategies have been evaluated, the most common approach is the coating with antithrombogenic molecules such as heparin and fibronectin. Other coatings include the use of anticoagulant peptides and nanoplatforms with antithrombogenic drugs (Chen et al., 2019; Xu et al., 2021). One of the most successful strategies is the incorporation of polyethylene glycol (PEG) derivatives. With stablished hydrophilic properties, non-toxicity, and resistance to protein fouling, PEG derivatives have been widely used (Seeto et al., 2013; Skardal et al., 2010; Wang et al., 2022; Yadav et al., 2021) and have been shown to improve long-term patency of TEVGs in a swine model (Valencia-Rivero et al., 2019).

Bioactivity for TEVG regeneration mainly targets endothelial lining. Strategies involve growth factors like VEGF (vascular endothelial growth factor) and FGF (Fibroblast Growth Factor), promoting endothelial cell growth and migration. Moreover, nitric oxide compounds enhance endothelial function and inhibit platelets, while peptides like RGD (Arginylglycylaspartic acid) and REVD bind to integrins on endothelial progenitor cells (Wang et al., 2015; Xie et al., 2022; Xue et al., 2019).

For instance, although REVD has shown to target specifically endothelial cells, RGD has demonstrated significant results in enhancing overall regeneration. In fact, different studies have investigated the use of RGD–PEG materials for the manufacturing of vascular grafts. WS Choi et al explored the modification of polyurethaneurea with PEG and different adhesive peptides to improve endothelialization while preventing platelet adhesion, showcasing the relevance of RGD-PEG materials for vascular grafts (Choi et al., 2016). Iglesias-Echevarria et al developed a novel platform for creating vascular grafts with mimetic arterial mechanics and physiologically relevant 3D arterial environments using RGD-PEG materials. This study used coaxial electrospinning to fabricate nanostructured hybrid fibers that mimic both the structural and mechanical properties of the vascular extracellular matrix. The study highlighted the ability of RGD-PEG materials to stimulate cell activities and promote the differentiation of mesenchymal stem cells into vascular smooth muscle cells, suggesting their potential for arterial regeneration and functional activity on the graft wall (Iglesias-Echevarria et al., 2021). Moreover, Noel et al discussed the response of PEG samples coated with adhesive peptides such as RGD. Key findings include the significant reduction of cell and platelet adhesion on PEG-coated samples and the high adhesive properties of RGD peptide. Emphasizing the promising nature of the RGD peptide for enhancing endothelialization and reducing thrombogenicity in vascular graft applications (Noel et al., 2015).

Finally, bioactivity in TEVGs also necessitates managing immune responses and promoting graft acceptance. This has led to proposals for using bioactive molecules like IL-10 and TGF-β, known for their immunomodulatory effects that reduce inflammation. Additionally, biomaterials with immunomodulatory properties, such as decellularized matrices and biodegradable polymers, have been suggested.

The trajectory towards developing long-patency vascular grafts depends not only on elucidating the molecular mechanisms underlying endothelialization, inflammation, and immunomodulation but also on understanding and achieving the optimal mechanical and physicochemical properties that accurately mimic native vascular vessels, alongside the exploration of potential therapeutic avenues for modulating endothelial cell activation and inflammation (Amersfoort et al., 2022; Chen et al., 2020; Rodriguez-Soto et al., 2022; Wang and Zhang, 2020).

Herein, we aimed to develop an innovative tissue-engineered vascular graft devised to amplify cellular responses by integrating a physicochemical gradient through a multilayered structure using synthetic [poly (ester urethane urea)] PEUU and natural polymers (Gelatin B), creating a polymer gradient aimed to reduce inflammatory responses Additionally, we mitigated potential microenvironmental mismatches by iteratively selecting the most adequate manufacturing conditions to achieve optimal pore size, fiber diameter, and mechanical properties for multilayered vascular grafts. In addition, the luminal surface is functionalized with a four-arm polyethylene glycol (P4A) to inhibit thrombogenesis while fostering the adhesion and maturation of functional endothelial cells with adhesive peptides (RGD/SV).

## 2 Materials and methods

### 2.1 Materials

For polymer synthesis and blends check Supplementary Material. Biological assays used reagents such as Triton X-100, Phosphate Buffer Saline (PBS), thiazolyl blue tetrazolium bromide (MTT), dimethyl sulfoxide (DMSO, 99%), Epinephrine (1236970), and Tween 20 (P1379) were purchased from Sigma-Aldrich in St. Louis, MO, United States. For cell line details and expansion reagents check Supplementary Material. For biomolecular assays phorbol 12-myristate 13-acetate (PMA-P8139), Lactate Dehydrogenase (LDH) kit (MAK066), fluorometric Intracellular ROS kit (MAK145) and a Bicinchoninic Acid (BCA) Assay Kit (Quanti-Pro M3685) was acquired from Sigma Aldrich from St. Louis, MO, United States. Nitric Oxide Assay kit was acquired from Abnova (KA1641) from Taipei, Taiwan, and Human VEGF Quantikine ELISA Kit (DVE00) from RnDSystem Minneapolis, MN, United States. For immunofluorescence assays check Supplementary Material. LEGENDplex™ Human Macrophage/Microglia Panel (10-plex) with V-bottom Plate was purchased from BioLegend in San Diego, CA, United States. For analysis of gene expression TRIzol™ Reagent (15596026) was purchased from Sigma Aldrich from St. Louis, MO, United States, 2-propanol (131090), Ethanol absolute (131086), Chloroform stabilized with ethanol (131252), Sodium citrate (814029RC), Sodium Hydroxide (303126), Hydrochloric Acid (303112), Sodium Hypochlorite (211921), L-Glutamic Acid (A1704), and HEPES (A3268) were purchased from PanReac Applichem GmbH (Darmstadt, Germany). Luna^®^ Universal One-Step RT-qPCR Kit (NEB #E3005S) was purchased from New England Bioabs, Inc. Ipswich, MA, United States.

### 2.2 Methods

#### 2.2.1 Multilayered vascular graft fabrication

##### 2.2.1.1 Luminal layer fabrication

The luminal layer was fabricated by *in situ* functionalization PEUU-COOH, incorporating PEG 4 Arm NH_2_ along with the peptides RGD and SV via the EDC/NHS chemistry to form amide bonds in an HFIP solution as previously described (Rodríguez-Soto et al., 2023) as outlined on Supplementary Methods 3.1. This functionalized polymer solution in HFIP was subsequently used to create the luminal layer via electrospinning. The flow rate was adjusted at 1.3 mL/h for 30 min, the needle-to-tip distance was carefully maintained at 11 cm, and a voltage of 9 kV was applied to a stainless-steel mandrel with a 3.75 mm diameter, rotating at a speed of 1,000 rpm, ensuring the formation of densely packed fibers.

##### 2.2.1.2 External layers fabrication

To fabricate the external layers, a gradient of PEUU and gelatin was achieved using different PEUU: Gelatin blends (75:25, 85:15, and 95:05 w/w). PEUU pellets and gelatin powder were dissolved in HFIP to a final concentration of 12.5% (w/v) under magnetic stirring at 30°C. The electrospinning procedure was then carried out, with a controlled flow rate set at 2 mL/h for each layer, spanning 20 min for 75:25 and 85:15 layers, and 30 min for 95:5. The needle-to-tip distance was consistently maintained at 11 cm relative to the mandrel, which had the luminal layer deposited and rotated at a speed of 200 rpm. Voltage adjustments were made for each layer as follows: 6 kV for the first layer (75:25), 7 kV for the second layer (85:15), and 8 kV for the third layer (95:05).

The structure was removed dry from the mandrel creating a corrugated structure for improved kinking resistance, and then placed in a sealed glass container with 12.5% (v/v) glutaraldehyde solution, gently agitated at 10 rpm at room temperature for 5 h. After that, it was quenched using a 5% w/v glutamic acid solution (dissolved in 1.8% v/v hydrochloric acid), agitated gently for 1 h, rinsed with distilled water for 30 s, and repeated the quenching process three times.

The resulting structure, referred to as Multilayered + PEG 4 Arm NH2 + PEPTIDES (ML + P + P) TEVG, consists of a luminal layer modified for function, followed by three outer layers of PEUU with varying gelatin content, creating a gradient. A Multilayered construct functionalized only with PEG 4 Arm NH2 (ML + P) was used as a control, as well as monolayer controls that were obtained using pristine PEUU (MO) or various PEUU: Gelatin blends (75:25, 85:15, or 95:5) for mechanical testing. Supplementary Figure S1 illustrates the TEVG structure and manufacturing method.

#### 2.2.2 Morphology

The morphology of the fabricated ML + P + P TEVG was examined using Scanning electron microscopy (SEM) analysis conducted with a JSM-6490LV^®^ microscope (JEOL USA Inc., Peabody, MA, United States) equipped with a 10 kV accelerating voltage. Samples were securely affixed to aluminum plates using carbon tape followed by a thin gold layer application using the Vacuum Desk IV apparatus from Denton Vacuum (Moorestown, NJ, United States). Post-processed SEM micrographs were analyzed utilizing the Fiji^®^ and ImageJ^®^ software packages (version 5.2.0, National Institutes of Health, Bethesda, MD, United States) to determine fiber thickness and pore diameter. Additionally, segments of the ML + P + P TEVG were taken to the histology service of Fundación Santa Fé (Bogotá, Colombia), to perform Masson’s trichrome staining to verify the gelatin inclusion. For ML + P + P TEVG stability in aqueous media, water uptake, and porosity measurements were performed by liquid displacement. More extensive details can be found in Supplementary Methods 3.2.

#### 2.2.3 Mechanical properties

The mechanical properties of the ML + P + P TEVG were evaluated and compared to the Monolayer constructs (MO) of PEUU alone, and 75:25, 85:15, or 95:5 formulations alongside Porcine Carotid Artery (PA) as a control to assess equivalence. Longitudinal tensile strength, circumferential tensile strength, and suture retention tests were performed according to ISO 7198:2016(E). An INSTRON 5585 (Norwood, MA, United States) Uniaxial Tensile Testing machine equipped with a 5 kN load cell was used for all tests. All samples were pre-humidified with a PBS solution at 37°C, following the guidelines outlined in ASTM F3225-17. Afterward, the samples were securely clamped by the machine’s jaws and lined with plastic wrap to ensure a secure grip. Length measurements were taken with a digital caliper (Mitutoyo 500-196-30 Digital Caliper, Kawasaki, Japan) with a resolution of 0.01 and a 0–150 mm measurement range.

In both longitudinal and circumferential tensile strength tests, variables such as the load at yield, break, or maximum load (T_max_), and rate of extension were measured. A constant extension rate of 50 mm/min was maintained, and data concerning extension and load curves were recorded and processed into stress-strain curves. The Ultimate Tensile Stress was determined by analyzing the longitudinal stress-strain curve and the Maximum Load was determined by analyzing the longitudinal load-extension curve. For more in-depth information, refer to in Supplementary Methods 3.3.

#### 2.2.4 Physicochemical characterization

##### 2.2.4.1 Chemical surface analysis of the luminal layer

The chemical surface characterization of the ML + P + P TEVG was conducted by X-ray photoelectron spectroscopy (XPS) technique from the functionalized luminal layer. The PEG 4 Arm NH_2_ functionalization (ML + P) followed by the anchoring of the bioactive RGD and SV peptides (ML + P + P) were evaluated. The data were recorded from a photoelectron spectrometer (SPECS Surface Nano Analysis GmbH, Germany) equipped with a PHOIBOS-150 hemispherical electron energy analyzer and a µfocus-600 Al X-ray source under ultra-high vacuum conditions. Specific protocol is available in Supplementary Methods 3.4.1.

Moreover, the physicochemical evaluation of ML + P + P TEVG was conducted by Fourier Transform Infrared (FTIR) analysis, where the infrared spectra were evaluated on an Alpha II FTIR Eco-ART (Bruker Optik GmbH, Ettlingen, Germany) from 4,000 to 600 cm^−1^ with a spectral resolution of 2 cm^−1^. Data was expressed as transmittance, and the peaks of PEUU COOH and ML + P + P transmittances were analyzed and compared with the reference pristine components. The PEUU data and PEUU Gelatin B 85:25 data were analyzed and compared to identify the incorporation of the Gelatin B. Further details are provided in Supplementary Methods 3.4.1.

To further elucidate the incorporation of the molecules through the formation of covalent bonds of the PEUU backbone with the bioactive molecules (i.e., PEG 4 arm NH_2_ and Peptides) or to identify the inclusion of gelatin in the blends, thermal properties were evaluated by Thermogravimetric Analysis (TGA). ML + P + P TEVG or monolayer (MO) samples were subjected to a linear temperature ramp up to 600°C at a rate of 10°C/min (ASTM E1131) in a TA Instruments Q600 thermogravimetric analyzer^®^ (New Castle, DE, United States) under nitrogen atmosphere (100 mL/min). Additionally, a DSC analysis was conducted following the ASTM D3418 standard using a TA Instruments Q2000 instrument (New Castle, DE, United States). The analysis was performed under a nitrogen atmosphere with a continuous flow rate of 300 mL/min. The temperature program included a heating cycle from 0°C to 600°C at a rate of 10°C/min.

##### 2.2.4.2 Modeling of the ML + P + P TEVG degradation

The ML + P + P TEVG degradation was studied with a multiphysics model approach in the COMSOL Multiphysics 6.1^®^ software (COMSOL Inc., Stockholm, Sweden). This model used a mathematical degradation dynamics model coupled with a dilute species transport in a porous media model (Bolanos-Barbosa et al., 2023). The analysis encompasses the degradation processes affecting the graft, which occur through three distinct mechanisms: 1) degradation induced by reactive oxygen species (ROS); 2) enzymatic degradation originating from the infiltration of macrophages into the ML + P + P TEVG; and 3) hydrolysis prompted by the aqueous environment in which the graft is situated. Within the model, five distinct chemical species were considered, namely: graft, macrophages, water, lipases, and ROS. Details o on governing equations are available in Supplementary Methods 3.4.2.

**FIGURE 1:**
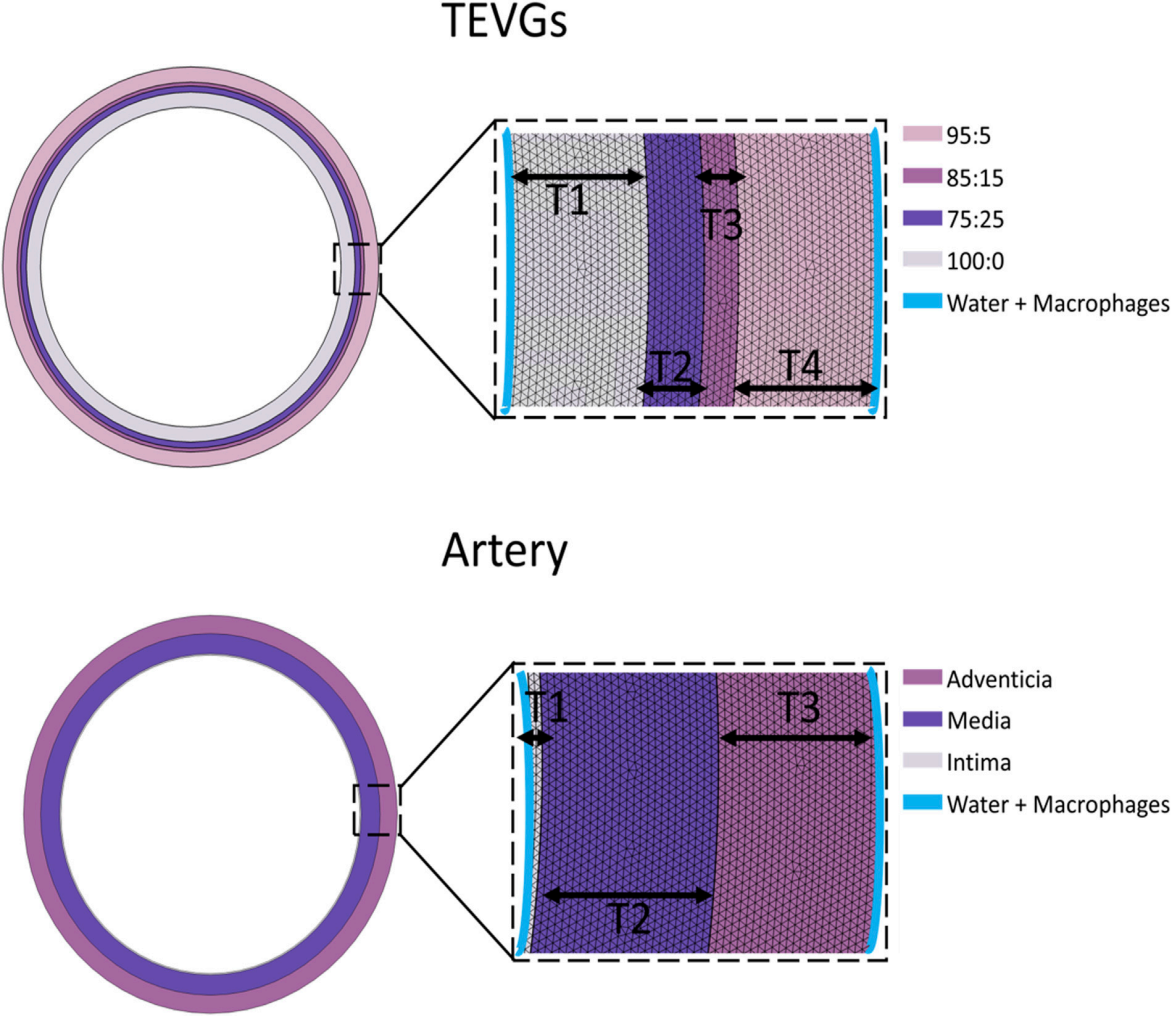
Mesh and boundary conditions representation of the computational domain.

A time-dependent approach, in conjunction with a MUltifrontal Massively Parallel sparse direct Solver (MUMPS), was employed to solve the subset of equations derived from the multiphysics model (Rodríguez et al., 2023). Detailed boundary conditions for the simulation, delineating the regions where macrophages were initialized and the location of the water, can be found in Figure 1. The computational domain for our model was created utilizing a meshing technique that incorporated 115,588 domain elements and 5,672 boundary elements for the TEVGs and 107,340 domain elements and 5,598 boundary elements. These specific meshing levels were chosen to ensure comprehensive coverage. The thickness of the layers was determined by experimentally identified averages, as detailed in Supplementary Table S1. Other simulation input parameters are also outlined therein, while the remaining parameters were fine-tuned to align the mathematical model with experimental outcomes.

The model validation involved the utilization of an *in-vitro* system to assess enzymatic and reactive oxygen species (ROS)-mediated degradation. Specifically, this evaluation entailed the application of lipase at a concentration of 20,000 units and hydrogen peroxide (H_2_O_2_). The selection of these parameters was guided by the anticipated macrophage infiltration density within the tissue-engineered vascular graft (TEVG), as supported by relevant literature (Wissing et al., 2019). The grafts were subjected to a bi-weekly regimen, involving washing, weighing, and replenishing of the respective solutions. Each washing cycle encompassed a triple rinse with water, and the samples were resuspended 15 times in water to ensure comprehensive coverage and thorough assessment of TEVG and artery degradation.

#### 2.2.5 Biocompatibility

The cytocompatibility and hemolytic behavior of the ML + P + P TEVG were determined according to the ISO 10993 standard and were compared to the monolayer construct (MO) and the Multilayered structure functionalized with only PEG 4 arm NH_2_ (ML + P). Sample preparation and sterilization are specified in Supplementary Methods 3.5.1.

The hemolysis test was performed as previously described (Rodríguez-Soto et al., 2023), informed consent was obtained to collect O+ human blood on EDTA tubes and erythrocytes were isolated and resuspended in PBS to create a stock solution of 4 × 10^6^ erythrocytes/μL. Sterilized films were submerged in the erythrocyte solution, with negative and positive controls consisting of PBS and 1% Triton X-100, respectively. Incubation was performed at 37°C for 1 h, followed by centrifugation at 324 g for 5 min; the absorbance of the supernatant was measured at 454 nm. The positive control, Triton X-100, was used to determine the hemolysis percentage. More specifics can be found in Supplementary Methods 3.5.2.

For cytocompatibility evaluation, a metabolic activity assay was performed using MTT on HUVECS cells, L929 cells, and THP-1 cells; cell expansion is described in Supplementary Methods 3.5.3. For this purpose, 5.0 × 10^4^ L929 cells were seeded onto 96-well plates containing DMEM supplemented with 10% FBS and allowed to adhere for 24 h at 37°C in a 5% CO_2_ atmosphere. 1.0 × 10^4^ HUVECS cells were seeded onto 96-well plates containing complete EGM-2 and allowed to adhere for 24 h at 37°C in a 5% CO_2_ atmosphere. For validation on suspension cells, 5 × 10^4^ THP-1 cells were seeded onto 96-well plates containing RMPI supplemented with 10% FBS. All Cells were then exposed to previously sterilized 0.4 cm^2^ samples for an additional 24 h and 72 h in serum-free media. DMSO at a concentration of 10% v/v and untreated cells were used as negative and positive controls, respectively. Post incubation, films were carefully removed, and the MTT solution was applied to allow formazan crystal formation over a 2-hours incubation period. Media was removed after centrifuging the culture plates at 250 g for 5 min, and DMSO was added to dissolve the formazan crystals. Absorbance was then read at 595 nm, with cell viability percentages benchmarked against a live cell control.

#### 2.2.6 Antithrombotic properties

As the luminal surface of the ML + P + P TEVG was functionalized with PEG 4 Arm NH_2_ to enhance its anti-thrombotic properties and promote long-term patency, we analyzed such properties on ML + P + P TEVG, MO, and ML + P constructs. To this end, a series of assays were conducted to investigate protein adsorption, platelet aggregation, activation, and clot formation according to previously described procedures (Rodríguez-Soto et al., 2023).

PEG 4 Arm NH2’s high hydrophilicity could reduce platelet aggregation by altering protein adsorption. Surface wettability was evaluated by measuring contact angles using a 100 μL droplet of Type II water on a 1 cm^2^ sterilized sample.

For the assessment of protein adsorption capacity, 0.5 cm^2^ sterile samples were immersed in a 10% Fetal Bovine Serum (FBS) solution and incubated for 12 h. Proteins adhered to the surface of the samples were detached using Dodecyl Sulfate (SDS) and the protein concentration in the supernatant was quantified using a Bicinchoninic Acid (BCA) Assay Kit (Quanti-Pro M3685, Sigma Aldrich, St. Louis, MO, United States). More information is provided on Supplementary Methods 3.5.4.

To assess platelet aggregation and activation, fresh O+ human blood was collected in sodium citrate tubes and platelet-rich plasma (PRP) was obtained by centrifuging the anticoagulated blood e at 180 g for 10 min 0.5 cm^2^ sterile samples were exposed to 200 μL of PRP activated with 0.1 M CaCl₂ for 20 min. The supernatant absorbance was measured at 620 nm, with Films functionalized with epinephrine serving as positive controls for platelet aggregation. The aggregation was expressed as a percentage relative to the epinephrine control.

Scanning electron microscopy (SEM) analysis was performed with an SEM model JSM-6490LV^®^ (JEOL USA Inc., Peabody, MA, United States) working at a 10 kV accelerating voltage to examine platelet presence and activation. 0.5 cm^2^ sterilized samples were exposed to activated PRP for 30 min with gentle agitation at 10 rpm, followed by fixation in 4% v/v glutaraldehyde for 30 min. The films were washed three times with PBS 1X, subjected to a decreasing ethanol gradient for drying, mounted on aluminum plates with carbon tape, and gold-coated using a Vacuum Desk IV apparatus (Denton Vacuum, Moorestown, NJ, United States).

A lactate dehydrogenase assay (LDH) quantified platelet adhesion to film surfaces exposed to activated PRP for 1 h. The films were transferred to new plates, and platelets were lysed using 1% Triton X-100 for 5 min. Following film removal, LDH working solution (MAK066, Sigma Aldrich, St. Louis, MO, United States) was added, and absorbance at 493 nm was recorded. Platelet counts were determined using a linear regression model based on a known platelet count of 4.1 × 10^5^ platelets/µL, and the platelet count was then normalized relative to the surface area (Braune et al., 2015).

To assess whole blood clotting on film surfaces, O+ whole blood from human donors was collected in sodium citrate tubes, discarding the first tube to prevent contamination. The blood was mixed with 0.1 M CaCl₂ for coagulation restoration. 200 μL of activated blood was then applied to 1.5 cm^2^ sterilized samples in 12-well plates and incubated for 1 h at 37°C to promote clot formation. After incubation, 3 mL of Type II water was added to each sample and incubated for 5 min. The supernatant was transferred to a new plate, and absorbance was measured at 540 nm. A glass slide served as a positive control for coagulation, with absorbance indicating free hemoglobin released by red blood cells not protected by polymerized fibrin mesh, inversely reflecting thrombus formation (Sabino and Popat, 2020).

#### 2.2.7 *In vitro* cell culture

To reveal the cellular responses on the ML + P + P TEVG, endothelialization and immune responses were evaluated on *in-vitro* models through immunofluorescence, RNA expression analysis, nitric oxide (NO), cytokine release, and intracellular reactive oxygen species production (ROS).

For all samples with seeded cells sterile samples were used prior any test, protein adsorption was allowed 48 h as specified in Supplementary Methods 3.6.1. For immunofluorescence stains sample preparation required an autofluorescence quenching protocol including a specific blocking protocol detailed in Supplementary Methods 3.6.2 and 3.6.3.

To assess endothelialization, ML + P + P TEVG and MO group samples were placed on culture plates, exposing their luminal surfaces. HUVECs (5 × 10^4^ cells) suspended in EGM-2 media were gently seeded using a drop method, with a 30 μL drop per sample to maintain surface tension. After a 45-minutesadhesion period, additional EGM-2 media fully submerged the samples. Cell media was refreshed every 2 days, and cultures were maintained for 7 days.

The same seeding procedure was replicated for analyzing endothelial function and its immunomodulatory role. On day 3, the cell media was removed, and 1 × 10^5^ THP-1 cells were seeded in hydrocortisone-free EGM-2 media supplemented with 50 ng/mL PMA to induce macrophage differentiation. The PMA-enriched medium was replaced with hydrocortisone-free EGM-2 media 24 h later, and the cultures were sustained for an additional 4 days, totaling 7 days with media changes occurring every 2 days (Barthes et al., 2018).

After these steps, cell media was extracted from both groups. Some samples were used immediately for NO release assays, while others were stored at −80°C for cytokine release analysis. For SEM analysis, samples were fixed with 2.5% v/v glutaraldehyde and dried with ethanol. For immunofluorescence, samples were fixed with 4% v/v formaldehyde and stored at 4°C. For RNA and DNA extraction, samples were disrupted using a cutting blade and preserved in Trizol at −80°C.

To determine the cell infiltration capacity on the vascular wall, 5 × 10^4^ L929 cells were seeded on the ML + P + P TEVG using the drop method with DMEM media supplemented with 10% FBS. The cell media was changed every 2 days, and cultures were maintained for 5 days. The cell media was then removed and the TEVG was fixed with either 2.5% v/v glutaraldehyde and ethanol dried for SEM analysis or 4 %v/v Formaldehyde for Immunofluorescence.

For immunomodulation analysis provided by the vascular wall, samples of ML + P + P TEVG, MO, and decellularized porcine artery (Supplementary Methods 3.6.4) were placed with their adventitial surfaces exposed. Afterward, 1 × 10^5^ THP-1 cells resuspended on RPMI media supplemented with 50 ng/mL PMA were seeded using the drop method. Following 45 min, well plates were centrifuged at 250 g for 5 min, and PMA-enriched cell media was replenished after 24 h to induce macrophage differentiation. After the initial 48 h, cell media was replaced with RPMI, and cultures were continued for five more days, totaling 7 days with media changes every 2 days. Upon culture completion, the cell media was removed. Some samples were used immediately for NO release assays, while others were stored at −80°C for cytokine release analysis.

For SEM analysis, samples were fixed with a 2.5% v/v glutaraldehyde solution and ethanol dried. Some samples underwent decellularization with 4.6% Sodium hypochlorite for 15 min under gentle agitation at 10 rpm at room temperature, washed twice with Type II water and ethanol drying for SEM analysis of fiber degradation (Wissing et al., 2019). Samples designated for Immunofluorescence were fixed using a 4% v/v formaldehyde solution and stored at 4°C. Samples intended for RNA and DNA extraction were disrupted with a cutting blade and preserved in Trizol stored at −80°C.

#### 2.2.8 Immunofluorescence staining

Cell morphology on HUVECs and Coculture samples were assessed using phalloidin/Hoechst stains, while M1/M2 polarization distribution on coculture and THP-1 experiment samples were identified using immunofluorescence with CCR7 (M1) and CD163 (M2) antibody markers (Kwiecień et al., 2019).

For all groups, the previously fixed samples were washed twice with PBS. Triton X-100 0.1% v/v was added for cell permeabilization and incubated for 5 min at room temperature, followed by two washes with PBS. Blocking proceeded with the addition of BSA 1% v/v, incubated for 1 h at room temperature, without a subsequent wash. For Phalloidin/Hoechst immunofluorescence, a solution of phalloidin conjugate (AF 488) 1:1,000 and Hoechst 1:2,000 was added to each sample and incubated for 60 min at room temperature. Samples were carefully washed twice, and fluorescence images at 20X magnification were collected on an Axio Vert.A1 microscope (Carl Zeiss, Oberkochen, Germany).

For M1/M2 identification, primary antibodies diluted in PBS with 0.05% v/v Tween 20% and 1% v/v goat serum were prepared and added to each sample using 1:1,000 rabbit monoclonal to CCR7 and 1:1,000 Mouse monoclonal to CD163. Following an overnight incubation at 4°C, the sample was washed twice with PBS. Secondary antibodies diluted in PBS with 0.05% v/v Tween 20% and 1% goat serum were prepared and added to each sample using 1:1,000 Goat Anti-Mouse IgG H&L (AF647) and Goat Anti-Rabbit IgG H&L (AF488) along with 1:2,000 Hoechst. Incubation proceeded for 2 h at 37°C and washed twice with PBS. Fluorescence images at 20X magnification were collected on an Axio Vert.A1 microscope (Carl Zeiss, Oberkochen, Germany). Reactive Oxygen Species (ROS), known as second messengers modulating specific cellular responses during inflammation and resolution were quantified intracellularly using a fluorometric Intracellular ROS kit (MAK145, Sigma Aldrich, St. Louis, MO, United States) following the manufacturer’s instructions. Additional information can be found in Supplementary Methods 3.7.1.

#### 2.2.9 DNA and RNA extraction

DNA and RNA extraction and purification were carried out using the guanidinium thiocyanate method (Trizol) following the manufacturer’s instructions (15596026 Sigma Aldrich, St. Louis, MO, United States). After phase separation of RNA/DNA (Supplementary Methods 3.7.2) DNA extraction was performed for cell number estimation through DNA quantification, details are reported in Supplementary Methods 3.7.3.

RNA extraction for gene expression analysis involved adding isopropanol to the aqueous phase from Trizol, incubating for 1 h, and centrifuging at 12,000 g for 10 min at 4°C to precipitate RNA and discard the supernatant. Ethanol 75% was used for RNA washing, with vigorous mixing on a vortex for 15 s, followed by centrifugation at 7,500 g for 5 min at 4°C. This washing step was repeated, and the supernatant was removed, allowing RNA to dry for 15 min. RNA was then resuspended in 30 µL of sterile water, and its concentration was determined by absorbance ratio at 260/280 nm using a DS-11 spectrophotometer (DeNovix, Wilmington, DE, United States). RNA integrity was verified through electrophoresis on a 0.8% agarose gel in TAE using 5 µL of sample (Supplementary Methods 3.7.4).

#### 2.2.10 Nitric oxide (NO) and cytokine release assays

For gene expression profiling, RT-qPCR was conducted using a One-Step RT-qPCR kit (NEB #E3005S, New England Biolabs, Inc., Ipswich, MA, United States) following the manufacturer’s protocol. A reaction mix containing enzyme mix, nuclease-free water, and 0.5 µg of template RNA for each sample was prepared. Primers for each gene (Supplementary Table S2) were added to microcentrifuge tubes, and the mixture was centrifuged to eliminate air bubbles. RT-qPCR was performed on a Rotor-Gene Q cycler (9001570, Qiagen N.V., Venlo, Netherlands) with an initial reverse transcription at 55°C for 10 min, followed by denaturation at 95°C for 1 min. Subsequent cycles included denaturation at 95°C for 10 s and extension at 60°C for approximately 30 s, totaling 40 denaturation cycles and 50 extension cycles. Nuclease-free water was used as a negative control. Copies exceeding the noise threshold were quantified, normalized to the reference gene (GAPDH), and relative expression levels were calculated using the 2^−ΔCt^ formula, reported as Log 2-Fold Change.

NO and cytokine levels were quantified in pooled supernatants at day 7 for HUVECs and Coculture groups and at day 3 and day 7 for THP-1 groups. NO was used as a determinant of the endothelial activity and inflammatory activity, we followed a standardized protocol as outlined by the manufacturer (KA1641, Taipei, Taiwan). The NO concentration was subsequently normalized relative to the DNA content and expressed as µM NO/µg DNA. Further elaboration is provided in Supplementary Methods 3.7.5.

VEGF, indicative of endothelialization and microvascularization promotion, was quantified using a VEGF immunoassay following the manufacturer’s guidelines (DVE00, RnDSystems, Minneapolis, United States). The VEGF concentration was subsequently normalized relative to the DNA content and expressed as µg VEGF/µg DNA. More details can be found Supplementary Methods 3.7.5.

Furthermore, a Human M1/M2 Macrophage Panel (10-plex) (LEGENDplex, BioLegend, San Diego, CA, United States) was used to quantify ten inflammatory-related factors involved in monocyte differentiation and macrophage functions (outlined on Supplementary Table S3). The protocol was performed according to the manufacturer’s instructions. The obtained results were expressed in pg cytokine/mL media, with data adjusted to be presented as the Log 2-Fold change. Further explanation can be found in Supplementary Methods 3.7.5.

#### 2.2.11 Data analysis and statistical analysis

Data analysis and statistical evaluations were performed using GraphPad Prism^®^ 9.1.1 software (Windows, GraphPad Software, San Diego, CA, United States, www.graphpad.com, accessed on 10 September 2023). Upon verifying data normality, independence of observations, and homoscedasticity, a two-way ANOVA test with Tukey’s multiple comparisons of means was employed. Data conforming to a normal distribution are presented as mean ± standard deviation, with statistical significance considered for *p*-values less than 0.05 (*p* < 0.05). Grubbs’ test was also used to identify potential single outliers within the data sets (data not shown).

For a better understanding of the methods here described, Supplementary Figure S2 is a schematic representation of the workflow for ML + P + P TEVG assessment.

## 3 Results and discussion

### 3.1 Macrostructure of the ML + P + P TEVG

This investigation led to the successful fabrication of a small-diameter multilayered tissue-engineered vascular graft (ML + P + P TEVG) using a blend of synthetic and natural polymers through electrospinning.

The TEVG featured a luminal surface functionalized with an anticoagulant molecule (PEG 4 arm NH_2_) and bioactive peptides to facilitate endothelial cell adhesion (RGD) and maturation (SV). On the other hand, the vascular wall was engineered with a physicochemical gradient through gelatin incorporation, aiming to induce tissue remodeling. The inclusion of gelatin is intended to offer sites for cell interaction and ameliorate inflammatory responses by mitigating the hyperactivation of pro-inflammatory cells.

**FIGURE 2:**
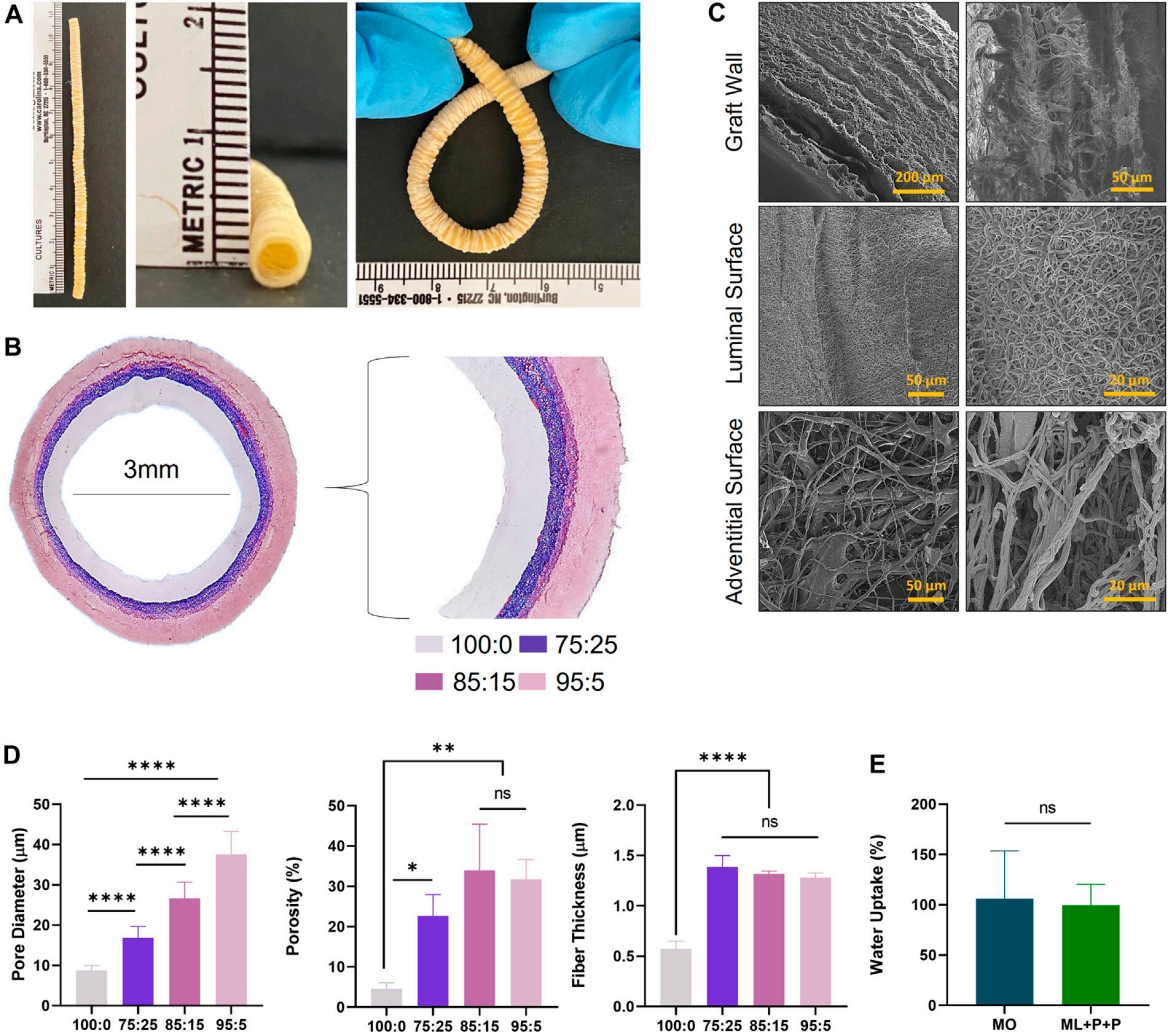
Macrostructure of the ML + P + P TEVG. **(A)** Length, diameter, and kink diameter. **(B)** SEM images of the graft wall, luminal surface and adventitial surface showing the fiber distribution. **(C)** Masson trichrome stain of the transversal section of the ML + P + P TEVG showing its four layers. **(D)** Fiber thickness, pore diameter and average porosity percentage of each TEVG layer. **(E)** Water uptake percentage of ML + P + P compared to monolayer sample (MO). (Mean ± SD) where, ns = no significant *p ≤ 0.05, **p ≤ 0.01, ***p ≤ 0.001, ****p ≤ 0.0001.

The ML + P + P TEVG structure is depicted in Figure 2A, showcasing an 11 cm graft with a 3.0 mm diameter and an 18.7 cm kinking diameter. Figure 2B illustrates the vascular composition using Masson trichrome stain, revealing four distinct layers. The luminal layer, functionalized with various bioactive molecules, appears clear. The purplish hue indicates the gelatin gradient introduced into the PEUU fibers at varying ratios (75:25, 85:15, and 95:5). As seen in Figures 2C, D, the electrospinning configuration used for the ML + P + P TEVG resulted in a luminal functionalized layer with thinner, densely packed fibers averaging 0.6 ± 0.1 µm in thickness, and a mean pore diameter of 8.8 ± 1.2 µm with an overall porosity of 4.6% ± 1.4. Conversely, the gelatin-containing layers consist of thicker fibers measuring 1.3 ± 0.1 µm, with a more porous architecture. Notably, the outer layers show increasing porosity and pore diameter. The 75:25 layer has an average porosity of 22.7% ± 5.3 and a pore diameter of 16.8 ± 2.8 µm, followed by the 85:15 layer with a porosity of 34.0% ± 11.4 and a pore diameter of 26.7 ± 4.0 µm, and the 95:5 layer exhibiting a porosity of 31.7% ± 4.9 and a pore diameter of 37.6 ± 5.7 µm.

The TEVG’s porosity design was carefully planned to prevent blood leakage while providing a sturdy surface for endothelial cell attachment and spreading. However, increased porosity and larger pore diameter on the adventitial surface are expected to enhance vascularization and tissue remodeling. This is crucial as cells from peripheral tissues surrounding the artery are a primary source for vascular wall remodeling (Matsuzaki et al., 2019). The porous structure aims to improve fluid and ion transfer through the graft (Lee et al., 2000). The reported pore diameters are higher than those in previous studies, where a 10 μm average pore size encouraged capillary ingrowth and graft regeneration, indicating good vascularization rates (Ozdemir et al., 2022). Water uptake tests, seen on Figure 2E, confirm that the ML + P + P TEVG maintains dimensional stability without morphological alterations.

### 3.2 Mechanical characterization of the ML + P + P TEVG

**FIGURE 3:**
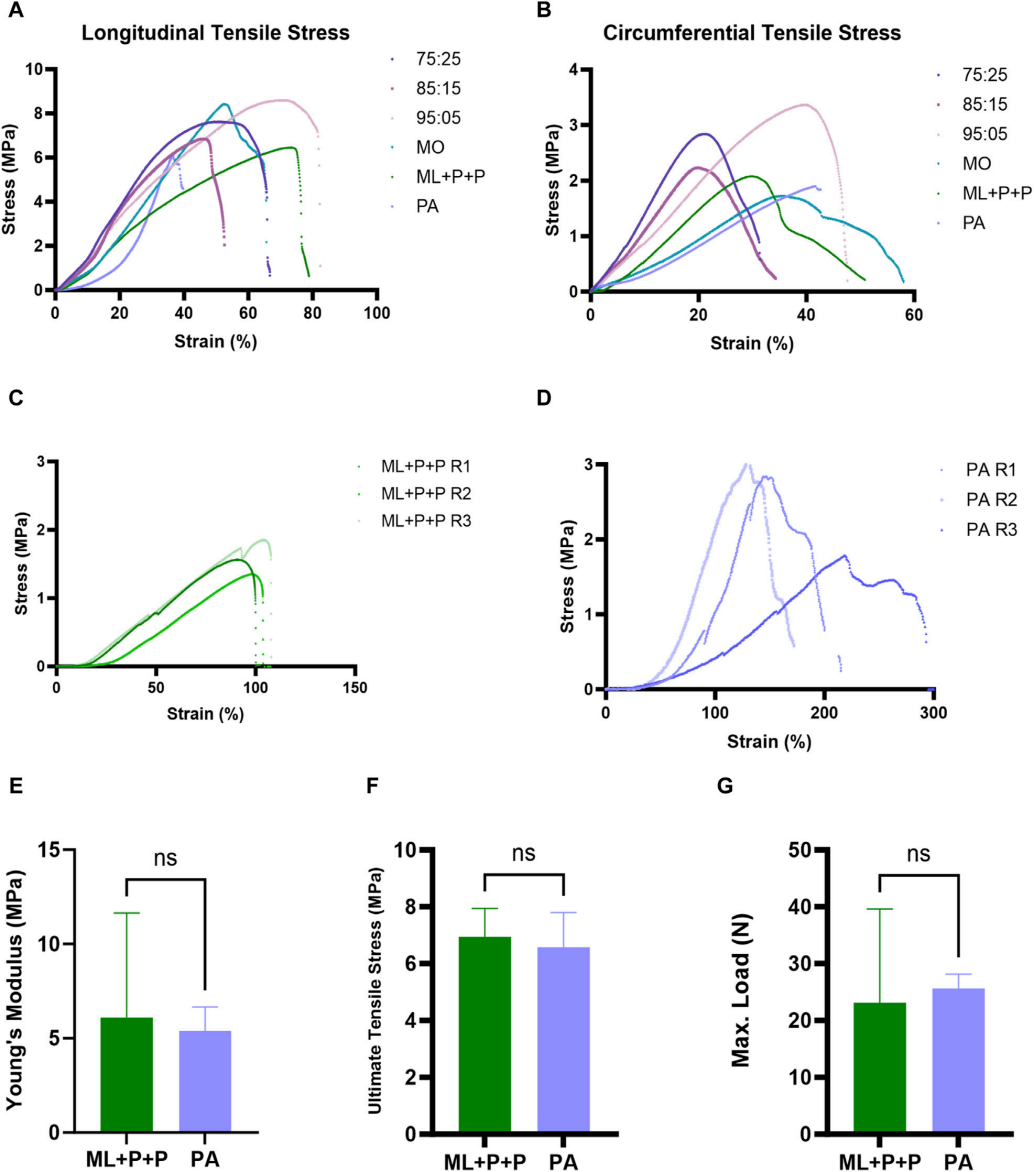
Mechanical properties. **(A)** Longitudinal tensile stress and **(B)** circumferential tensile stress for 75:25, 85:15, 95:05, MO, ML + P + P, and PA. Suture retention: oblique procedure for **(C)** ML + P + P and **(D)** PA. **(E)** Circumferential young modulus (MPa), **(F)** ultimate tensile stress (MPa), **(G)** maximum load (N) of ML + P + P and PA samples. (Mean ± SD) where, ns = no significant.

The mechanical properties of the ML + P + P TEVG were assessed for longitudinal and circumferential tensile stress and strain using PEUU: Gelatin blends (75:25, 85:15, and 95:05 w/w). Comparisons were made with monolayer samples from PEUU (MO) and the Native Carotid Porcine Artery (PA). In Figure 3A, it is shown that the PEUU: Gelatin blends, MO, and ML + P + P TEVG exhibit significantly higher maximum principal strains at longitudinal tensile stress compared to the PA, with a factor of 2.02 for the ML + P + P TEVG (36.1%). However, in terms of Ultimate Tensile Stress (UTS) values, there’s no statistically significant difference between ML + P + P TEVG (6.9 MPa ± 0.9 MPa) and PA (6.6 MPa ± 1.2 MPa) as shown in Figures 3A, F. This suggests that the ML + P + P TEVG could demonstrate similar mechanical resistance to native carotid arteries under physiological conditions.

In Figure 3B, the ultimate tensile circumferential stress of the ML + P + P TEVG (2.0 MPa ± 0.8 MPa) shows no statistically significant difference compared to the PA (1.9 MPa ± 0.8 MPa). Additionally, the Circumferential Young’s Modulus data in Figure 3E supports the similar mechanical behavior between ML + P + P TEVG (6.1 MPa ± 5.5 MPa) and PA (5.4 MPa ± 1.3 MPa), consistent with previous findings on PU + RGD scaffold (Choi et al., 2008). Our results align with previous assessments on Young’s Modulus and elongation at the break point with peptide incorporation like RGD for TEVG scaffolds (Antonova et al., 2019). This data suggests that the ML + P + P TEVG’s rigidity approaches that of native carotid arteries, potentially showing similar behavior under vascular compliance. Avoiding mechanical mismatches between TEVG and native arteries reduces the risk of TEVG calcification or loss of elasticity due to low shear stresses from flow boundary layer separation in areas with diameter differences (Rodriguez-Soto et al., 2022).

The suture retention analysis in Figures 3B, C indicates that control samples of porcine artery (PA) from figure 3D, withstand higher stress (2.5 MPa ± 0.6 MPa) during the oblique procedure compared to ML + P + P TEVG (1.64 MPa ± 0.2 MPa). However, statistical analysis shows no significant difference between both groups suggesting substantial mechanical compatibility for anastomosis between native vessels and ML + P + P TEVG. This is crucial as it implies the anastomotic region could closely mimic the native microenvironment, despite vascular graft implantation (Schiller et al., 2010; Furdella et al., 2021). Figure 3G further supports the mechanical comparability between native arteries (PA) and ML + P + P TEVG, showing no statistically significant difference in maximum load between the two groups.

### 3.3 Physicochemical characterization of the ML + P + P TEVG

**FIGURE 4:**
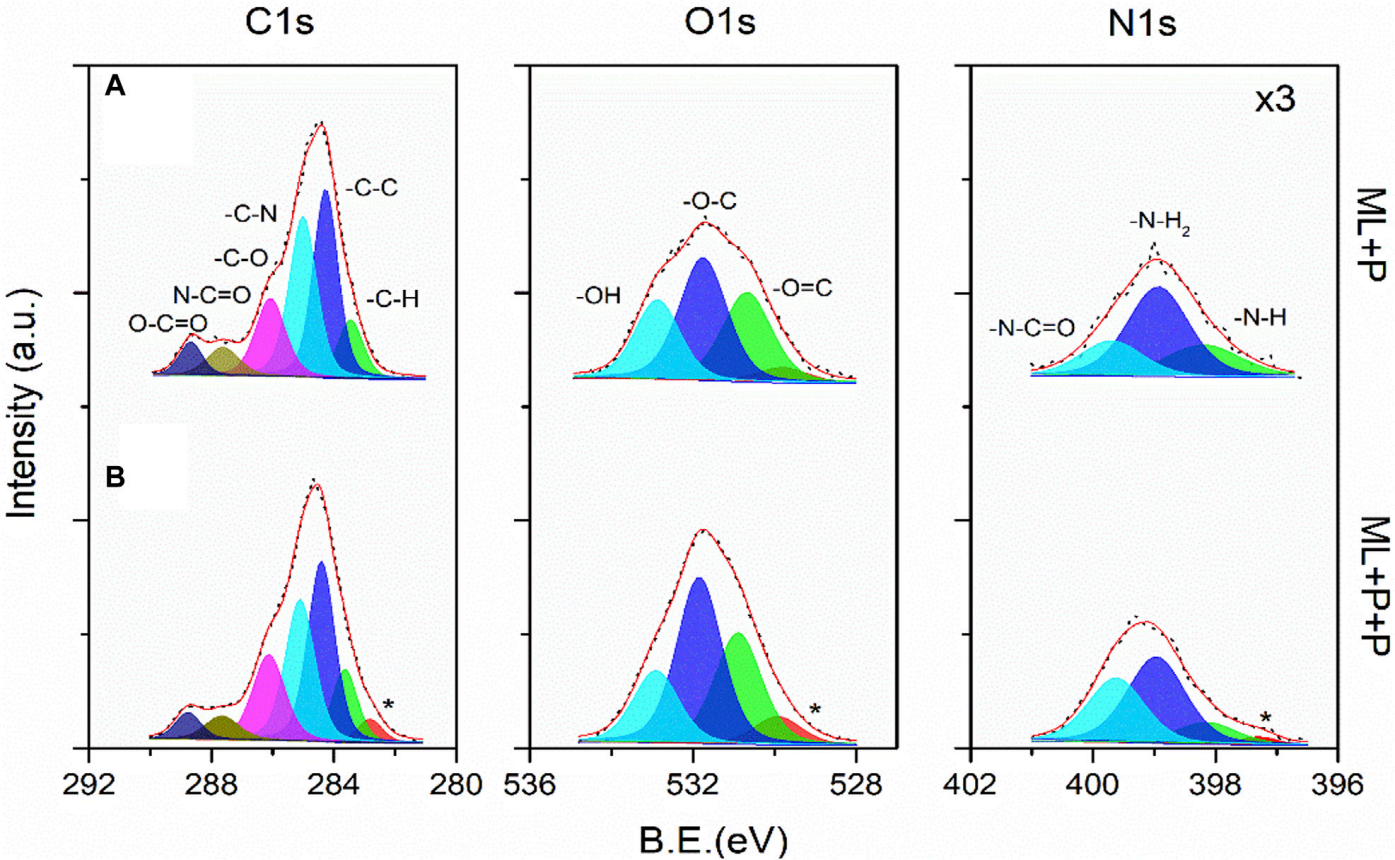
High-resolution XPS spectra for the C1s, O1s and N1s core-level of the Luminal surface of the multilayered TEVG. Comparing the **(A)** ML + P and **(B)** ML + P + P samples. *Loss peak. The binding energies (B.E.) of the C1s, O1s, and N1s core-levels of the Luminal surface of the multilayered TEVG (Supplementary Table S4) were obtained from the integration of peaks shown in **(A)** and **(B)**.

The surface chemistry characterization of ML + P + P TEGV was evaluated from the high-resolution XPS spectra of the Lumina layer as shown in Figure 4. This XPS analysis was initially conducted to the surface modification of PEUU-COOH with PEG 4 Arm NH_2_ molecules (ML + P), and subsequently, the incorporation of the RGD/SV peptides to the ML + P backbone (ML + P + P), Figures 4A, B, respectively. The evaluation of the first functionalization was presented in a previous work (Rodríguez-Soto et al., 2023). And henceforth, the main peaks were decomposed under the same fitting protocol and the corresponding sub-peaks featured the same-colored art for simplicity. The binding energies (B.E.) of the C1s, O1s, and N1s core-levels of the luminal surface of the multilayered TEVG from high-resolution XPS spectra peaks integration are presented in Supplementary Table S4. From above, the successful functionalization of the PC sample with PEG 4 Arm NH_2_ was evidenced mainly by the noticeable increase of the nitrogen counts calculated from the area under the peak for N1s core-level. Once the first functionalization was done, the study focused on the functional groups present in the outer surface layer of the polymer compound after the modification with both peptides. The relative atomic ratio of the carbon species from the C1s sub-peaks is consistent with a larger chain polymer for the ML + P + P where charging artifacts were evidenced as asymmetric tails at lower energies. This result was expected as a consequence of the modification of the chemical potential of the ML + P sample surface with a larger chain extension coming from a less conductive ML + P + P polymer. Then, differences in sub-peaks contributions were mainly observed from O1s and N1s core-levels. For the O1s peak, the concentration of the–O=C and–O-C functional groups increased for the ML + P + P sample and this fact was corroborated by the more pronounced N1s sub-peak linked to the–N-C=O amide group. The chemisorbed hydroxyl and amine species were kept in a coherent range of atomic percentages for the modified polymer and there was no evidence of protonated nitrogen species along the chain. In this way, the luminal layer formation from the *in situ* functionalization of the ML + P sample with RGD/SV peptides was corroborated.

**FIGURE 5:**
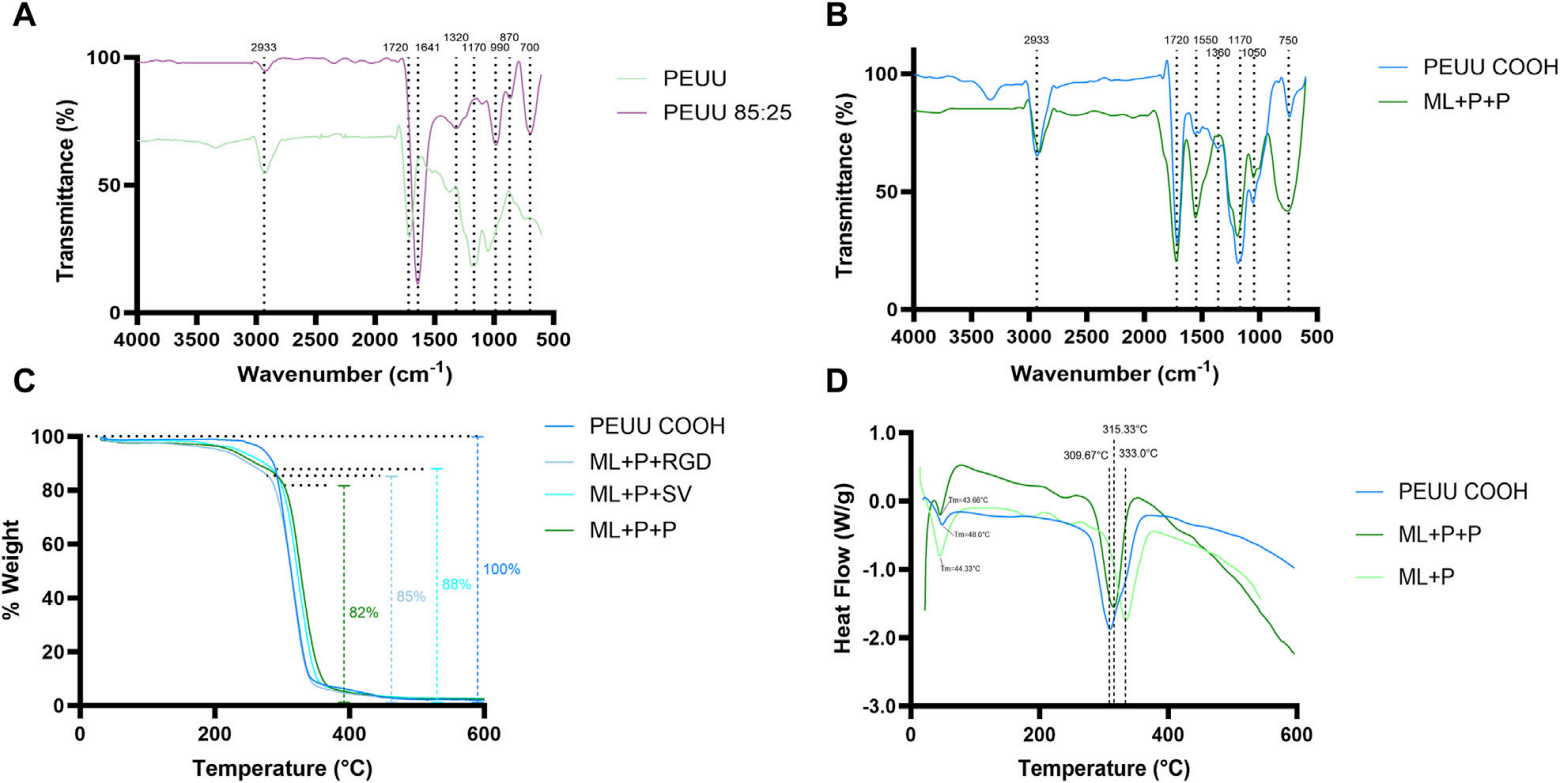
Physicochemical evaluation. FTIR of **(A)** PEUU and PEUU COOH Gelatin B 85:24, and **(B)** PEUU COOH and ML + P + P. **(C)** TGA analysis of PEUU COOH, PEUU COOH + PEG + SV (ML + P + SV), and PEUU COOH + PEG + RGD (ML + P + RGD). **(D)** DSC analysis of PEUU COOH, ML + P + P, PEUU + PEG four arm (ML + P).

FTIR analysis presented in Figure 5A confirmed the gelatin B incorporation into PEUU. Additionally, the incorporation of PEG, RGD, and SV peptides into PEUU was evaluated using the same technique, comparing PEUU COOH samples with ML + P + P samples, as presented in Figure 5B. TGA analysis determined the thermal degradation profile and the effectiveness of surface functionalization of PEUU-COOH + PEG + Peptides (SVV and RGD) shown in Figure 5C. DSC evaluation characterized differences in heat capacity, glass transition temperature, crystallization temperature, and melting temperature between PEUU-COOH, PEU + PEG, and ML + P + P samples as shown in Figure 5D, confirming the functionalization of PEG and peptides in PEUU samples. These analyses build upon our previous work characterizing PEUU and PEUU-COOH as potential biomaterials for TEVG applications (Rodríguez-Soto et al., 2023).

Figure 5A shows the incorporation of Gelatin B through a peak at 1,641 cm^−1^ for PEUU 85:25, comparable to the PEUU peak at 1,720 cm^−1^. This displacement in wavelength could be due to gelatin, which typically exhibits peaks at 1,630 and 1,650 cm^−1^, corresponding to amide-I presence (Wang et al., 2002). Additionally, PEUU 85:25 displays a peak at 1,320 cm^−1^, absent in the PEUU sample alone. This peak is likely due to gelatin incorporation, as reported previously, with gelatin showing characteristic peaks within 1,235 and 1,450 cm^−1^ (Sizeland et al., 2018). Characteristic peaks are observed on PEUU 85:25 around 990 and 700 cm^−1^. Similar peaks have been identified in porcine and bovine gelatin samples within the wavenumber range of 710–740 cm^−1^ and 1,030–1,080 cm^−1^ in the fingerprint region. These peaks are attributed to C-H vibration and C-S stretching on methyl-sulfide or aliphatic disulfide, as well as to aromatic ring vibration or stretching vibrations of C-O-C from saturated aliphatic carboxylic acid.

The peak at 135 observed on PEUU 85:25 at 1,170 cm^−1^ is attributed to the incorporation of gelatin into the PEUU. This is supported by a peak at 1,774 cm^−1^ associated with PEU surface conjugation to gelatin, representing the mesyl (sulfonyl) groups (Wang et al., 2002). Since these peaks are only observed in the PEUU 85:25 curve, the FTIR results indicate successful gelatin B incorporation.

In Figure 5B, a prominent peak at 1,550 cm^−1^ is observed for ML + P + P, which is not present in the PEUU-COOH spectrum. This peak corresponds to the Amide II bond (∼1,550 cm^−1^), primarily found in RGD (Arginine-Glycine-Aspartic Acid) and SVV peptides (Shin et al., 2017). The bands associated with this peak signify stretching and bending vibrations within the peptide bond (C=O and N-H), characteristic of proteins and peptides (Mallamace et al., 2015; Sizeland et al., 2018).

According to previous TGA reports on RGD, significant weight losses are observed between 310°C–380°C (Shin et al., 2017), which align with the observations for the PEUU-COOH + PEG + RGD curve in Figure 5C. Further assessment of thermal behavior was conducted using DSC thermograms for PEUU, PEUU-COOH, PEUU + PEG (4 ARM), and PEUU + PEG (4 ARM) + Peptides, as depicted in Figure 5D. The crystallization and melting behavior of PEUU were consistent with findings from previous DSC studies (Jaisankar et al., 2013; Go et al., 2016; Jahid et al., 2018). Notably, PEUU + PEG exhibited a melting temperature of 44.33°C, closely approximating the reported Tm for PEU + PEG scaffolds (47.9°C). This can be attributed to the increased molecular weight and crystallization of PEG (Mallamace et al., 2015) The temperature differences observed at 309.6°C for PEUU-COOH, 330°C for PEUU + PEG, and 315.3°C for ML + P + P can be attributed to the molecular weight disparities among the samples. These findings align with the TGA results, which revealed an 18% weight discrepancy between PEUU-COOH and ML + P + P.

To gain a deeper understanding of the fundamental mechanisms governing *in situ* tissue regeneration facilitated by biodegradable Tissue-Engineered Vascular Grafts (TEVGs), it is crucial to explore the intricate processes underlying this phenomenon. At the core of this mechanism lies a delicate balance between graft degradation, erosion, and the subsequent generation of extracellular matrix (ECM). To elucidate this complex process within the context of the ML + P + P TEVGs, a comprehensive analysis was conducted, including both *in-vitro* and *in silico* assessments of biodegradability, with a specific focus on enzymatic and hydrolytic degradation processes.

**FIGURE 6:**
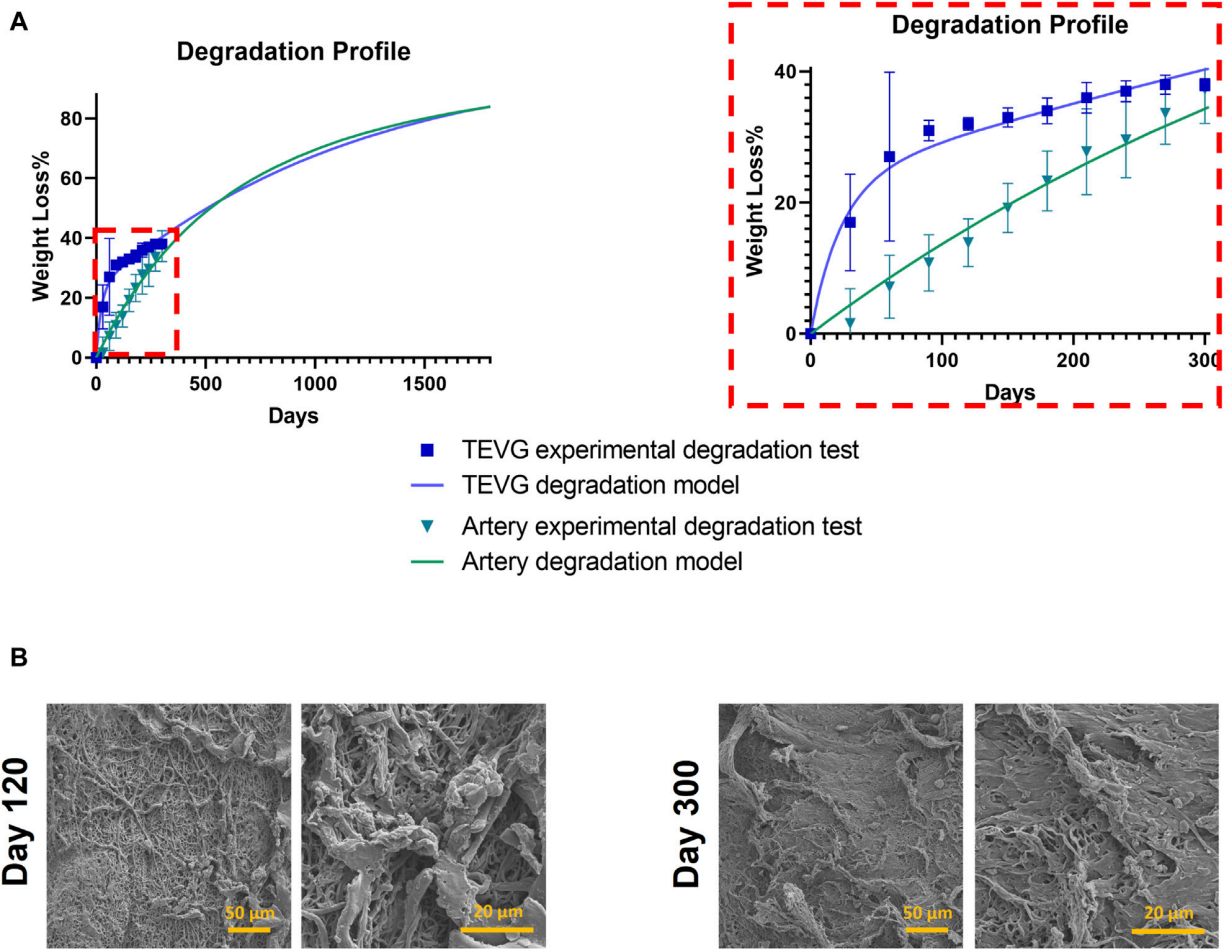
Degradation profile. **(A)** Left: Experimental data coupled with a mathematical model for overall degradation profile of ML + P + P TEVG and Native Artery. Right: Close up of experimental data on a mass loss model. **(B)** SEM images of degraded scaffolds.

We used a mass loss model crucial experimental data, forming the basis for a developed mathematical model. This model established a physics-based framework, enabling a deeper exploration of biodegradation dynamics within tissue regeneration, illustrated in Figure 6A. The artery degraded by an average of 37% over 300 days, albeit slower than the graft.

A significant observation during the final months of the study was the differential degradation rates between the native artery and the tissue-engineered vascular grafts (TEVGs). The artery exhibited a consistent linear degradation pattern, whereas the TEVGs demonstrated a multiphase degradation process, an initial rapid degradation phase followed by a slower, more gradual decline. This multiphase degradation in TEVGs is attributable to the incorporation of both gelatin and PEUU.

Each layer within the TEVGs is defined by specific gelatin to PEUU ratios, influencing both its structural thickness and degradation rate. Notably, layers with higher gelatin concentrations (e.g., 85:15 and 75:25) are thinner and degrade more rapidly compared to those with lower concentrations of gelatin (e.g., 95:5 and 100:0), which are thicker and degrade more slowly. This inverse relationship between gelatin concentration and layer thickness plays a critical role in the degradation dynamics of the graft. The structural integrity and functional longevity of the grafts are thus closely tied to these variations in layer thickness and material concentration, highlighting the complexity of the TEVG’s design and its impact on performance.

A Multiphysics model (Equations 1–15 of Supplementary Methods 3.4.2) was developed to project long-term behavior of both TEVGs and the artery. This model coupled diffusion analysis within half-pores with a degradation model considering hydrolysis, enzymatic degradation, and Reactive Oxygen Species (ROS) effects within the graft. Model parameters were calibrated independently using weight loss data from TEVGs and the artery, as shown in Figure 6A. To evaluate the model’s predictive accuracy, a Python code was created to compute the Mean Squared Error (MSE) and Mean Absolute Error (MAE) metrics. The TEVGs showed MSE values of 0.0042 and an MAE of 0.0438, while the artery exhibited MSE values of 0.0013 and an MAE of 0.0336. These results confirmed the model’s ability to accurately fit the experimental data, validating its predictive capabilities over extended periods.

The derived mathematical models project that after around 550 days, the arteries’ degradation will exceed that of the TEVGs. By approximately 1,700 days, both the TEVGs and the graft are expected to reach a degradation level of 83%. This pattern of initial rapid degradation followed by a gradual slowdown in TEVGs is likely due to the greater variability in their layer properties compared to the more uniform characteristics of artery layers. Future work will involve separate degradation tests for each graft layer to validate this hypothesis. Figure 6B provides Scanning Electron Microscopy (SEM) images for evaluating TEVGs at specific time points (120 days and 300 days), aiding in understanding the dynamic changes within these vascular grafts.

Furthermore, the SEM image captured at 120 days revealed a discernible transformation in the fiber structure in comparison to the 300-day counterpart. While the fiber structure at 120 days is not entirely pristine, it demonstrates superior organization and alignment when contrasted with the image obtained at 300 days. This initial image serves as a fundamental reference point for assessing the development of the graft’s structural integrity over time. In contrast, the 300-day image exhibits a notable departure from the earlier 120-day depiction. In the latter image, TEVG graft fibers display conspicuous tearing and fragmentation, indicating a marked reduction in structural integrity over the extended implantation period. This degradation raises questions regarding the long-term durability and suitability of the graft for clinical applications.

### 3.4 Biocompatibility and hemocompatibility of the ML + P + P TEVG

The ML + P + P fabrication process requires the use of organic solvents and potentially harmful chemicals. Rigorous evaluations were conducted to ensure TEVG cytocompatibility and hemocompatibility. These assessments focused on verifying proper chemical quenching and device safety, as well as evaluating the anti-thrombogenic properties conferred by PEG 4 arm NH2. Tests included cell viability assessments with HUVEC, THP-1, and L929 cell lines, as well as examinations of hemolysis rates and coagulation dynamics.

**FIGURE 7:**
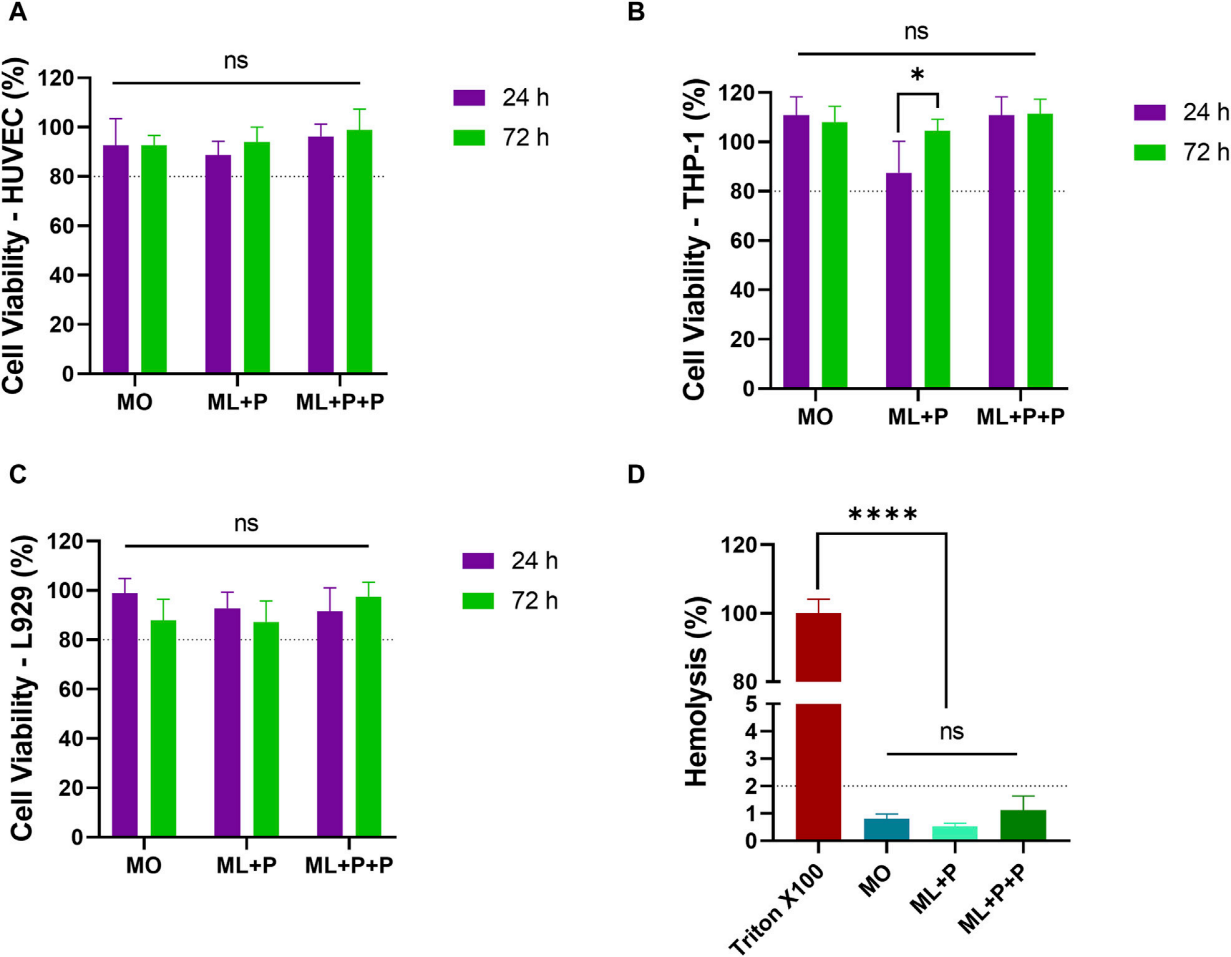
ML + P + P Biocompatibility. Cell viability of various cell lines exposed to the ML + P + P TEVG was analyzed at both 24 h and 72 h, **(A)** HUVECs, **(B)** THP-1, **(C)** L929. **(D)** Hemolysis percentages of erythrocytes directly exposed to the ML + P + P TEVG and control samples. (Mean ± SD) where, ns = no significant *p ≤ 0.05, **p ≤ 0.01, ***p ≤ 0.001, ****p ≤ 0.0001.

The viability of cells exposed to ML + P + P TEVG and control samples was assessed at 24 and 72 h, compared with MO and ML + P samples. Figures 7A–C shows no significant viability decrease for any cell type, all surpassing the ISO 10993 threshold of 80%. Specifically, ML + P + P TEVG’s viabilities were: HUVECs—96.2% ± 5.1 at 24 h and 99.0% ± 8.4 at 72 h; THP-1s—110.8% ± 7.4 at 24 h and 111.3% ± 6.1 at 72 h; L929 cells—91.5% ± 9.6 at 24 h and 97.4% ± 5.9 at 72 h. Hemolysis rates for all groups stayed below 5%, with ML + P + P TEVG notably at 1.1% ± 0.5 as shown in Figure 7D.

**FIGURE 8:**
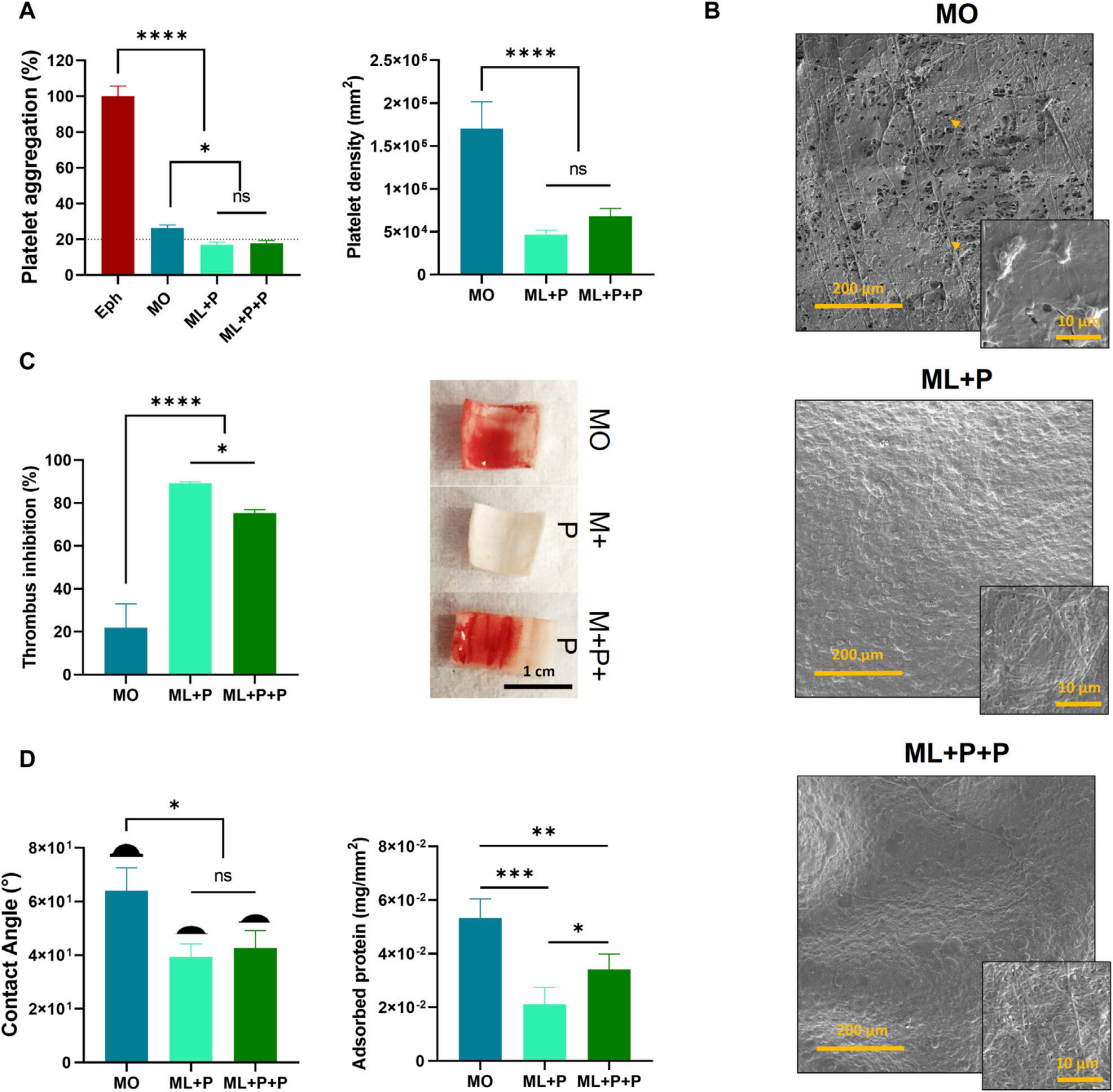
ML + P + P Antithrombotic properties. **(A)** platelet aggregation percentage induced by samples on platelet-rich plasma (PRP) and platelet density of cells adhered to the sample surfaces, **(B)** SEM images of samples exposed to PRP and platelet morphology, **(C)** thrombus inhibition percentage and images showing whole blood clot formation on sample surfaces, **(D)** contact angle and adsorbed protein on sample surfaces. (Mean ± SD) where, ns = no significant *p ≤ 0.05, **p ≤ 0.01, ***p ≤ 0.001, ****p ≤ 0.0001.

We assessed the ML + P + P TEVG’s anti-thrombotic properties by analyzing induced platelet aggregation using PRP, platelet density on sample surfaces, and thrombus inhibition in whole blood. This was correlated with contact angle and protein adsorption. Figure 8A shows ML + P + P TEVG had significantly lower platelet aggregation percentages compared to MO (17.9% ± 1.5% vs. 26.5% ± 5.8). Platelet density on ML + P + P TEVG was notably lower than MO (6.8 × 10^4^ ± 9.4 × 10^3^ platelets/mm^2^ for ML + P + P vs. 1.7 × 10^5^ ± 3.1 × 10^4^ platelets/mm^2^ for MO), with no notable differences between ML + P and ML + P + P TEVG behaviors.

Figure 8B SEM images reveal active platelet adhesion on the MO sample, contrasting with platelet-free ML + P and ML + P + P surfaces. This supports the TEVG’s anti-platelet activity, likely due to steric inhibition from PEG 4 arm NH_2_, despite adhesive peptides on its surface (Rodríguez-Soto et al., 2023). Surface properties, such as smoothness and hydrophilicity, notably influence platelet deposition, reducing aggregation and thrombosis probabilities (Goodman and Albrecht, 1987). Figure 8C shows thrombus inhibition percentages for the samples. ML + P exhibited 89.3% ± 0.6 inhibition, slightly reduced to 75.4% ± 1.6 for ML + P + P. Both notably outperformed the non-functionalized MO sample at 21.8% ± 11.2.

Figure 8D correlates the results with contact angle, showing reduced angles due to PEG 4 arm NH_2_ functionalization (64.0° ± 8.5 for MO vs. 39.3° ± 4.9 for ML + P and 42.7° ± 6.5 for ML + P + P). Protein adsorption profiles differed: ML + P and ML + P + P had lower adsorbed proteins than MO, with ML + P + P slightly higher than ML + P (5.3 × 10^−2^ ± 7.1 × 10^−3^ mg/mm^2^ for MO, 2.1 × 10^−2^ ± 6.2 × 10^−3^ mg/mm^2^ for ML + P, and 3.4 × 10^−2^ ± 5.8 × 10^−3^ mg/mm^2^ for ML + P + P). This discrepancy may be attributed to the fact that the RGD peptide is a fibrinogen-binding motif, within the Aα chain of the platelet integrin, αIIbβ_3_, implicated in platelet aggregation (Adamson et al., 2018).

### 3.5 The ML + P + P TEVG provides endothelialization potential

The absence of endothelialization is a significant issue in small-diameter vascular graft failure. Under normal physiological conditions, endothelial cells play a crucial role in regulating the behavior of platelets and inflammatory cells, mainly due to their release of NO, which serves as a direct inhibitor for both types of cells, thereby preventing thrombogenesis. The presence of an endothelial lining acts as a deterrent to other complications such as intimal hyperplasia and atherogenesis. While peptides like REDV target endothelial cells specifically (Mahara et al., 2021) our study takes an approach with the ML + P + P TEVG functionalized with RGD aiming to capture a broader spectrum of endothelial progenitor cells (EPCs) circulating in the bloodstream. Considering the cell heterogeneity of their precursors, EPCs exhibit a diverse range of origins, from hematopoietic stem cells, mesenchymal stem cells, and a distinct myeloid origin (Rodriguez-Soto et al., 2022). Consequently, the RGD peptide was incorporated into the polymer functionalization on the TEVG ML + P + P lumen, owing to its potential to promote cell adhesion through its hydrophilic amino acid residues that interact with integrins α_5_β_1_, α_v_β_3_, and α_v_β_5_ of the cell membrane (Ren et al., 2015).

Furthermore, transitioning from endothelial progenitor cells (EPCs) to mature endothelial cells (ECs) is desired for achieving the modulatory effects of the endothelium in the context of TEVG regeneration. Although the maturation of endothelial cells is mainly dependent on the dynamic mechano-signaling provided by pulsatile flow in laminar hemodynamics, the SV peptide from osteopontin was introduced as a strategy to induce maturation of the attached endothelial cells, since it fosters the polarization and differentiation of these cells, thereby potentiating their angiogenic activity (Hamada et al., 2003). This has been shown to augment cell adhesion and migration (Tanaka et al., 2020).

We thoroughly investigated the pro-endothelialization attributes of the ML + P + P TEVG by analyzing cellular structure with phalloidin staining and SEM. We also quantified DNA content, examined RNA expression, and measured ROS generation and released molecules.

**FIGURE 9:**
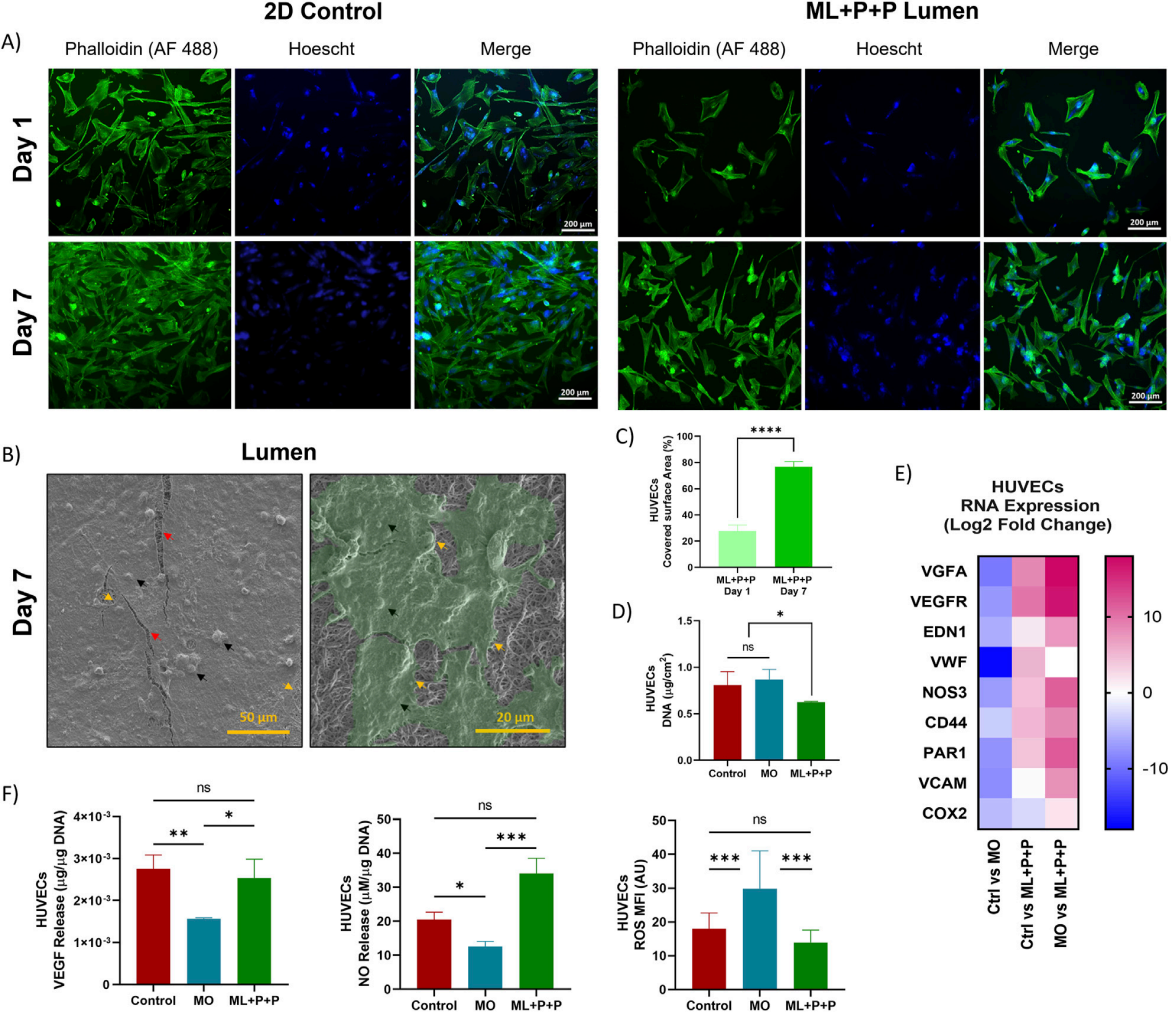
Endothelialization potential of ML + P + P with HUVECs seeded on the luminal surface. **(A)** Phalloidin staining at days 1 and 7 compared with a 2D control on a glass slide. **(B)** SEM images of Endothelial cell lining. Black arrows highlight cells and cell nuclei, yellow arrows indicate cell boundaries, and red arrows correspond to cracks in the fixed cell monolayer resulting from sample processing; beneath this layer, electrospun fibers are visible. **(C)** Percentage of covered surface area by HUVECs, data normalized from with 2D control. **(D)** DNA quantification at 7 days. **(E)** RNA expression profile. **(E)** VEGF and NO release. **(F)** Intracellular ROS production. (Mean ± SD) where, ns = no significant *p ≤ 0.05, **p ≤ 0.01, ***p ≤ 0.001, ****p ≤ 0.0001.

Figure 9A shows HUVECs stained with phalloidin on the ML + P + P TEVG luminal surface and a 2D control on a glass slide. Initially, the covered area on the structure is smaller than the control on day 1. However, by day 7, HUVECs had proliferated and covered a larger portion of the luminal surface uniformly, as displayed in Figure 9B and quantified in Figure 9C. In fact, compared to the 2D control at day 1 the cell covered surface on the ML + P + P TEVG was only 27.7 ± 4.5% which increased to 76.7 ± 4.0% at day 7. SEM images in Figure 9B reveal fissures in the fixed cell monolayer, exposing the electrospun fibers beneath. DNA content on the ML + P + P (6.3 × 10^−1^ ± 8.5 × 10^−3^ μg/cm^2^) is lower than the control (8.1 × 10^−1^ ± 1.5 × 10^−1^ μg/cm^2^) or MO sample (8.7 × 10^−1^ ± 1.1 × 10^−1^ μg/cm^2^) as seen on Figure 9D. This might be linked to the inhibitory action of the PEG 4 arm NH_2_ on endothelial cell adhesion (van Almen et al., 2016), as PEG’s highly hydrophilic properties deter the binding of cell adhesion mediators (Chuang and Masters, 2009) to the graft surface (Hao et al., 2020).

Furthermore, RNA expression analysis of endothelial cells on the TEVG (Supplementary Table S5) revealed intriguing behavior. Compared to the 2D control, the MO sample exhibited a notable reduction in all gene expressions, particularly in VWF (−17.5 ± 2.0), VEGFA (−9.2 ± 1.4), VCAM (−8.0 ± 1.6), and VEGFR (−7.1 ± 1.2) as depicted in Figure 9E. Conversely, the ML + P + P TEVG showed an increase in markers associated with HUVEC proliferation such as VEGFA (8.5 ± 1.0) and VEGFR (9.7 ± 1.5), which was further pronounced when compared to the MO sample’s changes in VEGFA (17.8 ± 1.0) and VEGFR (16.7 ± 1.5). These results could be attributed to the vasculogenic effects of the RGD peptide (Yaralı et al., 2020).

Moreover, ML + P + P showed an increase in the Log2 fold change expression of genes such as VWF (5.4 ± 0.2 vs. 2D control and 22.9 ± 0.2 vs. MO sample), NOS3 (4.4 ± 0.8 vs. 2D control and 11.4 ± 0.8 vs. MO sample) and PAR 1 (4.1 ± 1.1 vs. 2D control and 11.6 ± 1.1 vs. MO sample), all markers of endothelial maturation (Rodriguez-Soto et al., 2022), suggesting at the SV peptide’s efficacy in inducing functional endothelial phenotypes. This pattern is also confirmed in Figure 9F, where despite hosting fewer cells than the 2D control, ML + P + P TEVG released higher levels of NO (34.1 ± 4.4 µM NO/µg DNA vs. 20.6 ± 2.1 µM NO/µg DNA), a key marker on endothelial function and the main regulator of platelet interaction and inflammatory responses (Boughaleb et al., 2022). Besides, the VEGF release from the ML + P + P TEVG displayed a non-statistically significant difference in protein release into the cell media compared to the 2D control (2.5 × 10^−3^ ± 4.5 × 10^−4^ μg VEGF/µg DNA vs. 2.8 × 10^−3^ ± 3.3 × 10^−4^ μg VEGF/µg DNA), indicating the preservation of functional HUVEC phenotypes. Intracellular ROS (Figure 9E; Supplementary Figure S3), indicative of cell stress, was significantly decreased in the ML + P + P TEVG compared to the 2D control (14.0 ± 3.6 MFI vs. 18.0 ± 4.7 MFI), a desirable outcome because intracellular ROS can induce endothelial dysfunction, leading to a reduced NO availability due to NO degradation by superoxide anions. Consequently, the ensuing peroxynitrite induces protein nitration, contributing to impaired cell activity (Incalza et al., 2018). Despite initial impairment in endothelial adhesion on the ML + P + P TEVG, the polymer functionalization proved efficacious in supporting endothelial lining formation and promoting maturation. This is evident through the evolution of a functional endothelial phenotype, as indicated by RNA gene expression and relevant biomarkers.

### 3.6 The ML + P + P TEVG promotes endothelial function in a HUVECs + THP-1 coculture

TEVG failure is often linked to inflammatory processes that mediate long-term patency loss (de Vries and Quax, 2018; Rodriguez-Soto et al., 2021). Platelets adhering to a TEVG’s surface activate and release inflammatory mediators and growth factors, triggering monocyte recruitment and differentiation into macrophages. This intensifies thrombogenesis signals and recruits more platelets, known as platelet-leukocyte aggregate formation (Hamilos et al., 2018; Pluta et al., 2022). The interaction between endothelial cells and monocytes/macrophages is crucial for the development of intimal hyperplasia and atherosclerotic lesions, potentially leading to graft wall stiffening (He et al., 2012). A functional resting phenotype is defined by minimal interaction with leukocytes, due to a reduction in adhesion molecules such as VCAM1 and basal NO production which maintains leukocyte quiescence. Deviation from these fundamental functions signals endothelial dysfunction (Pober and Sessa, 2007).

Moreover, functional endothelial cells have been shown to prompt macrophage polarization towards an M2 phenotype, establishing a mutually beneficial relationship. M2 macrophages are known to support angiogenesis (He et al., 2012). However, striking a delicate balance between promoting an acute inflammatory response for angiogenesis and cell recruitment, while avoiding the adverse effects associated with chronic M1 macrophage activation, is crucial. Chronic stimulation of M1 macrophages has been associated with disrupting endothelial cell tight junctions, potentially impeding the reendothelialization process in tissue-engineered vascular grafts and posing a potential hurdle to their efficacy (Luo et al., 2019).

Considering the functional phenotype displayed by HUVECs on the ML + P + P TEVG, we proposed an *in vitro* coculture model to assess the interaction mechanisms between endothelial cells and monocytes transitioning into macrophages. In this model, we evaluated markers of endothelial function alongside the potential for M1 to M2 macrophage polarization. Specifically, we focused on cells transitioning between M1 and M2 phenotypes, which were identified using fluorescence microscopy by their display of both green and red pixels corresponding to M1 and M2 macrophage markers, respectively.

**FIGURE 10:**
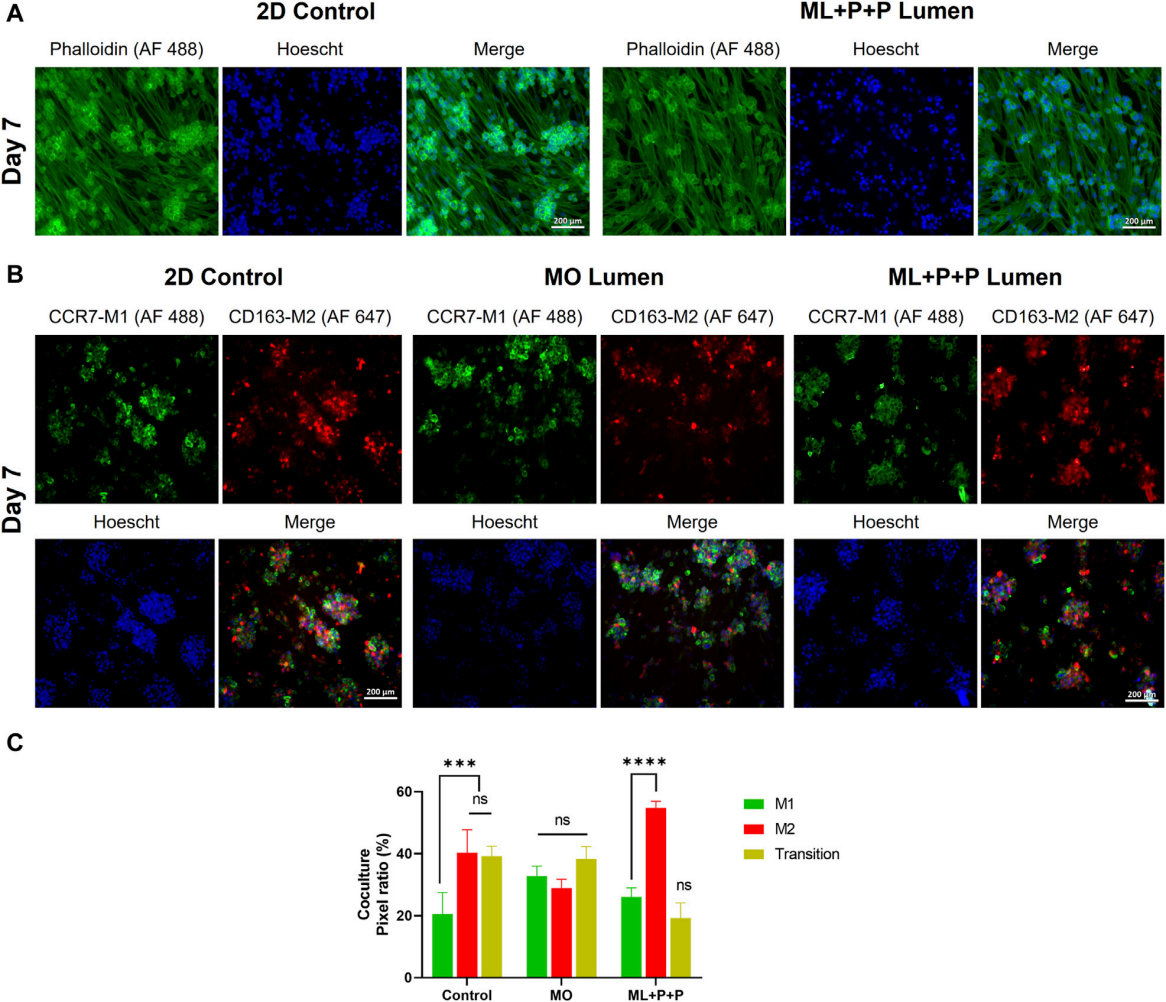
Endothelial function of ML + P + P TEVG in a HUVECs + THP-1 coculture seeded on the luminal surface. **(A)** Phalloidin staining on day 7 compared with a 2D control on a glass slide. **(B)** Immunofluorescence staining for identification M1/M2 markers (CCR7-AF 488/CD163 AF 647). **(C)** Pixel ratio percentage of cells positive for M1, M2 or expressing both markers. (Mean ± SD) where, ns = no significant *p ≤ 0.05, **p ≤ 0.01, ***p ≤ 0.001, ****p ≤ 0.0001.

Figure 10A displays phalloidin staining results in our coculture model, showing the spatial distribution of HUVECs and differentiated THP-1 cells into macrophages. Figure 10B illustrates immunofluorescence findings identifying M1/M2 markers (CCR7-AF 488 and CD163 AF 647). Figure 10C presents pixel ratio percentages for M1, M2, and dual-marker cells. In the 2D control, most cells showed M2 macrophage traits (40.3% ± 7.3) or were cells in transition (39.2% ± 3.3), with fewer M1 macrophages (20.5% ± 6.9). Conversely, ML + P + P increased M2 macrophages (54.7% ± 2.2), reduced M1 (26.1% ± 2.9), and cells in transition (19.2% ± 4.9). This contrasted with the MO sample’s even distribution (32.8% ± 3.2 M1, 28.9% ± 2.8 M2, 38.3 ± 4.0 in transition).

**FIGURE 11:**
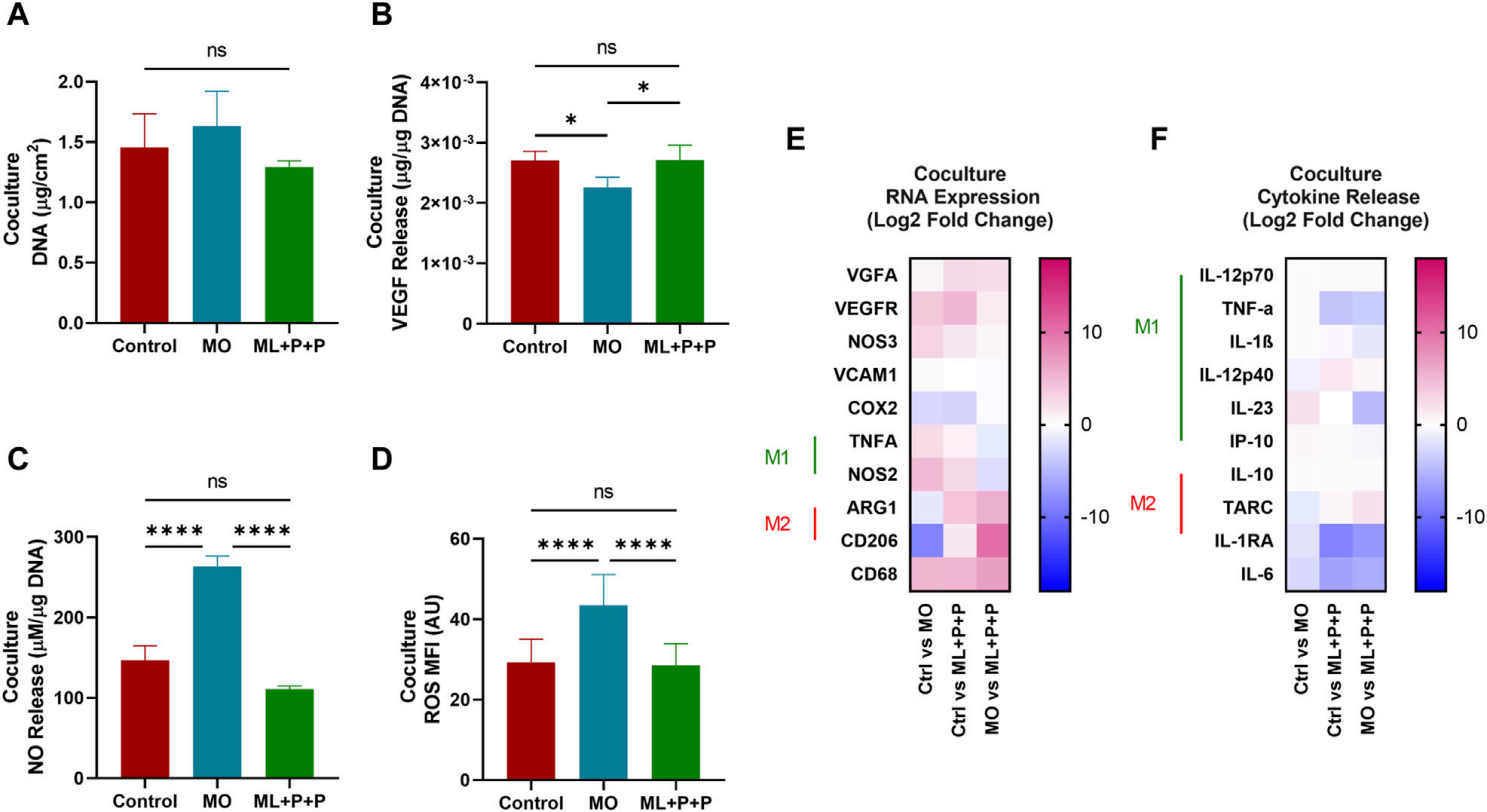
Endothelial function of ML + P + P TEVG in a HUVECs + THP-1 coculture seeded on the luminal surface. **(A)** DNA quantification. **(B)** VEGF and **(C)** NO release. **(D)** Intracellular ROS production. **(E)** RNA expression profile, **(F)** M1/M2 released cytokines on the cell media. (Mean ± SD) where, ns = no significant *p ≤ 0.05, **p ≤ 0.01, ***p ≤ 0.001, ****p ≤ 0.0001.

Figure 11A displays the DNA content per 1 cm^2^ sample in the coculture. The DNA content for ML + P + P TEVG (1.3 ± 0.1 μg/cm^2^) is comparable to both the control (1.5 ± 0.3 μg/cm^2^) and MO sample (1.6 ± 0.3 μg/cm^2^). In contrast to the lower endothelial coverage observed in HUVEC monoculture on the TEVG luminal surface, the DNA content appeared uniformly distributed across all samples, consistent with the phalloidin images. Both M1 and M2 macrophages release high levels of VEGF and angiogenic factors, crucial for enhancing monocyte recruitment (Cho et al., 2001). We hypothesized that the VEGF released by THP-1s might impact HUVEC proliferation on ML + P + P TEVGs (Cho et al., 2001).

In Figure 11B, the results indicate that the VEGF concentration released by the ML + P + P TEVG and the 2D control did not show a significant difference (2.7 × 10^−3^ ± 1.5 × 10^−4^ μg VEGFA/µg DNA vs. 2.7 × 10^−3^ ± 2.5 × 10^−4^ μg VEGFA/µg DNA), while the MO sample exhibited a notably lower VEGFA concentration (2.3 × 10^−3^ ± 1.7 × 10^−4^ μg VEGFA/µg DNA). The VEGFA released by the macrophages in our coculture model may have stimulated HUVEC proliferation until confluence was achieved on the ML + P + P TEVG.

Previous reports indicate that a concentration of 20 nM/mL (equivalent to 3 × 10^−3 ^µg/sample), as observed in the ML + P + P TEVG, doubled the HUVECs’ covered area, promoting proliferation, migration, and angiogenesis. This effect of VEGFA on endothelial cells is linked to enhanced mitochondrial oxidative respiration, increased intracellular ATP levels, and decreased ROS production due to elevated glutathione peroxidases (Guo et al., 2017). In our analysis, similar ROS levels were observed in the ML + P + P TEVG (28.6 ± 5.4 MFI) and the 2D control (29.3 ± 5.8), significantly lower than in the MO sample (43.5 ± 7.7 MFI), as shown in Figure 11D and Supplementary Figure S4. Furthermore, as seen on Figure 11C, a significant seven-fold increase in released NO was noted compared to HUVEC monoculture in both the ML + P + P TEVG and the 2D control (147.0 ± 17.9 µg NO/µg DNA vs. 111.0 ± 4.0 µg NO/µg DNA), yet significantly lower than in the MO sample (263.5 ± 12.8 µg NO/µg DNA).

The deviations observed between monoculture and coculture, along with the increased NO levels in the MO sample, may stem from the dual NO-NOS3 source from HUVECs and NOS_2_ from macrophages. This, coupled with the hyperactivated state of macrophages in the MO sample, led to heightened NO production (Mattila and Thomas, 2014). This was confirmed by the higher expression of NOS_2_ in the MO sample (4.8 ± 1.5 and 2.6 ± 1.2 Log 2-Fold change) compared to the 2D control and ML + P + P TEVG, as depicted in Figure 11E. Interestingly, there was a decline in the expression of genes related to M1 macrophages (TNFα and NOS_2_), while an increase was noted in the gene expression related to M2 macrophages (ARG1 and CD206) on the ML + P + P TEVG compared to the MO sample and 2D control. While the markers of M2 activation in the cytokine release assay did not show significant changes for the ML + P + P TEVG, it is notable that cytokines associated with M1 macrophage activation were reduced, as illustrated in Figure 11F.

Interestingly, a downward trend was observed in the expression of genes related to M1 macrophages (TNFα and NOS_2_), while an uptick was noted in the gene expression related to M2 macrophages (ARG1 and CD206) on the ML + P + P compared to the MO sample and 2D control. While the markers of M2 activation in the cytokine release assay did not exhibit a significant fluctuation for the ML + P + P TEVG, it is noteworthy that cytokines associated with M1 macrophage activation were reduced as shown in Figure 11F.

Supplementary Table S6 contains additional information on RNA expression and cytokine release. In summary, the findings reveal a dynamic interplay in the coculture model, with macrophages initially fostering endothelial growth. This transition from M1 to M2 polarization, influenced by endothelial cell function within the TEVG, underscores a crucial symbiotic relationship that could help manage inflammatory responses and facilitate healing post-TEVG implantation.

### 3.7 The ML + P + P TEVG showcases the remarkable potential for vascular wall regeneration

**FIGURE 12:**
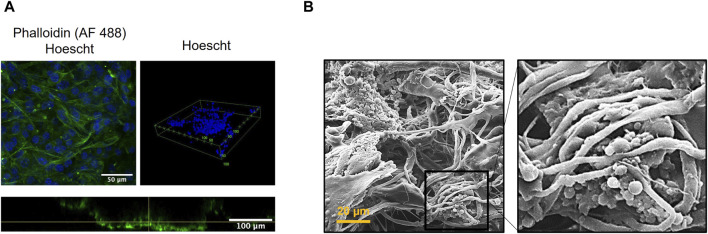
Fibroblast distribution and infiltration from the adventitial surface of the ML + P + P TEVG at 5 days. **(A)** Phalloidin staining and phalloidin distribution as reconstructed from confocal Z-stacks. **(B)** SEM images of fibroblasts infiltrating and adhering to the electrospun fibers.

Expanding on our earlier discussion, we proposed that the primary source for repopulating vascular grafts lies within the perivascular tissues directly contacting the graft’s adventitial surface. The outer layers were designed to encourage robust cell infiltration due to their high porosity. Initially, we evaluated the TEVG’s ability to support cell infiltration and growth by seeding L929 fibroblasts directly onto the adventitial surface of the ML + P + P TEVG. Figure 12 illustrates that after 5 days of culture, fibroblasts exhibit filopodia and have infiltrated the TEVG, as evidenced by phalloidin staining, phalloidin distribution from confocal Z-stacks, and SEM images demonstrating well-distributed cell populations.

In assessing the regenerative capacity of ML + P + P TEVG, it is crucial to note inflammation’s significant role in graft failure. Specifically, the balance and composition of M1 and M2 macrophages emerge as critical factors in determining vascular success or failure.

M1 macrophages are the predominant subset during the inflammatory phase, recruiting other inflammatory cells, and stem cells, and inducing angiogenesis. However, the prolonged presence of M1 macrophages leads to chronic inflammation, obstructing tissue repair. The continued presence of M1 macrophages has been associated with synthetic vascular graft failure due to fibrotic capsule formation (Dondossola et al., 2016) and may also contribute to atherogenesis through vascular wall cell calcification (Tintut et al., 2002). As initial pro-inflammatory signals decrease, anti-inflammatory signals, such as interleukin-4 (IL-4), interleukin-13 (IL-13), and transforming growth factor-beta (TGF-β), facilitate the transition from M1 to M2 macrophages, adopting an anti-inflammatory phenotype. M2 macrophages aid tissue repair and regeneration by sustaining angiogenesis, collagen deposition, and signaling to other infiltrating cells for tissue remodeling. The promotion of M2 polarization has been shown to be facilitated by pore size, interconnected pores, and adhesion molecules on scaffolds (Pham et al., 2023; Stahl et al., 2023).

Interestingly, a mere transition from M1 to M2 may not always be favorable as it could hinder cell recruitment processes and limit regeneration potential. Additionally, unique M2 induction has been linked to aortic aneurysms in rat models (Wei et al., 2019; Witherel et al., 2019). Research has demonstrated that the presence of hybrid M1/M2 macrophages, with a predominant M2 phenotype, results in fewer fibrotic processes, a less densely packed extracellular matrix (ECM) allowing better nutrient perfusion, and lower rates of calcification (Stahl et al., 2023). In *in-vivo* rat aortic interposition TEVG models, it was noted that monocytes migrated to the graft within the initial 3 days post-implantation, transforming towards an M1 phenotype that persisted throughout the regeneration process. Meanwhile, M2 macrophages appeared as early as day 7 after implantation and remained present until the study concluded at day 100. This M2 macrophage presence contributed to graft patency and correlated with regeneration indicators, including the induction of contractility and relaxation, extracellular matrix (ECM) organization, and endothelialization.

Having confirmed that the ML + P + P TEVG supports cell infiltration, we proceeded to assess the immune responses triggered by THP-1 cells seeded on the TEVG and differentiated into macrophages, analyzing their polarization potential. Our comparison included the ML + P + P TEVG, a 2D control, a MO sample, and a decellularized porcine artery (DPA) to mimic autologous vascular grafts. Autologous grafts are currently considered the gold standard for implantation due to their low immunogenicity and long-term patency.

**FIGURE 13:**
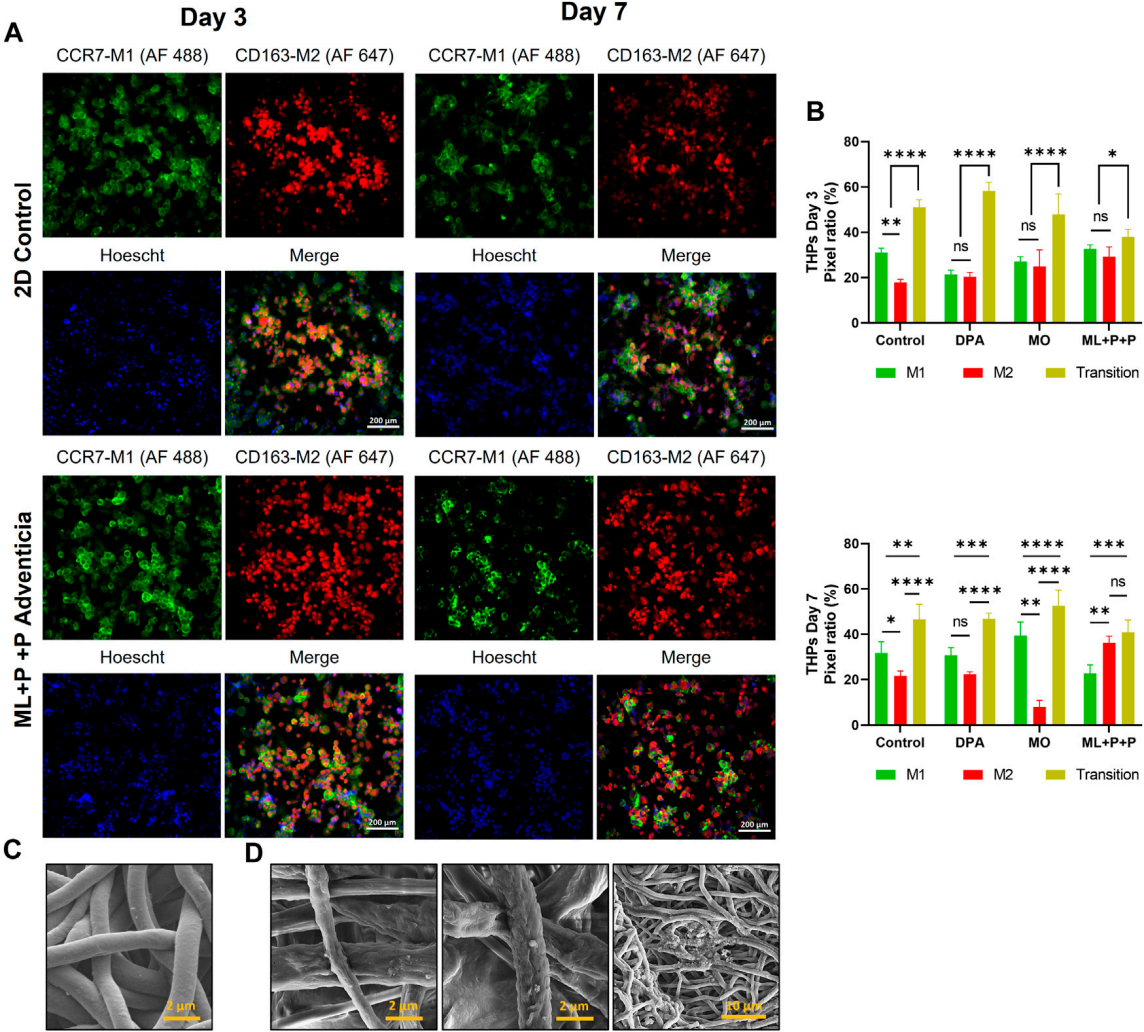
Immunomodulatory effect of the vascular wall of the ML + P + P TEVG with macrophages from THP-1 differentiation seeded on the adventitial surface. **(A)** Immunofluorescence staining for identification of M1/M2 markers (CCR7-AF 488/CD163 AF 647) on days 3 and 7 compared with a 2D control on a glass slide. **(B)** Pixel ratio percentage of cells positive for M1, M2 or expressing both markers. **(C)** SEM images of raw electrospun fibers. **(D)** SEM images of decellularized fibers after macrophage culture. (Mean ± SD) where, ns = no significant *p ≤ 0.05, **p ≤ 0.01, ***p ≤ 0.001, ****p ≤ 0.0001.

In Figure 13A, immunofluorescence staining was conducted to identify M1/M2 markers (CCR7-AF 488/CD163 AF 647) on days 3 and 7, offering insights compared to a 2D control on a glass slide. Figure 13B provides pixel ratio percentages for cells positive for M1, M2, or expressing both markers across all groups. On day 3, a common trend emerged with cells in all samples exhibiting transitional macrophage characteristics, displaying both M1 and M2 markers. Notably, the DPA sample had the highest percentage of cells in transition (58.2% ± 3.9), while ML + P + P had the lowest (38.0% ± 3.4). The proportions of cells displaying M1 or M2 markers were relatively consistent across groups. The 2D control group had a higher percentage of M1 (31.0% ± 1.9) than M2 (17.9% ± 1.4) cells, whereas the ML + P + P TEVG group showed slightly more M1 (32.7% ± 1.8) than M2 (29.3% ± 4.3) cells, though not significantly different.

Moving on to day 7, distinct patterns emerged. In the 2D control group, the M1/M2 distribution largely mirrored day 3, with a slight increase in the M2 population (21.7% ± 2.1). Conversely, the DPA group showed a decrease in transition cells (46.9% ± 2.5), with M2 remaining stable (22.4% ± 1.1 at day 7% vs. 20.4% ± 1.9 at day 3) and M1 increasing (30.8% ± 3.4 at day 7 from 21.4% ± 2.0 at day 3). The MO sample maintained consistent transition cell percentages but had a significant decrease in M2 (8.0% ± 2.9) and an increase in M1 (39.4% ± 5.9). Meanwhile, the ML + P + P group maintained a similar transition cell percentage but saw an increase in M2 (36.2% ± 2.9) and a decrease in M1 (22.8% ± 3.7) population on day 7. SEM images in Figure 13C depict the fiber structure of ML + P + P. Following macrophage culture, a decellularization process reveals a scaffold with irregular surfaces, as shown in Figure 13D. The partially degraded fibers in this figure indicate the impact of macrophage activity.

**FIGURE 14:**
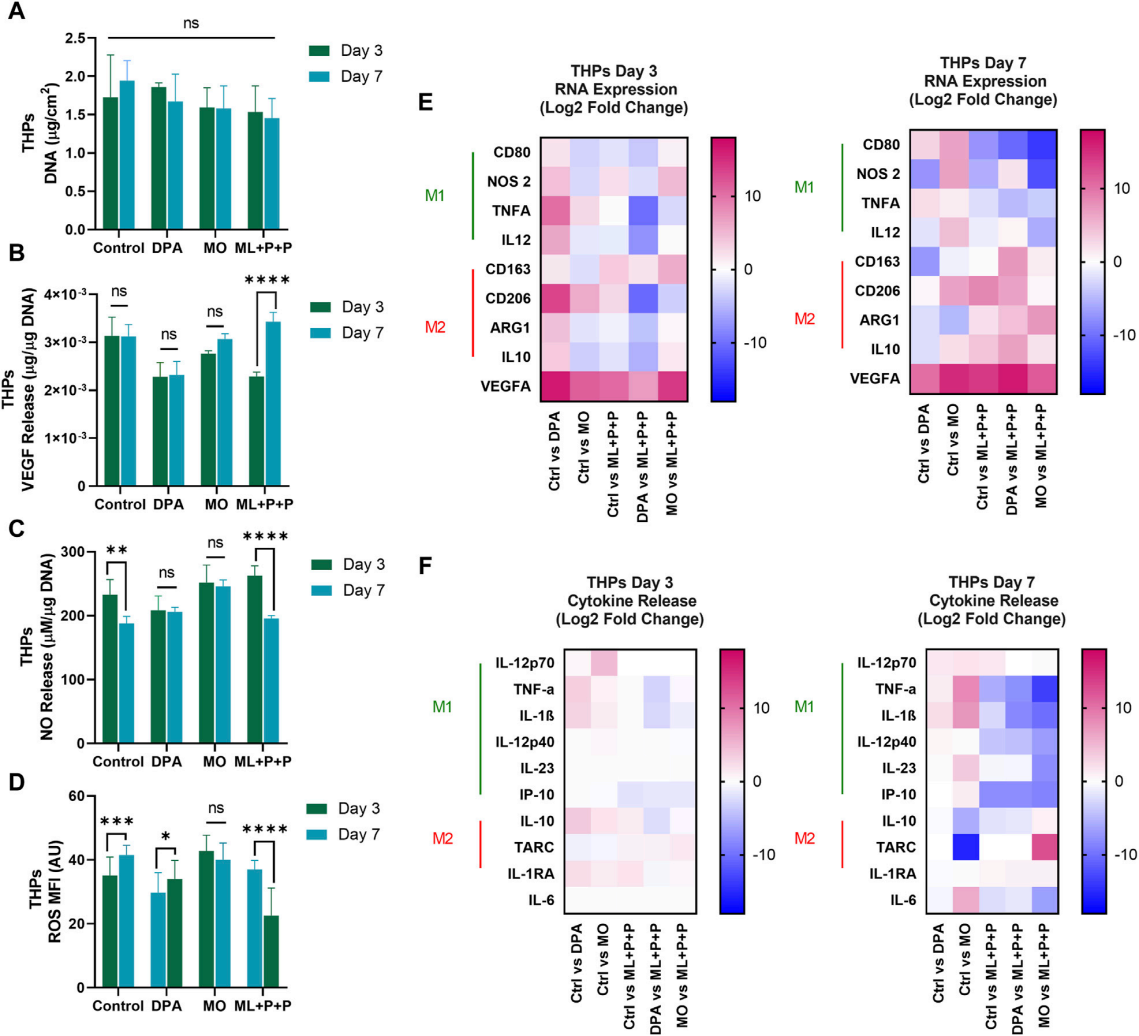
Immunomodulatory effect of the vascular wall of the ML + P + P TEVG with macrophages from THP-1 differentiation seeded on the adventitial surface. **(A)** DNA quantification. **(B)** VEGF and **(C)** NO release. **(D)** Intracellular ROS production. **(E)** RNA expression profile, **(F)** M1/M2 released cytokines on the cell media. (Mean ± SD) where, ns = no significant *p ≤ 0.05, **p ≤ 0.01, ***p ≤ 0.001, ****p ≤ 0.0001.

In Figure 14A, stable DNA content on days 3 and 7 implies that M1/M2 marker expression variations do not stem from monocyte proliferation. On day 3, both DPA cell culture (2.3 × 10^−3^ ± 3.0 × 10^−4^ μg VEGFA/µg DNA) and ML + P + P (2.3 × 10^−3^ ± 9.3 × 10^−5^ μg VEGFA/µg DNA) had lower VEGF levels than the control. However, by day 7, ML + P + P TEVG showed a notable increase (3.4 × 10^−3^ ± 1.9 × 10^−4^ μg VEGFA/µg DNA), aligning with the notion that M2 macrophages support angiogenesis, endothelization, and tissue repair as suggested in Figure 14B.

Regarding NO release, it is noteworthy that the stimulated macrophages in the 2D control on day 3 had ten times the concentration compared to resting HUVECs. DPA showed lower NO levels (208.4 ± 22.4 µM NOS/µg DNA) than ML + P + P TEVG (262.7 ± 15.5 µM NOS/µg DNA), suggesting an acute inflammatory response induced by the TEVG due to NO’s proinflammatory role (Sharma et al., 2007). However, by day 7, ML + P + P TEVG had a significantly reduced NO level (195.6 ± 4.4 µM NOS/µg DNA), as shown in Figure 14C. This contrasts with the intracellular ROS MFI trend from day 3 to day 7, where ML + P + P TEVG decreased (37.0 ± 2.8 to 22.5 ± 8.7) while the 2D control group increased (35.1 ± 5.8 to 41.5 ± 3.0), as seen in Figure 14D and Supplementary Figure S5. Both these trends, along with the M1/M2 polarization pattern, suggest inflammation resolution due to the macrostructure and gelatin inclusion in the ML + P + P TEVG. Gelatin, rich in adhesive peptides for cell adhesion, has been linked to a positive stimulus for M2 transformation (Wu et al., 2021; Pham et al., 2023). To further support these findings, gene expression analysis (Figure 14E) and cytokine panel analysis (Figure 14F) for M1/M2 were performed on days 3 and 7 (Sharma et al., 2007; Wu et al., 2021; Pham et al., 2023).

The results show that on day 3, the ML + P + P TEVG did not exhibit significant changes in gene expression or cytokine profile compared to the 2D control or other groups. However, by day 7, noticeable reductions in M1 marker expression were observed in ML + P + P, notably NOS_2_ (from 2.2 ± 0.6 to −5.6 ± 0.6 Log 2-Fold change), alongside an increase in M2 markers like ARG1 (from −0.95 ± 0.9 to 2.4 ± 0.1 Log 2-Fold change). This aligns with the decrease in NO synthesis, attributed to ARG1’s regulatory role on NOS by competing for L-arginine substrate.

The cytokine release analysis revealed a consistent trend: M1 cytokines notably decreased in ML + P + P TEVG compared to the 2D control, particularly TNFα (from 0.3 ± 0.2 to −5.7 ± 0.1 Log 2-Fold change). This reduction is in line with the decreased production of intracellular ROS, as TNFα serves as a significant inducer (Tan et al., 2016).

While there was not a significant increase in M2 markers compared to the 2D control, TARC release saw a substantial rise compared to the MO sample (from 1.4 ± 0.4 to 12.6 ± 0.0 Log 2-Fold change). This increase underscores TARC’s known role in supporting long-lived cells and facilitating their transition towards M2 phenotypes (Montella et al., 2021). For more detailed data on RNA expression and cytokine release, please refer to Supplementary Table S7 (Tan et al., 2016; Montella et al., 2021). In summary, the data indicates that ML + P + P TEVG initially triggers a pro-inflammatory response, maintaining an M1 macrophage phenotype, then transitions towards a pro-regenerative state, supporting M2 macrophage transition. This trajectory suggests potential for improved vascular graft performance and longevity, hinting at promising clinical applications.

## 4 Conclusion

A multilayered tissue-engineered vascular graft (ML + P + P TEVG) was crafted through electrospinning, combining synthetic and natural polymers to achieve dimensions suitable for vascular applications. Surface functionalization was successfully done featuring PEG 4 arm NH_2_, RGD, and SV peptides to foster endothelial cell adhesion and maturation. The integration of a gelatin gradient within the PEUU matrix showed potential in tissue remodeling and reducing pro-inflammatory cell activation. The architectural design of the graft incorporated a lower porosity and smaller pore diameters in the luminal layer to enhance blood tightness and promote cell adhesion. Conversely, the gelatin layers, facilitated cell infiltration and endorsed a favorable M2 macrophage transition. Mechanical testing revealed that ML + P + P TEVG properties are comparable to native arteries, suggesting physiological elasticity and compatibility during anastomosis. Effective quenching procedures ensured high cell viability, minimal hemolysis, and anti-thrombotic properties. ML + P + P TEVG supported elevated NO release and preserved VEGF levels, indicating maintained endothelial function, and reduced intracellular ROS levels. Immunomodulatory attributes unveiled distinct M1/M2 polarization patterns, fostering angiogenesis, endothelialization, and tissue repair. This delineation of the ML + P + P TEVG’s performance underscores its promise as a viable candidate for vascular graft applications, thereby holding substantial promise for clinical translatability in vascular tissue engineering.

## 5 Notes

The materials and structure of the TEVG here reported are protected by the patent Biodegradable, Non-Thrombogenic Elastomeric Polyurethanes. Patent Application Publication, United States. Pub. No: US 2014/0248232 A1. Pub. Date: Sep. 4, 20164. And collagen-based based multilayered regenerative vascular graft with bioactive luminal coating. (Original in Spanish: Injerto vascular regenerativo multicapa basado en colágeno, con recubrimiento luminal bioactivo). Patent application number in Colombia: NC 2021/0017700, submitted on 22 December 2021.

## Data Availability

The datasets presented in this study can be found in online repositories. The names of the repository/repositories and accession number(s) can be found in the article/Supplementary Material.
