# Corrigendum: Redefining vascular repair: revealing cellular responses on PEUU—gelatin electrospun vascular grafts for endothelialization and immune responses on *in vitro* models

**DOI:** 10.3389/fbioe.2025.1607125

**Published:** 2025-04-24

**Authors:** María A. Rodríguez-Soto, Alejandra Riveros-Cortés, Ian C. Orjuela-Garzón, Inés María Fernández-Calderón, Cristian F. Rodríguez, Natalia Suárez Vargas, Carlos Ostos, Carolina Muñoz Camargo, Juan C. Cruz, Seungil Kim, Antonio D'Amore, William R. Wagner, Juan C. Briceño

**Affiliations:** ^1^ Department of Biomedical Engineering, Universidad de los Andes, Bogotá, Colombia; ^2^ Instituto de Química, Facultad de Ciencias Exactas y Naturales, Universidad de Antioquia, Medellín, Colombia; ^3^ McGowan Institute for Regenerative Medicine and Department of Bioengineering, University of Pittsburgh, Pittsburgh, PA, United States; ^4^ Department of Congenital Heart Disease and Cardiovascular Surgery, Fundación CardioInfantil Instituto de Cardiología, Bogotá, Colombia

**Keywords:** tissue engineered vascular grafts, regenerative medicine, biomaterials, inflammatory response, immunomodulation, M1/M2 macrophage polarization, endothelialization, cell signaling

In the published article, there was an error in Figure 9 as published. The image labeled as “2D control Day 1 panel of Phalloidin (AF 488)” was mistakenly similar to the image labeled as “ML + P + P Lumen Day 7 panel of Phalloidin (AF 488) due to a mislabeling during file organization.” The corrected Figure 9 and its caption “Endothelialization potential of ML + P + P with HUVECs seeded on the luminal surface. (A) Phalloidin staining at days 1 and 7 compared with a 2D control on a glass slide. (B) SEM images of Endothelial cell lining. Black arrows highlight cells and cell nuclei, yellow arrows indicate cell boundaries, and red arrows correspond to cracks in the fixed cell monolayer resulting from sample processing; beneath this layer, electrospun fibers are visible. (C) Percentage of covered surface area by HUVECs, data normalized from with 2D control. (D) DNA quantification at 7 days. (E) RNA expression profile. (E) VEGF and NO release. (F) Intracellular ROS production. (Mean ± SD) where, ns = no significant *p ≤ 0.05, **p ≤ 0.01, ***p ≤ 0.001, ****p ≤ 0.0001.” appear below.

**FIGURE 9:**
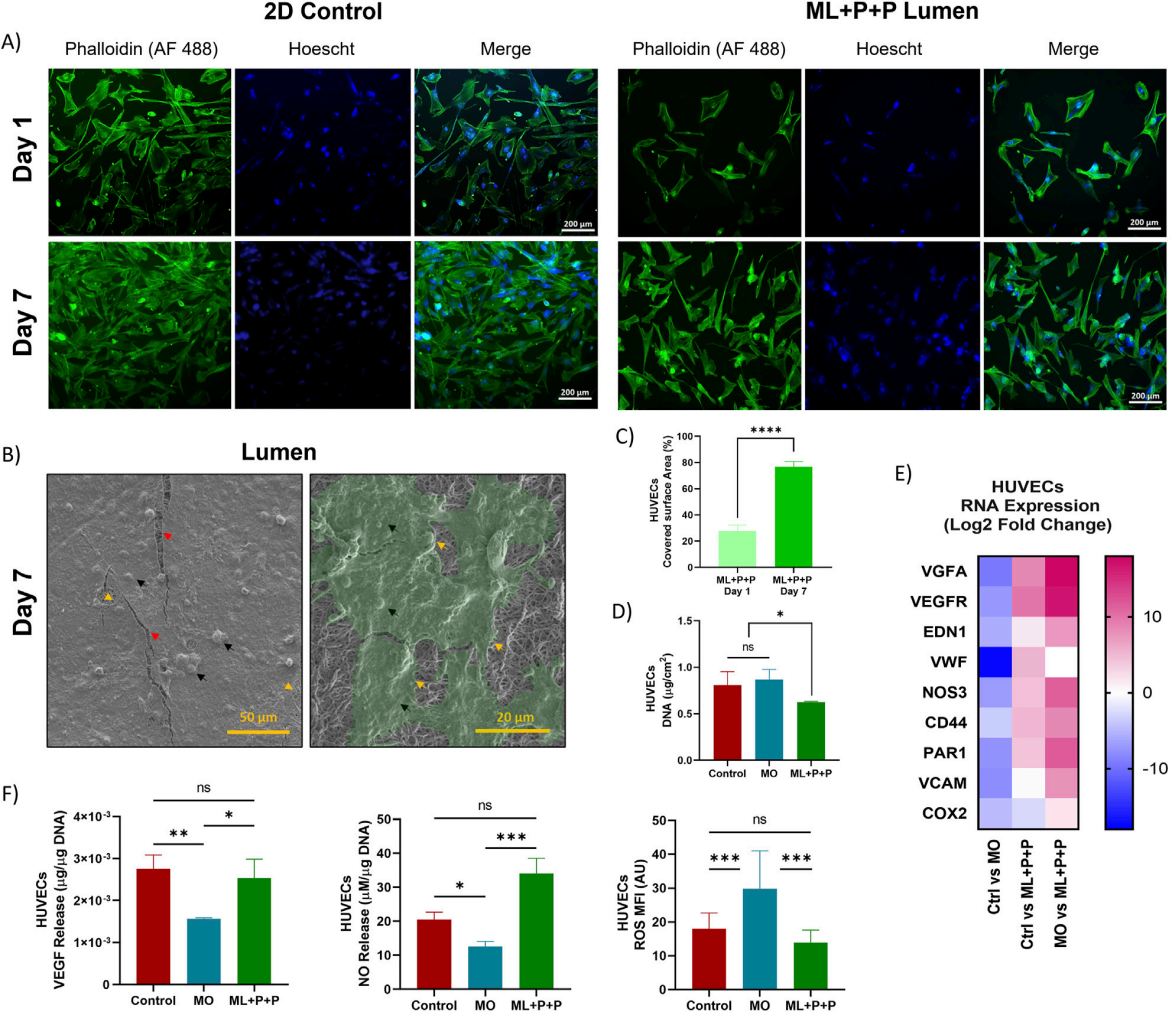
Endothelialization potential of ML + P + P with HUVECs seeded on the luminal surface. **(A)** Phalloidin staining at days 1 and 7 compared with a 2D control on a glass slide. **(B)** SEM images of Endothelial cell lining. Black arrows highlight cells and cell nuclei, yellow arrows indicate cell boundaries, and red arrows correspond to cracks in the fixed cell monolayer resulting from sample processing; beneath this layer, electrospun fibers are visible. **(C)** Percentage of covered surface area by HUVECs, data normalized from with 2D control. **(D)** DNA quantification at 7 days. **(E)** RNA expression profile. **(E)** VEGF and NO release. **(F)** Intracellular ROS production. (Mean ± SD) where, ns = no significant *p ≤ 0.05, **p ≤ 0.01, ***p ≤ 0.001, ****p ≤ 0.0001.

The authors apologize for this error and state that this does not change the scientific conclusions of the article in any way. The original article has been updated.

