# Supplementary material for: Redefining vascular repair: revealing cellular responses on PEUU—gelatin electrospun vascular grafts for endothelialization and immune responses on in vitro models
Source: Front Bioeng Biotechnol. 2024 Jun 5;12:1410863. doi: 10.3389/fbioe.2024.1410863 (PMC11188488; doi:10.3389/fbioe.2024.1410863)
Supplement: Supplementary file 1 [file DataSheet1.docx]

Supplementary Data

# Overview

**Supplementary Figure 1** Schematic representation of TEVG structure and manufacture method.


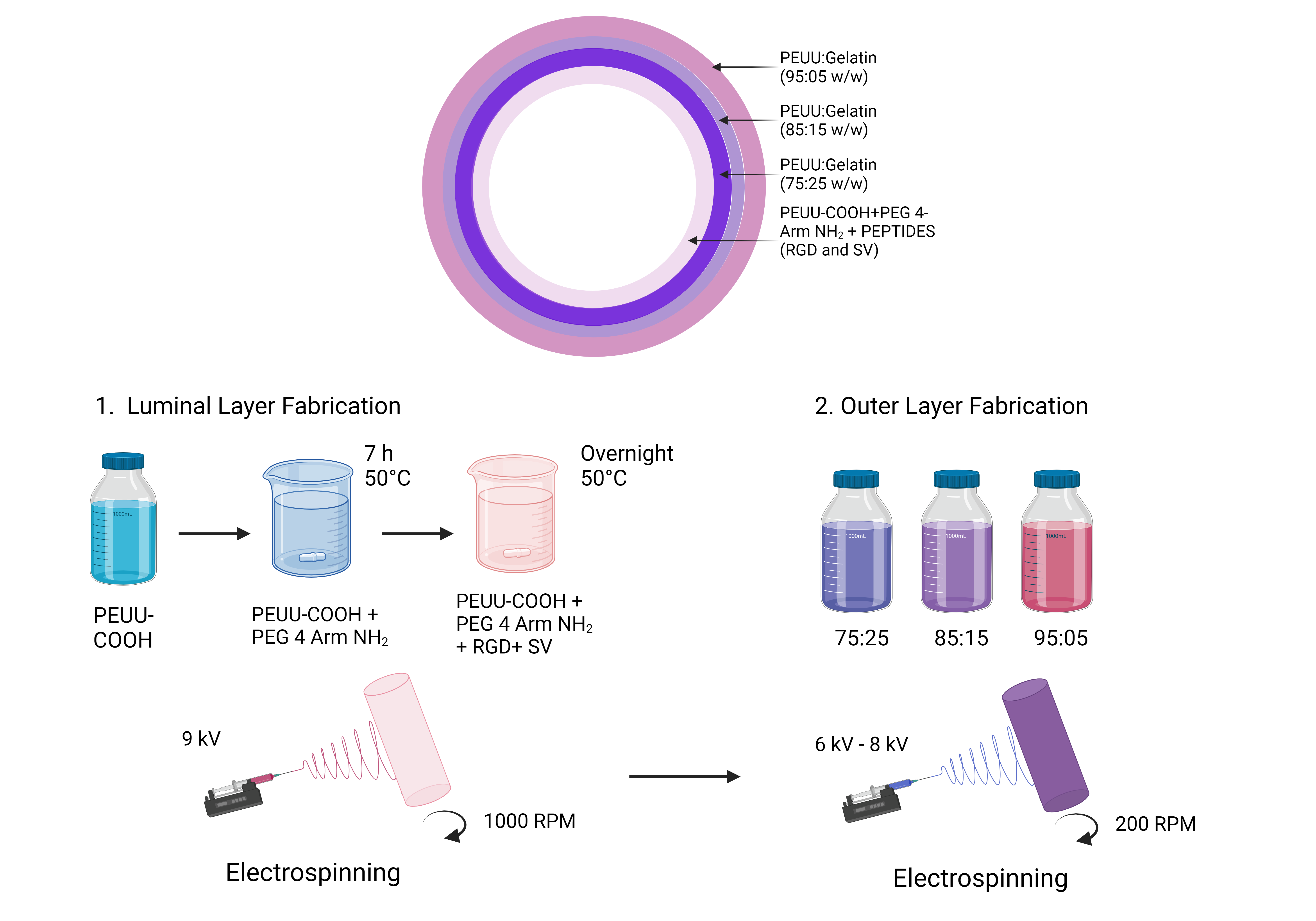


**Supplementary Figure 2** Schematic representation of the workflow for ML+P+P TEVG assessment.


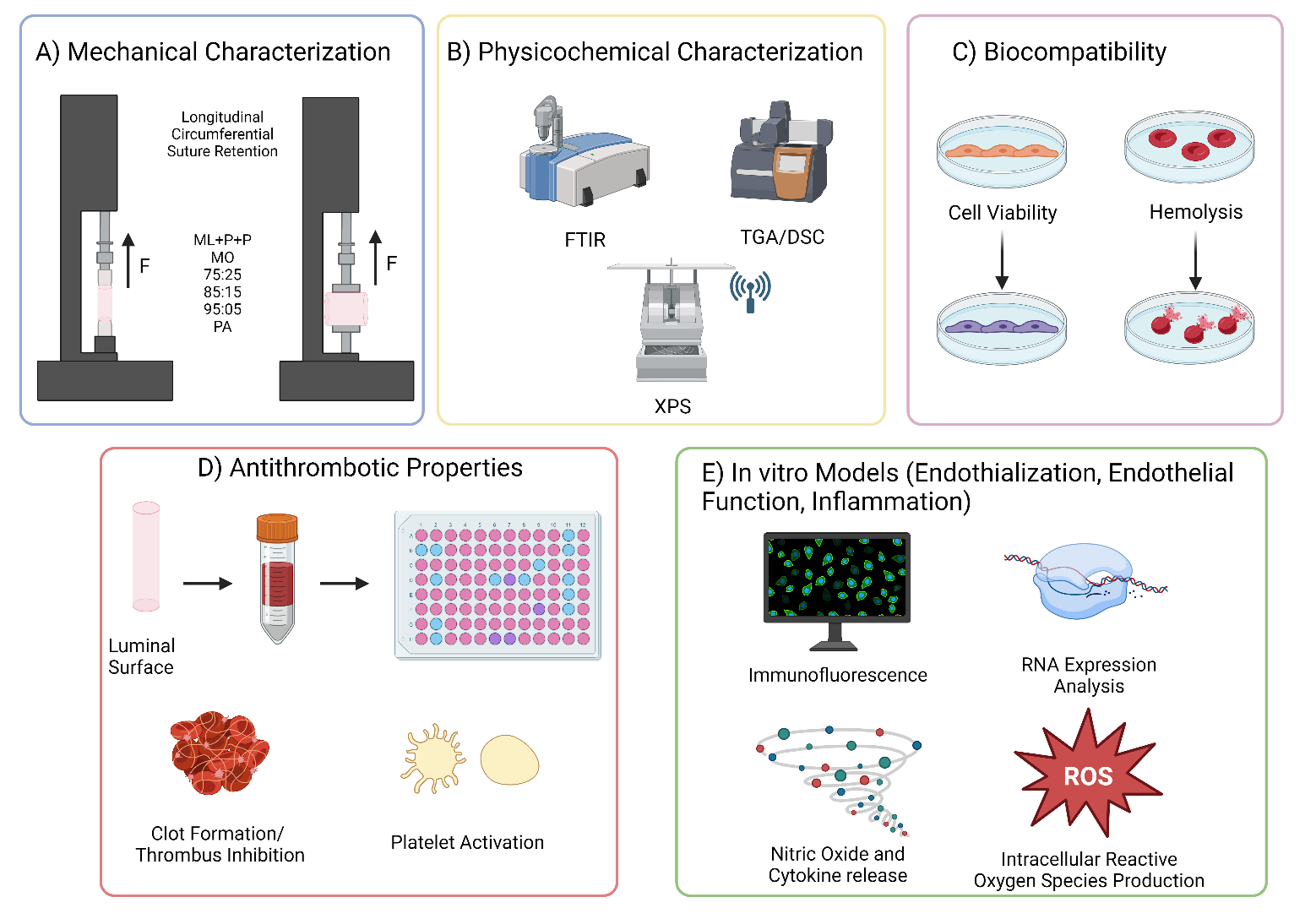
A) Mechanical properties, B) Physicochemical characterization, C) Biocompatibility, D) Antithrombotic Properties, E) in-vitro models (Endothelialization, endothelial function, inflammation).

# Supplementary Materials

For polymer synthesis, Polycaprolactone diol (PCL, Mn = 2000), dimethylolpropionic acid (DMPA), 1,4-Diisocyanatobutane (BDI), putrescine, and stannous octoate (Sn (Oct)2) were obtained from Sigma-Aldrich in St. Louis, MO, USA. Polymer solutions utilized 1,1,1,6,6,6-Hexafluoroisopropanol (HFIP) obtained from Oakwood Products, Inc. in Estill, USA. Polymer functionalization was performed with 4-arm Polyethylene Glycol with amine terminal groups Mw 20.000 (PEG 4 Arm NH_2_ - JKA7026) acquired from Sigma-Aldrich in St. Louis, MO, USA. Peptides SVVYLGR (SV) and GRGDNP (RGD) were synthesized by GenScript USA, Inc. Piscataway, USA. Amide bond formation was assisted by N-(3-Dimethylaminopropyl)-N′-ethyl carbodiimide hydrochloride (EDC - E7750), N-Hydroxysuccinimide (NHS - 130672) were purchased from Sigma-Aldrich in St. Louis, MO, USA. Vascular wall fabrication employed PEUU blended with Gelatin Type B 9000-70-8 and crosslinked with Glutaraldehyde solution 340855 purchased from Sigma-Aldrich in St. Louis, MO, USA.

For cell expansion and assays Dulbecco’s modified Eagle’s medium (DMEM), Roswell Park Memorial Institute 1640 Medium (RPMI1640), Endothelial Cell Growth Medium-2 BulletKit (EGM-2) were obtained from Lonza in Basel, Lonza-Hochhaus, Switzerland. Fetal Bovine Serum (FBS) was purchased from Biowest in Riverside, CA, USA. Human Umbilical Vein Endothelial Cells - HUVEC (CRL-1730), monocytes -THP-1 cells (ATCC TIB-202), and NCTC clone 929 – L929 cells were acquired from ATCC® in Manassas, VA, USA.

For immunofluorescence assays Hoechst 33258 Staining Dye Solution (ab228550) and Phalloidin-iFluor 488 Reagent (ab176753) were used for cell immunofluorescence. Rabbit monoclonal to CCR7 (ab32527) and Mouse monoclonal to CD163 (ab156769) were purchased for M1 and M2 macrophages identification along with Goat Anti-Mouse IgG H&L (Alexa Fluor® 647) (ab150115) and Goat Anti-Rabbit IgG H&L (Alexa Fluor® 488) (ab150077) were purchased from Abcam, Cambridge, UK. Bovine Serum Albumin (BSA - 37525) and Normal goat serum (10000C) were purchased from Sigma Aldrich from St. Louis, MO, USA.

# Supplementary Methods

## Polymer Synthesis (PEUU-COOH + PEG 4 Arm NH_2_ + PEPTIDES)

PEUU-COOH was synthesized using a previously described method, wherein PCL served as the soft segment, DMPA, and Putrescine acted as chain extenders, and BDI was added as the hard segment (Rodríguez-Soto et al., 2023). Next, we prepared a 12.5% (w/v) solution of PEUU-COOH in HFIP under constant magnetic stirring at 30°C. The in-situ functionalization was carried out using EDC/NHS Chemistry. Considering the presence of COOH functional groups in PEUU-COOH (3.56x10^−4^ mol/g), we determined the EDC/NHS ratio as 1:4 (3.56x10^−3^ mol/g) and conducted an activation step at 37°C for 15 min. Subsequently, PEG 4 Arm NH_2_ was introduced at a ratio of 1:3 (1.78x10^−4^ mol/g), and the reaction proceeded for 7 h at 50°C. Finally, RGD (20 µM) and SVV (20 µM) were added in a 1:3 ratio (1.78x10^−4^ mol/g), and the reaction continued overnight at 50°C (Rodríguez-Soto et al., 2023).

## Morphology

For analysis of the water uptake and stability on aqueous media, Tubular samples of the ML+P+P TEVG (10 mm in length) were freeze-dried overnight and weighed before immersing in PBS at 37°C for 24 h, according to the ASTM D570 – 98 international standards. To swelling ratio (%), determined by the formula: $\left[ \left( \boldsymbol{Wet weight-Dry weight} \right)\boldsymbol{/Dry weight} \right]\boldsymbol{*100}$, was calculated post-sample removal from the media, gentle shaking, and surface water removal with filter paper (Chung et al., 2015).

The porosity evaluation involved weighing tubular samples of the ML+P+P TEVG and immersing them in a known ethanol (non-solvent) volume (V1). The total ethanol volume and sample total volume after soaking were considered as V2. Post-soaking, samples were removed, and the remaining ethanol volume was recorded as V3. Porosity, expressed as a percentage, was calculated using the formula: $\left[ \left( \boldsymbol{V}\boldsymbol{1-V}\boldsymbol{2} \right)\boldsymbol{/}\left( \boldsymbol{V}\boldsymbol{2-V}\boldsymbol{3} \right) \right]\boldsymbol{*100}$ (McKenna et al., 2012).

## Mechanical properties

Longitudinal tensile strength, circumferential tensile strength, and suture retention tests were performed according to ISO 7198:2016(E). For the circumferential tensile strength test, the length (L) of the sample was never less than the nominal relaxed internal diameter as specified by the ISO 7198:2016(E) standard. The maximum load is documented in kiloNewtons (kN), while the length is expressed in millimeters (mm) as the ratio between Tmax and 2L. The circumferential tensile strength was then normalized by the initial transversal area, and the elongation was normalized by the initial length. Both longitudinal and circumferential tensile stress and strain were calculated and reported. For both ML+P+P TEVG and PA, Young’s Modulus was determined by analyzing the longitudinal stress-strain curve and calculated as the ratio between stress and strain.

A suture retention oblique procedure test was conducted to assess manipulability, following the ISO 7198:2016(E) guidelines. Samples were prepared by making perpendicular cuts to their long axis. Suturing was performed at a 2 mm distance from both the heel and the toe on the 45° cut end of the samples using 5-0 prolene sutures. The sutures were initially fixed to a specialized jaw for mechanical testing of biological samples on a biaxial Bose Electroforce Planar Biaxial Testbench (Eden Prairie, Minnesota, USA). The jaws were then secured by the jaws of the INSTRON Uniaxial Tensile machine, operating at a constant extension rate of 50 mm/min. During this procedure, data regarding the extension and load curves of the samples were recorded and subsequently processed to generate stress-strain curves.

## Physicochemical Characterization

### Chemical Surface analysis of the luminal layer

For the chemical surface characterization with X-ray photoelectron spectroscopy (XPS) technique, the samples were mounted on a non-conductive tape and a tantalum mesh with a nominal aperture of 430µm was used as a reference (Ta4f_7/2_ peak) for energy calibration. The surface charge compensation was achieved by an electron flood gun operated at 3 eV (20µA). The spot diameter, energy pass, and scan number were fixed at 200nm, 20eV, and 20, respectively. The fitting procedure was done by the XPSPeak4.1 software using a Shirley-type background, a 30% G-L peak shape, and full-width at half-maximum (FWHM) values selected from the database.

For by Fourier Transform Infrared (FTIR) analysis the absorbance data was processed by using the Baseline function from OriginPro 2023b Software. Then, the processed absorbance data was transformed into transmittance by obtaining the antilogarithm function of two minus the absorbance. Transmittance data was smoothed in OriginPro 2023b Software by the Savitzky/Golay method with 100 points of window and a Polynomial Order 4.

### Modeling of the ML+P+P TEVG degradation

Within the model, five distinct chemical species were considered, namely: Graft, macrophages, water, lipases, and ROS. The mass balance equation governing the transport of these solutes within the medium is expressed as Equation (1).

$\frac{\boldsymbol{\partial}\left( \boldsymbol{\varepsilon}_{\boldsymbol{p}}\boldsymbol{C}_{\boldsymbol{i}} \right)}{\boldsymbol{\partial t}}\boldsymbol{+ \nabla\cdot}\boldsymbol{J}_{\boldsymbol{i}}\boldsymbol{=}\boldsymbol{R}_{\boldsymbol{ⅈ}}\boldsymbol{+}\boldsymbol{S}_{\boldsymbol{ⅈ}}$ (1)

Where $\boldsymbol{\varepsilon}_{\boldsymbol{p}}$ represents porosity, $\boldsymbol{C}_{\boldsymbol{i}}$ stands for the concentration of each species, $\boldsymbol{R}_{\boldsymbol{ⅈ}}$ denotes the reaction rate for each species, $\boldsymbol{S}_{\boldsymbol{ⅈ}}$ signifies the species source for each species, and $\boldsymbol{J}_{\boldsymbol{i}}$ represents the mass flux that is defined by Equation (2) (Bolanos-Barbosa et al., 2023; COMSOL, 2022).

$\boldsymbol{J}_{\boldsymbol{i}}\boldsymbol{= -}\boldsymbol{D}_{\boldsymbol{e,i}}\boldsymbol{\nabla}\boldsymbol{C}_{\boldsymbol{i}}$ (2)

Where $\boldsymbol{D}_{\boldsymbol{e,i}}$ is the effective diffusion coefficient, particularly under the assumption of a saturated pore medium, as described by Equation (3).

$\boldsymbol{D}_{\boldsymbol{e,i}}\boldsymbol{=}\frac{\boldsymbol{\varepsilon}_{\boldsymbol{P}}}{\boldsymbol{\tau}_{\boldsymbol{F,i}}}$ (3)

Where, $\boldsymbol{\tau}_{\boldsymbol{F,i}}$ is the tortuosity factor for each species. The degradation model was coupled through the reaction $\boldsymbol{R}_{\boldsymbol{ⅈ}}$ presented in Equation (1). Similar to previous models in the literature (Eftimie & Barelle, 2021), it was assumed that macrophages arriving at the wound site followed logistical compartmentalization, as depicted in Equation (4).

$\frac{\boldsymbol{ⅆM}}{\boldsymbol{ⅆt}}\boldsymbol{=}\boldsymbol{k}_{\boldsymbol{m}}\boldsymbol{M}\left( \boldsymbol{1-}\frac{\boldsymbol{M}}{\boldsymbol{L}} \right)$(4)

Where M are the macrophages,$\boldsymbol{k}_{\boldsymbol{m}}$ is the constant of proportionality, and L is the Carrying Capacity. The production of reactive oxygen species (ROS) by macrophages over time was modeled using Equation (5).

$\frac{\boldsymbol{ⅆR}}{\boldsymbol{ⅆt}}\boldsymbol{=}\boldsymbol{k}_{\boldsymbol{R}}\boldsymbol{M}\left( \boldsymbol{1-}\frac{\boldsymbol{R}}{\boldsymbol{\beta}_{\boldsymbol{R}}\boldsymbol{M}} \right)$ (5)

Where $\boldsymbol{R}$ represents the ROS concentration,$\boldsymbol{k}_{\boldsymbol{R}}$serves as the rate constant that defines the rate at which macrophages produce ROS, and $\boldsymbol{\beta}_{\boldsymbol{R}}$ functions as a scaling parameter that modulates the rate of ROS production concerning macrophage concentration. The production of the enzymes was modeled using Equation (6).

$\frac{\boldsymbol{ⅆE}}{\boldsymbol{ⅆt}}\boldsymbol{=}\boldsymbol{k}_{\boldsymbol{E}}\boldsymbol{M}\left( \boldsymbol{1-}\frac{\boldsymbol{E}}{\boldsymbol{\beta}_{\boldsymbol{E}}\boldsymbol{M}} \right)$ (6)

Where $\boldsymbol{E}$ represents the Enzyme concentration, $\boldsymbol{k}_{\boldsymbol{E}}$ serves as the rate constant that defines the rate at which macrophages produce Enzymes, and $\boldsymbol{\beta}_{\boldsymbol{E}}$ functions as a scaling parameter that modulates the rate of enzyme production concerning macrophage concentration. The degradation of the grafts was modeled by considering hydrolysis, enzymatic degradation by lipases, and degradation induced by reactive oxygen species (ROS) as outlined in Equation (7).

$\frac{\boldsymbol{dG}}{\boldsymbol{dt}}\boldsymbol{=-}\boldsymbol{\phi}_{\boldsymbol{E}}\boldsymbol{*E*G-}\boldsymbol{\phi}_{\boldsymbol{R}}\boldsymbol{*R*G-}\boldsymbol{\phi}_{\boldsymbol{H}_{\boldsymbol{2}}\boldsymbol{O}}\boldsymbol{*G*}\boldsymbol{H}_{\boldsymbol{2}}\boldsymbol{O}$ (7)

Where $\phi_{E}$ represents the forward rate constant associated with enzymatic degradation, $\phi_{R}$ pertains to the forward rate constant governing degradation by reactive oxygen species, and $\phi_{H_{2}O}$ corresponds to the forward rate constant associated with hydrolysis.

## Biocompatibility

### Sample Sterilization and preparation

Rectangular 1 cm^2^ samples were cut and sterilized with ethylene oxide at the sterilization center of Fundación Cardio Infantil following the NTC 4426-1 and NTC 4426–2 standards, before the subsequent biocompatibility and anti-thrombogenic tests.

### Hemolysis test

For samples involving the use of blood, informed consent was obtained (Ethical Committee at the Universidad de Los Andes, minute number 928-2018) to collect O+ human blood. For hemolysis tests, blood was etracted using tubes with Ethylenediaminetetraacetic acid (EDTA). To isolate the erythrocytes and remove plasma content, five washes were performed on the anticoagulated blood using a physiological solution of 0.9% NaCl, centrifuging at 324 g for 5 min removing the supernatant each time. Erythrocytes were then resuspended in PBS to create a stock solution of 4x10^6^ erythrocytes/μL.

### Cell Expansion

The Human Umbilical Vein Endothelial primary cell line (HUVECs) was thawed and cultured in Endothelial Cell Growth Medium 2, supplemented with the following components: fetal bovine serum (1% v/v), hydrocortisone, hFGF, VEGF, IGF, hEGF, ascorbic acid, heparin, and glutamine. This culture was maintained at 37°C in a 5% CO2 atmosphere using T75 flasks, each containing a total volume of 7 mL. Half of the medium was replaced daily until the cells reached confluence. The cells were subcultured at a density of 4x10^3^ cells/cm^2^ until the 7th passage, at which point they were used for experiments.

For subculturing, the cell culture medium was aspirated, and the cells were washed twice with fresh PBS. Subsequently, 4 mL of a 10% v/v trypsin/EDTA solution was added, and the cells were incubated for 3 minutes to facilitate detachment. The cells were completely detached by gently resuspending the solution along the flask walls. To neutralize the trypsin, 5 mL of a trypsin-neutralizing solution was added, and the detached cells were transferred to centrifuge tubes. After centrifugation at 250 g for 5 min, the supernatant was discarded, and the cells were resuspended in fresh EGM-2 medium. They were then seeded into a new T75 culture flask at a density of 2.5x10^3^ cells/cm^2^.

The mouse fibroblastic cell line L929 was thawed and cultivated in Dulbecco's Modified Eagle Medium (DMEM) supplemented with fetal bovine serum (10% v/v). This culture was maintained in standard T25 flasks with a total culture volume of 5 ml. Daily medium replacements were performed until the cells reached confluence. Subsequently, the cells were subcultured at a density of 1.5x10^5^ cells/cm^2^ before their use in experiments. The subculturing process followed the previously described protocol, utilizing fresh DMEM medium, and the cells were then seeded into a new T25 culture flask at a density of 1.5x10^4^ cells/cm^2^.

The human monocytic cell line (THP-1) was thawed and cultivated in Roswell Park Memorial Institute (RPMI) 1640 medium, which was supplemented with fetal bovine serum (10% v/v) and β-Mercaptoethanol (0.05 mM). This culture was maintained in standard T75 flasks, with a total culture volume of 10 mL. Cell passages were conducted every other day by initially transferring the cell culture, along with the cells, to centrifuge tubes. Centrifugation was carried out at 250 g for 5 minutes, after which the culture medium was discarded. Subsequently, the cells were resuspended in a fresh RPMI medium to achieve a cell density of 1x10^5^ cells/mL. This culture was maintained until the 15th passage when the cells were used for further experimentation.

### Protein adsorption assay

For the assessment of protein adsorption capacity, 0.5 cm^2^ sterile samples were immersed in a 10% Fetal Bovine Serum (FBS) solution and incubated for 12 hours. Subsequently, these samples were transferred to a 96-well plate, washed with 50 μL of a 1% Sodium Dodecyl Sulfate (SDS) solution for 35 minutes at 37°C with gentle agitation at 100 rpm. The protein concentration in the supernatant was quantified using a Bicinchoninic Acid (BCA) Assay Kit (Quanti-Pro M3685, Sigma Aldrich, St. Louis, MO, USA). Both positive and negative controls were prepared with and without FBS, respectively. The BCA working solution was incubated for 30 minutes, and absorbance was measured at 565 nm. Protein concentration was determined using a linear regression model based on a Bovine Serum Albumin (BSA) standard curve with a known protein concentration of 2 mg/mL, and the protein concentration was subsequently normalized relative to the surface area.

## Preparation for cell seeding

### Protein adsorption protocol for cell seeding

1 cm² sterilized samples were placed in culture wells, and a cell media treatment was performed. Each well contained cell-specific media, the samples were incubated for 48 hours, with media changes every 24 hours to allow protein adsorption. After incubation, the media was removed, and the samples were left to air-dry overnight before cell seeding.

### Sample Autofluorescence Quenching for immunofluorescence stains

In our experiments, both the MO and ML+P+P TEVG samples exhibited autofluorescence. Prior to sterilization and cell seeding, we implemented an autofluorescence quenching protocol for the samples designated for immunofluorescence analysis. Initially, we prepared a photobleaching solution following a previously documented procedure (Sun et al., 2017). To elaborate, we first prepared a Tris Buffered saline solution (TBS 1X) with 150 mM NaCl and 50 mM Tris-Cl, adjusting the pH to 7.5. Additionally, we introduced 50 mM H2O2 to enhance the autofluorescence quenching process. The samples were then positioned in translucent glass flasks, each containing 50 mL of the photobleaching solution. To facilitate photobleaching, we lined the inside of a box with aluminum foil and a flat LED lamp with a power rating of 60W and an output of 4800 lumens was put inside. The samples were carefully placed above the lamp on a rocker platform set to oscillate at 10 rpm, ensuring constant movement. The set-up was maintained at 4°C and samples were exposed to direct light for 12 hours. Following this exposure, the samples underwent three wash cycles with Type II water and were allowed to air-dry prior to blocking.

### Sample blocking for immunofluorescence stains

During our experiments, we observed increased nonspecific binding of fluorophores in the MO and ML+P+P samples. Consequently, to address this issue, we conducted a blocking procedure utilizing EDC/NHS chemistry after quenching autofluorescence.

To prepare the blocking peptide solution, we started by digesting fetal bovine serum (FBS) in PBS (20% v/v). In brief, a 0.25% v/v trypsin solution was created in DPBS and mixed with the FBS solution at a 1:20 ratio. The resulting FBS-Trypsin mixture was incubated at 37°C for 2 hours. Afterward, centrifugation at 250g for 5 minutes was conducted to separate the supernatant containing the peptides, which we used further for blocking.

The autofluorescence-quenched samples were then gently placed with 1.5 mL of the blocking peptide solution. We added EDC to a final concentration of 2 mM and then NHS to a final concentration of 5 mM. These samples were placed on a rocker platform set to oscillate at 10 rpm, maintaining a temperature of 37°C to activate and conjugate any available free functional groups. Subsequently, the samples underwent washing with Type II water and were allowed to air-dry before sterilization.

### Porcine Artery Decellularization

As autologous grafts are considered the gold standard for successful vascular graft, we hypothesized that the use of a decellularized porcine carotid artery could represent an excellent scaffold reference for the analysis of ideal immunomodulatory responses.

To this end, we obtained porcine carotid arteries from the local slaughterhouse and proceeded with a decellularization protocol. The arteries were initially rinsed with tap water to remove any remaining blood and manually stripped of excess fat. Subsequently, the arteries were immersed in Type I water agitated at 200 rpm, and replaced five times until it ran clear. Following this, the arteries were soaked in a hypotonic 0.4% NaCl solution for 12 hours under agitation at 200 rpm. Afterward, the samples were kept cold and immersed in PBS 1X for ultrasonication for 15 minutes.

The arteries were then set up on a perfusion system equipped with a 5.2W pump, delivering a flow rate of 370 L/H through a tube with a 13 mm diameter connected to the artery. Perfusion was conducted using a solution consisting of SDS (1% v/v) and EDTA (0.1% v/v) for 2 hours, followed by a wash with Type I water for 30 minutes. Subsequently, another 15-minute ultrasonication step was performed. This SDS/washing perfusion and ultrasonication process was repeated once more. Following this, a solution of Triton X-100 (1% v/v) was perfused for an additional hour and washed twice with Type I water, as previously described. The artery was then removed from the mounted system and rinsed in Type II water until foam was no longer visible. The final decellularized artery was placed in PBS, which was changed three times before use. DNA quantification was conducted as detailed in the main document, and appropriately decellularized tissue was considered acceptable when the DNA content was below 50 ng/mg.

## Biomolecular analysis

### Reactive Oxygen Species Assay

Briefly, cell media was removed from TEVG and samples, replaced with DHE working solution, and incubated with live cells for 40 minutes at 37°C with 5% CO_2_. Media was removed, and samples were fixed with 4% formaldehyde for 10 min. Samples were rinsed with PBS, and a Hoechst solution 1:2000 was then added, followed by 10-minute incubation at 37°C. Samples were washed with PBS, and fluorescence images at 20X magnification were collected, maintaining the same light intensity and a 1-second exposure for all samples on Zen 3.8 software. Mean Fluorescence intensity was measured by subtracting the mean pixel intensity of the background from that of the cells (Shihan et al., 2021).

### Trizol RNA/DNA phase separation

Samples in Trizol were homogenized using a vortex for 5 minutes and then subjected to repeated passes through a 27-gauge syringe. After homogenization, chloroform was added, the mixture was resuspended, incubated for 3 minutes, and centrifuged at 12000 g for 15 minutes at 4°C. Once phase separation occurred, the aqueous phase containing the RNA was carefully removed for further RNA processing, while the remaining phases underwent DNA extraction.

### DNA Extraction and Quantification

The phases containing the DNA were subjected to DNA extraction. To this end, absolute ethanol was added to the remaining phases, mixed, and incubated for 3 minutes at room temperature. Subsequently, centrifugation was carried out at 2000 g for 5 minutes at 4°C to pellet the DNA. The phenol-ethanol supernatant was then discarded, and the pellet was resuspended in 0.1 M sodium citrate, followed by a 30-minute incubation. The DNA was washed through centrifugation at 2000 g for 5 minutes at 4°C, repeating this step three times. Afterward, the pellet was resuspended in 75% ethanol, followed by centrifugation at 2000 g at 4°C, discarding the supernatant after each centrifugation. The DNA was allowed to air dry for 5 minutes and was then resuspended in 8 mM NaOH. DNA concentration was confirmed by measuring the absorbance ratio at 260/280 nm using a DS-11 spectrophotometer (DeNovix, Wilmington, DE, USA).

### Agarose Gel for determination of RNA integrity

Before conducting RNA expression analysis, a 2% w/v agarose gel was prepared in TAE 1X (Tris-Acetate-EDTA) buffer. The agarose was melted in a microwave and then cooled to approximately 60°C before adding ethidium bromide (0.5 μg/mL). The molten agarose gel was carefully poured into a gel casting tray equipped with well-forming combs, and it was allowed to solidify for 30 minutes. RNA samples were subsequently mixed with RNA loading dye at a 1:5 ratio, with a minimum concentration requirement of at least 50 ng. These prepared samples were loaded onto the agarose gel alongside a known-sized RNA ladder. Electrophoresis was conducted at 90V for 1 hour in the absence of light. Following electrophoresis, the separated RNA bands were visualized under UV light (GELDOC XR+, BIORAD). Non-degraded samples were identified by the presence of two well-defined RNA bands corresponding to 28S and 18S rRNA.

### Nitric Oxide and Cytokine Release Assay

NO was used as a determinant of the endothelial activity and inflammatory activity. To this end the sample supernatant underwent deproteinization using a mixture of ZnSO4 and NaOH, followed by centrifugation at 2250 g for 10 minutes to eliminate the protein pellet. The deproteinized supernatant was then employed to reduce nitrates to nitrite using the Griess method, which involved incubating the sample with reagents A and B for 1 hour at 37°C. Following incubation, a brief centrifugation at 2250 g for 30 seconds was conducted, and the resultant supernatants were transferred to a fresh plate for absorbance measurement at 540 nm. NO concentration in cell media was determined using a linear regression model based on an NO standard curve with a known concentration of 100 µM.

VEGF, indicative of endothelialization and microvascularization promotion, was quantified using a VEGF immunoassay. To this end, the cell culture supernatant sample was placed onto the VEGF Elisa column, along with RD1W reagent, and incubated for 2 hours at room temperature. Subsequently, column contents were discarded, and a triple wash procedure was carried out. The VEGF conjugate was added, followed by another 2-hour incubation at room temperature and the triple washing procedure. The substrate solution was added to the column, and incubated for 25 minutes in darkness, after which the STOP solution was introduced and thoroughly mixed. Finally, absorbance was measured at 450 nm. VEGF concentration in cell media was determined using a linear regression model based on a VEGF standard with a known concentration of 1x10^-3^ µg.

M1/M2 macrophage balance is indicative of the inflammatory-regenerative signals occurring due to cell stimulation. Inflammatory related factors can be therefore identified through a Human M1/M2 Macrophage Panel. To this end individual capture beads were sonicated for 1 min and vortexed for 30 sec. A bead mixture reaction composed of each cytokine bead in the assay buffer along with the cell media was incubated for 2 hours at room temperature at 800 rpm, followed by a centrifugation wash at 250 g for 5 min. Immediately thereafter, samples were incubated for 1 h along with the Human Macrophage/Microglia Panel Detection Antibodies. Streptavidin-phycoerythrin (SA-PE) was added to the mixture, and incubation proceeded for another 30 min. After centrifugation wash at 250 g for 5 min, beads were resuspended in 150 µL of wash buffer. Data were acquired using a FACS CANTO II (BD, New Jersey, USA) flow cytometer, with a total of 4000 events used for each sample. Analysis was performed using LEGENDplex™ Data Analysis Software v8.0 (BioLegend, San Diego, CA, USA). Cytokine concentration in cell media was determined using a linear regression model based on the standard curves for each cytokine automatically determined by the software.

## Supplementary Tables

**Supplementary Table 1.** Parameters for the ML+P+P TEVG degradation simulation.

| **Parameter** | **Value** |
| --- | --- |
| Porosity of the medium (100:0) | 4.62% |
| Porosity of the medium (75:25) | 22.66% |
| Porosity of the medium (85:15) | 33.99% |
| Porosity of the medium (95:5) | 31.74% |
| Porosity of the medium (Media) | 52.1% |
| Porosity of the medium (Advecticia) | 58.31% |
| T1 TEVGs [µm] | 375.3 |
| T2 TEVGs [µm] | 153.146 |
| T3 TEVGs [µm] | 94.270 |
| T4 TEVGs [µm] | 382.906 |
| T1 Artery [µm] | 33.995 |
| T2 Artery [µm] | 506.827 |
| T3 Artery [µm] | 17.9 |
| Initial concentration of macrophages | 1 |
| Initial concentration of lipases | 0 |
| Initial concentration of ROS | 0 |
| Macrophages carrying capacity | 6.72 (Eftimie & Barelle, 2021) |
| Macrophages constant of proportionality | 0.23 (Eftimie & Barelle, 2021) |

**Supplementary Table 2.** Genes and primers used for the analysis of RNA expression in cell cultures.

|  | **Symbol** | **Name** | **Function** | **Primer sequence (5'-3')** | **Accession number** |
| --- | --- | --- | --- | --- | --- |
| Endothelial | VGFA | Vascular endothelial growth factor A | Induces proliferation and migration of vascular endothelial cells. Essential in angiogénesis (National Library of Medicine, 2023l). | F: TTGCCTTGCTGCTCTACCTCCA (OriGene Technologies Inc., 2023e).  R: GATGGCAGTAGCTGCGCTGATA (OriGene Technologies Inc., 2023e). | [NM_001025366](https://www.ncbi.nlm.nih.gov/nuccore/NM_001025366) |
|  | VEGFR | Epidermal growth factor receptor | Receptor for VEGF-induced endothelial proliferation, survival and migration (National Library of Medicine, 2023q, 2023t). | F: CCTGCAAGATTCAGGCACCTATG (OriGene Technologies Inc., 2023d).  R: GTTTCGCAGGAGGTATGGTGCT (OriGene Technologies Inc., 2023d). | [NC_000004.12](https://www.ncbi.nlm.nih.gov/gene/3791) |
|  | EDN1 | Endothelin 1 | Vasoconstrictor. Aberrant expression of this gene may promote tumorigenesis (National Library of Medicine, 2023p). | F: CTACTTCTGCCACCTGGACATC (OriGene Technologies Inc., 2023b).  R: TCACGGTCTGTTGCCTTTGTGG (OriGene Technologies Inc., 2023b). | [NC_000006.12](https://www.ncbi.nlm.nih.gov/gene/?term=1906) |
|  | VWF | Von Willebrand factor | Coding of a glycoprotein involved in hemostasis (National Library of Medicine, 2023m). | F: GTGGGAGATGTTTGCCTACG  R: GTTCATCAAAGGGTGGGCAG | [NC_000012.12](https://www.ncbi.nlm.nih.gov/gene/7450) |
|  | NOS3 | Nitric oxide synthase 3 | Mediator of neurotransmission, antimicrobial and antitumoral activities (National Library of Medicine, 2023k). | F: GCATCACCAGGAAGAAGACCTT  R: TGTGGCCTTCACTCTCTTTGC | [NC_000007.14](https://www.ncbi.nlm.nih.gov/gene/4846) |
|  | CD44 | Cluster of differentiation 44 | Cell-surface glycoprotein involved in cell-cell interactions, cell adhesion and migration (National Library of Medicine, 2023i). | F: CATCCTCACCTCCAACACCT  R: GTTGCTGGGATTGATGTCCT | [NC_000011.10](https://www.ncbi.nlm.nih.gov/gene/960) |
|  | PAR1 | Pseudoautosomal region 1 | Required for pairing of the X and Y chromosomes during male meiosis (Helena Mangs & Morris, 2007). | F: CGAGCTGCGGACTCGTTAGT  R: CAGCGGATGATGGACACGTA | [NC_000005.10](https://www.ncbi.nlm.nih.gov/gene/2149) |
|  | VCAM1 | Vascular cell adhesion molecule1 | Mediation of leukocyte-endothelial cell adhesion and signal transduction (National Library of Medicine, 2023h). | F: GATTCTGTGCCCACAGTAAGGC (OriGene Technologies Inc., 2023c).  R: TGGTCACAGAGCCACCTTCTTG (OriGene Technologies Inc., 2023c). | [NC_000001.11](https://www.ncbi.nlm.nih.gov/gene/7412) |
|  | COX2 | Cytochrome c oxidase subunit II | Cytochrome-c oxidase activity (National Library of Medicine, 2023g). | F: CGGTGAAACTCTGGCTAGACAG (OriGene Technologies Inc., 2023a).  R: GCAAACCGTAGATGCTCAGGGA (OriGene Technologies Inc., 2023a). | [NC_012920.1](https://www.ncbi.nlm.nih.gov/gene/4513) |
| Macrophages | CD80 | CD80 molecule | Induces T-cell proliferation and cytokine production. Can act as a receptor for adenovirus subgroup B (National Library of Medicine, 2023a). | GCCAGTAGATGCGAGTTTGTGC | [NC_000003.12](https://www.ncbi.nlm.nih.gov/gene?Db=gene&Cmd=DetailsSearch&Term=941) |
|  | NOS2 | Nitric oxide synthase 2 | Mediator of neurotransmission, antimicrobial and antitumoral activities. Expressed in the liver (National Library of Medicine, 2023j). | F: GGTCCATGATGGTACCATTCTGC  R: CATCCCAAATACGAGTGGTTTCG | [NC_000017.11](https://www.ncbi.nlm.nih.gov/gene/4843) |
|  | TNFA | Tumor necrosis factor alpha | Encodes a multifunctional proinflammatory cytokine that belongs to the tumor necrosis factor superfamily (National Library of Medicine, 2023u). | F: GTCTTCCTCTCTCACGCACC  R: TGGGCTAGAGGCTTGTCACT | [NC_000006.12](https://www.ncbi.nlm.nih.gov/gene/7124) |
|  | IL12 | Interleukin 12 | Enhances the stability, intracellular trafficking, and export of the p35 subunit (Jalah et al., 2013). | F: AAGGCCAGACAAACTCTAGAATTC  R: TTGGTTAACTCCAGTGGTAAACAGG | [NC_000005.10](https://www.ncbi.nlm.nih.gov/gene/3593) |
|  | CD163 | Cluster of differentiation 163 | Expressed in monocytes and macrophages, involved in the clearance and endocytosis of hemoglobin/haptoglobin complex (National Library of Medicine, 2023c). | F: CGGCTGCCTCCACCTCTAAGT  R: ATGAAGATGCTGGCGTGACA | [NC_000012.12](https://www.ncbi.nlm.nih.gov/gene/9332) |
|  | CD206 | Mannose receptor c, Type 1 | Medicates the endocytosis of glycoproteins by macrophages (National Library of Medicine, 2023f). | CAAAACGCTCGCGCATTGTCCA | [NC_000010.11](https://www.ncbi.nlm.nih.gov/gene/4360) |
|  | ARG1 | Arginase 1 | Catalyzes the hydrolysis of arginine to ornithine and urea. Expressed predominantly in the liver as a component of the urea cycle (National Library of Medicine, 2023o). | F: GGATCATTGGAGCCCCTTTCTC  R: TCAAGCAGACCAGCCTTTCTC | [NC_000006.12](https://www.ncbi.nlm.nih.gov/gene/383) |
|  | IL10 | Interleukin 10 | Chemoattractant for activated T cells into sites of tissue inflammation (Dufour et al., 2002). | F: CAAGCCTTGTCGGAGATGAT  R: TTTTCACAGGGGAGAAATCG | [NC_000001.11](https://www.ncbi.nlm.nih.gov/gene/3586) |
| Coculture | CD68 | Cluster of differentiation 68 | Ligand of antigen CD28. Codifies for the membrane protein type 1 (National Library of Medicine, 2023b). | ATGAGAGGCAGCAAGATGGACC | [NC_000017.11](https://www.ncbi.nlm.nih.gov/gene/968) |

**Supplementary Table 3.** Cytokines that were used for the analysis of the M1/M2 panel.

|  | **Symbol** | **Cytokine** | **Function** |
| --- | --- | --- | --- |
| M1 | VEGFA | Vascular endothelial growth factor A | Induces proliferation and migration of vascular endothelial cells. Essential in angiogenesis (National Library of Medicine, 2023l). |
|  | IL12P70 | Interleukin 12 | Enhances the stability, intracellular trafficking, and export of the p35 subunit (Jalah et al., 2013). |
|  | TNFA | Tumor necrosis factor-alpha | Encodes a multifunctional proinflammatory cytokine that belongs to the tumor necrosis factor superfamily (National Library of Medicine, 2023u). |
|  | IL1B | Interleukin 1 Beta | An important mediator of the inflammatory response and is involved in a variety of cellular activities, including cell proliferation, differentiation, and apoptosis (National Library of Medicine, 2023n). |
|  | IL12P40 | Interleukin 12 Subunit p40 | Important for sustaining a sufficient number of memory/effectors Th1 cells to mediate long-term protection against an intracellular pathogen (National Library of Medicine, 2023e). |
|  | IL23 | Interleukin 23 | Ability to potently enhance the expansion of T helper type 17 (Th17) cells and responsible for many of the inflammatory autoimmune responses (Tang et al., 2012). |
|  | IP10 | Interferon gamma-induced protein 10 | Stimulation of monocytes, natural killer and T-cell migration, and modulation of adhesion molecule expression (National Library of Medicine, 2023d). |
| M2 | IL10 | Interleukin 10 | Chemoattractant for activated T cells into sites of tissue inflammation (Dufour et al., 2002). |
|  | TARC | Thymus and activation-regulated chemokine | Ligand for CCR4, which is predominantly expressed on Th2 lymphocytes, basophils, and natural killer cells (Saeki & Tamaki, 2006). |
|  | IL1RA | Interleukin 1 Receptor Antagonist | Inhibits the activities of interleukin 1, alpha (IL1A) and interleukin 1, beta (IL1B), and modulates a variety of interleukin 1 related immune and inflammatory responses, particularly in the acute phase of infection and inflammation (National Library of Medicine, 2023r). |
| M1/M2 | IL6 | Interleukin 6 | Induces a transcriptional inflammatory response through the interleukin 6 receptor (National Library of Medicine, 2023s). |

# Supplementary Results

**Supplementary Table 4.** Summary of the binding energies (B.E.), full-width at half-maximum (FWHM), and area under the curve of XPS sub-peaks derived from the functionalized Lumina layer (ML+P and ML+P+P) samples. The sub-peaks are labeled in ascending order of binding energy. The resolution of the equipment used is approximately 0.5 eV. The intensity counts for the area under the curve are arbitrary and have been normalized to C1s (284.6 eV). The binding energies (B.E.) of the C1s, O1s, and N1s core-levels of the Luminal surface of the multilayered TEVG were obtained from the integration of high-resolution XPS spectra peaks shown in Fig. 4 A) and B) on the main manuscript.

| Functionalized sample | Main peak | Sub-peaks | | | |
| --- | --- | --- | --- | --- | --- |
|  |  | Label | B.E. (eV) | FHWM | Area (a.u.) |
| ML+P | C1s | -C-H  -C-C  -C-N  -C-O  N-C=O  O-C=O | 283.45  284.27  285.01  286.07  287.63  288.68 | 0.97  0.95  1.06  1.14  1.23  0.89 | 20.4  64.2  60.8  31.7  12.2  10.6 |
|  | O1s | -O=C  -O-C  -OH | 530.68  531.75  532.87 | 1.43  1.38  1.30 | 45.8  60.4  36.9 |
|  | N1s | -N-H  -N-H_2_  -N-C=O | 398.29  398.94  399.70 | 1.46  1.18  1.20 | 5.5  12.6  5.1 |
| ML+ P+P | C1s | -C-H  -C-C  -C-N  -C-O  N-C=O  O-C=O | 283.64  284.41  285.10  286.13  287.66  288.67 | 0.97  1.04  1.13  1.23  1.30  1.00 | 25.5  67.2  57.3  37.6  10.9  9.4 |
|  | O1s | -O=C  -O-C  -OH | 530.89  531.85  532.91 | 1.36  1.30  1.32 | 54.2  77.5  33.9 |
|  | N1s | -N-H  -N-H_2_  -N-C=O | 398.15  398.97  399.62 | 1.17  1.11  1.10 | 3.0  11.5  8.4 |

##
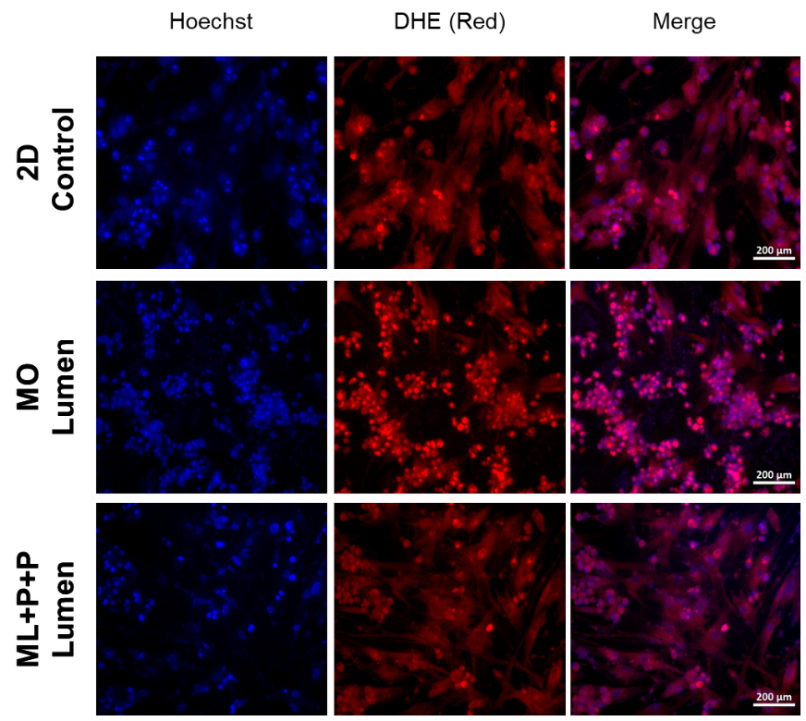


## Supplementary Figure 3. Intracellular ROS staining for Endothelial cells (HUVECs) seeded on the luminal surface of the ML+P+P TEVG.

##
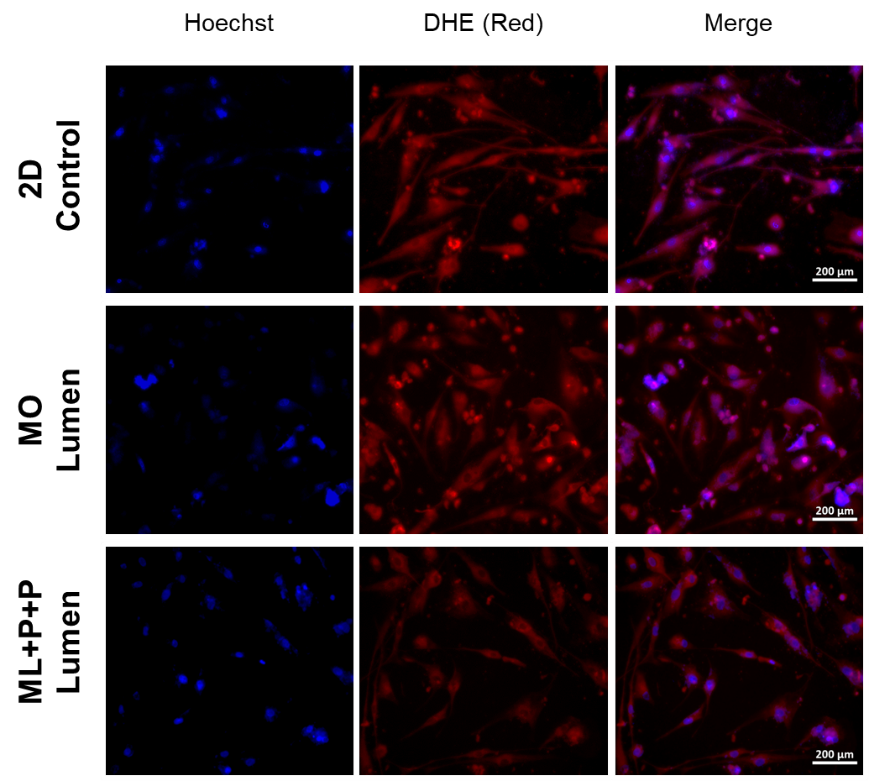


## Supplementary Figure 4. Intracellular ROS staining for the coculture of Endothelial cells (HUVECs) and macrophages (THP-1) seeded on the luminal surface of the ML+P+P TEVG.


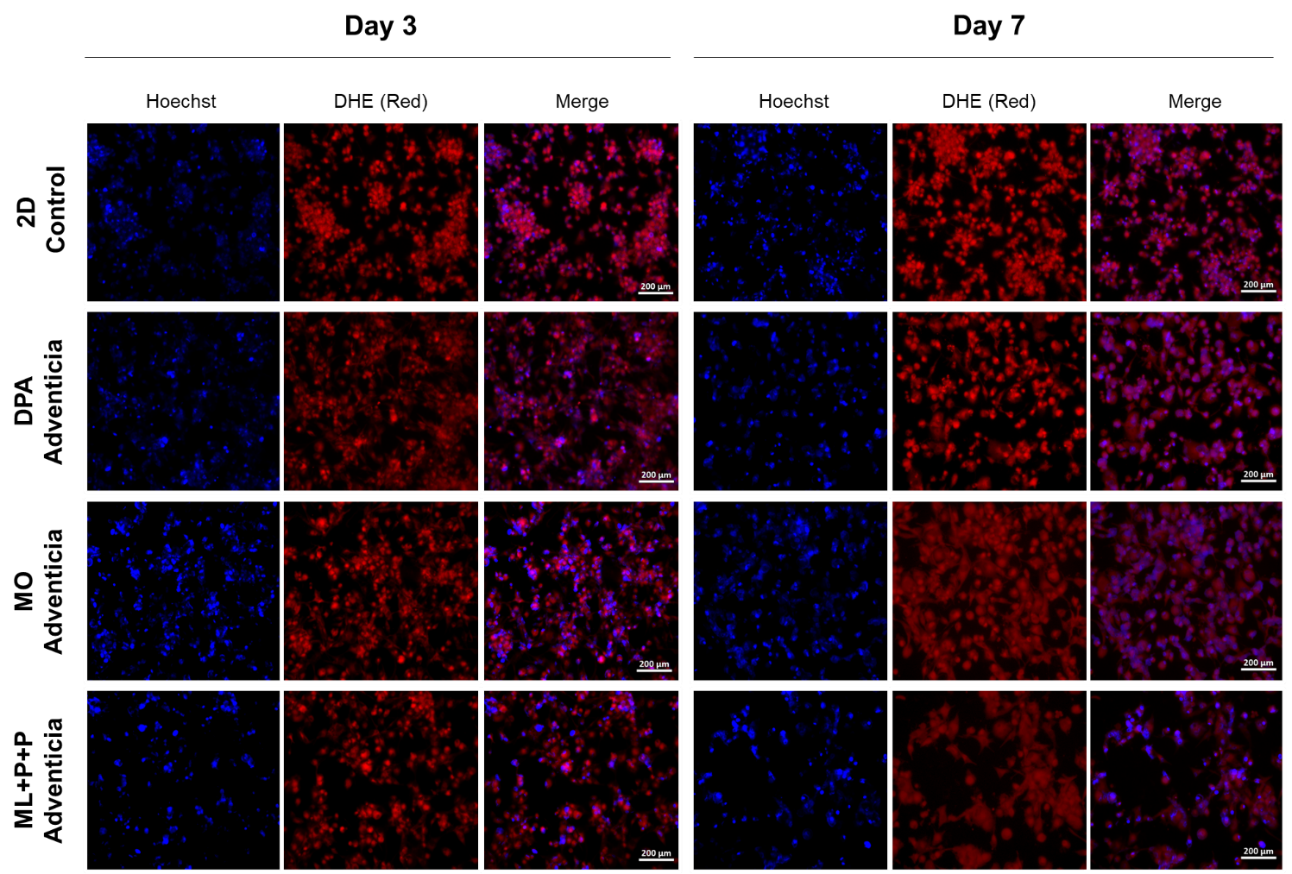


**Supplementary Figure 5.** Intracellular ROS staining for macrophages (THP-1) seeded on the adventitial surface of the ML+P+P TEVG.

**Supplementary Table 5.** RNA expression values obtained in the culture of Endothelial cells (HUVECs) seeded on the luminal surface of the ML+P+P TEVG (Log2 Fold Change, Mean ± SD).

|  | Control vs MO | Control vs ML+P+P | MO vs ML+P+P |
| --- | --- | --- | --- |
| VGFA | -9.2 ± 1.4 | 8.5 ± 1.0 | 17.8 ± 1.0 |
| VEGFR | -7.1 ± 1.2 | 9.7 ± 1.5 | 1.5 ± 1.1 |
| EDN1 | -5.7 ± 2.2 | 1.5 ± 1.1 | 7.2 ± 1.1 |
| VWF | -17.5 ± 2.0 | 5.4 ± 0.2 | 22.9 ± 0.2 |
| NOS3 | -7.0 ± 1.0 | 4.4 ± 0.8 | 11.4 ± 0.8 |
| CD44 | -3.6 ± 4.3 | 5.0 ± 0.7 | 8.5 ± 0.7 |
| PAR1 | -7.5 ± 0.7 | 4.1 ± 1.1 | 11.6 ± 1.1 |
| VCAM | -8.0 ± 1.6 | -0.1 ± 1.3 | -2.8 ± 0.6 |
| COX2 | -4.6 ± 0.3 | -2.8 ± 0.6 | 1.8 ± 0.6 |

**Supplementary Table 6.** RNA expression values and cytokine profiles obtained in the coculture of Endothelial cells (HUVECs) and macrophages (THP-1) seeded on the luminal surface of the ML+P+P TEVG (Log2 Fold Change, Mean ± SD).

1. Coculture RNA Expression from HUVECs and THPs at day 7

|  | Control vs MO | Control vs ML+P+P | MO vs ML+P+P |
| --- | --- | --- | --- |
| VGFA | 0.5 ± 0.6 | 2.6 ± 0.4 | 2.1 ± 0.4 |
| VEGFR | 3.9 ± 0.6 | 5.1 ± 0.4 | 1.2 ± 0.4 |
| NOS3 | 0.9 ± 0.2 | 1.5 ± 1.2 | 0.5 ± 1.2 |
| VCAM1 | 0.3 ± 0.4 | 0.0 ± 0.9 | -0.3 ± 0.9 |
| COX2 | -2.8 ± 0.7 | -3.0 ± 1.3 | -0.2 ± 1.3 |
| TNFA | 2.4 ± 1.5 | 1.0 ± 0.6 | -1.4 ± 0.6 |
| NOS2 | 4.8 ± 1.5 | 2.6 ± 1.2 | -2.2 ± 1.2 |
| ARG1 | -1.6 ± 0.5 | 4.0 ± 0.9 | 5.6 ± 0.9 |
| CD206 | -8.6 ± 0.4 | 1.5 ± 0.9 | 10.1 ± 0.9 |
| CD68 | 5.2 ± 0.8 | 5.1 ± 0.4 | 6.7 ± 0.4 |

1. Coculture Cytokine Release from HUVECs and THPs at day 7

|  | Control vs MO | Control vs ML+P+P | MO vs ML+P+P |
| --- | --- | --- | --- |
| IL-12p70 | 0.0 ± 0.3 | -0.3 ± 0.5 | 0.2 ± 0.4 |
| TNF-α | 0.2 ± 0.3 | -4.1 ± 5.2 | -3.5 ± 4.5 |
| IL-1β | 0.0 ± 2.7 | -0.5 ± 0.3 | -1.7 ± 0.2 |
| IL-12p40 | -0.9 ± 3.7 | 1.5 ± 2.8 | 0.6 ± 2.4 |
| IL-23 | 2.1 ± 3.1 | -0.1 ± 0.0 | -4.8 ± 2.4 |
| IP-10 | 0.4 ± 0.0 | -0.2 ± 1.1 | -0.6 ± 0.9 |
| IL-10 | -0.1 ± 0.8 | -0.2 ± 0.4 | 0.1 ± 0.3 |
| TARC | -1.3 ± 2.3 | 0.6 ± 0.8 | 1.8 ± 0.7 |
| IL-1RA | -2.1 ± 4.4 | -8.8 ± 1.1 | -7.2 ± 3.2 |
| IL-6 | -2.6 ± 5.8 | -6.3 ± 7.3 | -5.8 ± 6.3 |

**Supplementary Table 7.** RNA expression values and cytokine profiles obtained for macrophages (THP-1) seeded on the adventitial surface of the ML+P+P TEVG (Log2 Fold Change, Mean ± SD)

1. RNA Expression THPs at Day 3

|  | Control vs DPA | Control vs MO | Control vs ML+P+P | DPA vs ML+P+P | MO vs ML+P+P |
| --- | --- | --- | --- | --- | --- |
| CD80 | 1.8 ± 2.5 | -3.1 ± 0.6 | -2.0 ± 0.3 | -3.8 ± 0.3 | 1.1 ± 0.3 |
| NOS 2 | 4.4 ± 1.3 | -2.6 ± 1.2 | 2.2 ± 0.6 | -2.2 ± 0.6 | 4.8 ± 0.6 |
| TNFA | 10.3 ± 0.2 | 2.7 ± 0.7 | 0.0 ± 0.1 | -10.3 ± 0.1 | -2.6 ± 0.1 |
| IL12 | 6.3 ± 0.2 | -1.7 ± 1.9 | -1.4 ± 1.8 | -7.7 ± 1.8 | 0.3 ± 1.8 |
| CD163 | 1.7 ± 2.0 | -2.2 ± 0.6 | 3.5 ± 0.2 | 1.9 ± 0.2 | 5.7 ± 0.2 |
| CD206 | 13.2 ± 0.6 | 6.0 ± 1.3 | 2.8 ± 0.5 | -10.4 ± 0.5 | -3.2 ± 0.5 |
| ARG1 | 4.3 ± 2.0 | -1.8 ± 0.0 | -1.0 ± 0.9 | -4.3 ± 0.9 | 0.6 ± 0.9 |
| IL10 | 3.6 ± 0.9 | -3.4 ± 0.7 | -1.9 ± 1.0 | -5.5 ± 1.0 | 1.4 ± 1.0 |
| VEGFA | 16.4 ± 1.2 | 11.4 ± 1.4 | 10.7 ± 0.0 | 7.1 ± 0.0 | 14.0 ± 0.0 |

1. RNA Expression THPs at Day 7

|  | Control vs DPA | Control vs MO | Control vs ML+P+P | DPA vs ML+P+P | MO vs ML+P+P |
| --- | --- | --- | --- | --- | --- |
| CD80 | 3.0 ± 1.5 | 6.5 ± 1.7 | -7.4 ± 1.3 | -10.4 ± 1.3 | -13.9 ± 1.3 |
| NOS 2 | -7.5 ± 2.5 | 6.6 ± 3.9 | -5.6 ± 0.6 | 1.9 ± 0.6 | -12.2 ± 0.6 |
| TNFA | 2.4 ± 0.6 | 1.4 ± 0.5 | -2.2 ± 2.9 | -4.7 ± 2.9 | -3.6 ± 2.9 |
| IL12 | -1.9 ± 0.5 | 4.6 ± 0.7 | -1.2 ± 0.8 | 0.7 ± 0.8 | -5.7 ± 0.8 |
| CD163 | -7.2 ± 0.1 | -1.1 ± 0.5 | 0.2 ± 0.3 | 7.4 ± 0.3 | 1.3 ± 0.3 |
| CD206 | 0.4 ± 0.6 | 6.4 ± 0.5 | 8.6 ± 1.6 | 6.5 ± 1.6 | 0.5 ± 1.6 |
| ARG1 | -2.4 ± 0.2 | -5.0 ± 0.5 | 2.0 ± 0.1 | 4.4 ± 0.1 | 7.4 ± 0.1 |
| IL10 | -2.4 ± 0.4 | 2.2 ± 0.8 | 4.2 ± 0.6 | 6.6 ± 0.6 | 2.0 ± 0.6 |
| VEGFA | 10.3 ± 0.6 | 15.2 ± 0.5 | 14.0 ± 0.7 | 16.4 ± 0.7 | 11.8 ± 0.7 |

1. Cytokine Release from THPs at Day 3

|  | Control vs DPA | Control vs MO | Control vs ML+P+P | DPA vs ML+P+P | MO vs ML+P+P |
| --- | --- | --- | --- | --- | --- |
| IL-12p70 | 1.4 ± 1.8 | 1.8 ± 0.3 | 1.6 ± 1.6 | 0.0 ± 1.1 | 0.2 ± 1.1 |
| TNF-α | 1.1 ± 1.7 | 8.3 ± 0.1 | -5.7 ± 0.1 | -7.8 ± 3.9 | -13.4 ± 6.7 |
| IL-1β | 2.3 ± 3.6 | 7.5 ± 0.3 | -2.6 ± 0.8 | -8.3 ± 0.7 | -10.0 ± 0.7 |
| IL-12p40 | 0.5 ± 0.3 | 0.2 ± 0.0 | -3.9 ± 0.6 | -4.4 ± 2.0 | -7.0 ± 3.0 |
| IL-23 | 0.3 ± 0.2 | 3.7 ± 0.1 | -0.5 ± 1.9 | -0.8 ± 1.7 | -7.9 ± 4.8 |
| IP-10 | -0.1 ± 0.3 | 1.2 ± 0.5 | -8.0 ± 2.1 | -8.0 ± 1.8 | -8.6 ± 1.8 |
| IL-10 | -0.2 ± 0.3 | -5.7 ± 0.3 | -1.9 ± 0.1 | -1.8 ± 0.1 | 1.0 ± 0.1 |
| TARC | 0.0 ± 0.0 | -15.6 ± 0.5 | 0.0 ± 0.0 | 0.0 ± 0.0 | 12.6 ± 0.0 |
| IL-1RA | -0.2 ± 0.1 | -0.1 ± 0.1 | 0.7 ± 0.3 | 0.8 ± 0.3 | 0.9 ± 0.3 |
| IL-6 | -0.6 ± 0.1 | 5.9 ± 0.1 | -2.3 ± 0.1 | -1.7 ± 0.8 | -6.7 ± 2.9 |

1. Cytokine Release from THPs at Day 7

|  | Control vs DPA | Control vs MO | Control vs ML+P+P | DPA vs ML+P+P | MO vs ML+P+P |
| --- | --- | --- | --- | --- | --- |
| IL-12p70 | 0.4 ± 0.7 | 4.7 ± 0.6 | 0.0 ± 0.0 | 0.0 ± 0.0 | 0.0 ± 0.0 |
| TNF-α | 3.2 ± 0.1 | 0.8 ± 0.1 | 0.3 ± 0.2 | -3.0 ± 0.2 | -0.5 ± 0.2 |
| IL-1β | 3.0 ± 0.1 | 1.3 ± 0.1 | 0.2 ± 0.3 | -2.8 ± 0.3 | -1.1 ± 0.3 |
| IL-12p40 | 0.3 ± 0.7 | 0.4 ± 0.3 | 0.1 ± 0.5 | -0.2 ± 0.5 | -0.3 ± 0.5 |
| IL-23 | 0.2 ± 0.2 | 0.3 ± 0.1 | 0.0 ± 0.2 | -0.1 ± 0.2 | -0.3 ± 0.2 |
| IP-10 | -0.2 ± 0.0 | -0.2 ± 0.1 | -1.8 ± 0.1 | -1.6 ± 0.1 | -1.6 ± 0.1 |
| IL-10 | 3.7 ± 0.2 | 1.8 ± 0.3 | 1.3 ± 0.5 | -2.4 ± 0.5 | -0.4 ± 0.5 |
| TARC | -1.0 ± 2.3 | -0.8 ± 1.2 | 0.9 ± 0.4 | 1.1 ± 0.4 | 1.4 ± 0.4 |
| IL-1RA | 2.3 ± 0.3 | 1.3 ± 0.1 | 1.8 ± 0.2 | -0.5 ± 0.2 | 0.5 ± 0.2 |
| IL-6 | 2.5 ± 0.1 | 1.9 ± 0.2 | 0.5 ± 0.1 | -2.1 ± 0.1 | -1.4 ± 0.1 |

**Supplementary Table 8.** Total DNA extracted from the cell seeded samples with either HUVEC, THP-1 or coculture (HUVEC+THP-1) (µg/cm^2^ - Mean ± SD)

| **HUVEC Total DNA at 7 days (µg/cm2)** | | |  |
| --- | --- | --- | --- |
| Control | MO | ML+P+P |  |
| 0.6 | 0.9 | 0.6 |  |
| 0.8 | 1.0 | 0.6 |  |
| 1.0 | 0.7 | 0.6 |  |
| 0.9 | 0.9 | 0.6 |  |
| **0.8** | **0.9** | **0.6** | Mean |
| **0.1** | **0.1** | **0.0** | SD |

| **Coculture (HUVEC +THP-1) Total DNA at 7 days (µg/cm2)** | | |  |
| --- | --- | --- | --- |
| Control | MO | ML+P+P |  |
| 1.7 | 1.4 | 1.3 |  |
| 1.2 | 1.3 | 1.3 |  |
| 1.7 | 1.9 | 1.3 |  |
| 1.2 | 1.9 | 1.2 |  |
| **1.5** | **1.6** | **1.3** | Mean |
| **0.3** | **0.3** | **0.0** | SD |

| **THP-1 Total DNA at 3 days (µg/cm2)** | | | |  |
| --- | --- | --- | --- | --- |
| Control | DPA | MO | ML+P+P |  |
| 2.0 | 1.8 | 1.9 | 2.0 |  |
| 1.3 | 1.9 | 1.8 | 1.4 |  |
| 2.4 | 1.8 | 1.3 | 1.2 |  |
| 1.2 | 1.9 | 1.4 | 1.4 |  |
| **1.7** | **1.9** | **1.6** | **1.5** | Mean |
| **0.6** | **0.1** | **0.3** | **0.3** | SD |

| **THP-1 Total DNA at 3 days (µg/cm2)** | | | |  |
| --- | --- | --- | --- | --- |
| Control | DPA | MO | ML+P+P |  |
| 2.2 | 2.1 | 1.2 | 1.5 |  |
| 2.0 | 1.4 | 1.7 | 1.3 |  |
| 2.1 | 1.9 | 1.9 | 1.2 |  |
| 1.6 | 1.3 | 1.5 | 1.8 |  |
| **1.9** | **1.7** | **1.6** | **1.5** | Mean |
| **0.3** | **0.4** | **0.3** | **0.3** | SD |

**References**

Bolanos-Barbosa, A. D., Rodríguez, C. F., Acuña, O. L., Cruz, J. C., & Reyes, L. H. (2023). The Impact of Yeast Encapsulation in Wort Fermentation and Beer Flavor Profile. *Polymers*, *15*(7), 1742. https://doi.org/10.3390/polym15071742

Chung, E., Ju, H. W., Park, H. J., & Park, C. H. (2015). Three‐layered scaffolds for artificial esophagus using poly(ɛ‐caprolactone) nanofibers and silk fibroin: An experimental study in a rat model. *Journal of Biomedical Materials Research Part A*, *103*(6), 2057–2065. https://doi.org/10.1002/jbm.a.35347

COMSOL. (2022). *Chemical Reaction Engineering Module User’s Guide*. https://doc.comsol.com/6.1/doc/com.comsol.help.chem/ChemicalReactionEngineeringModuleUsersGuide.pdf

Dufour, J. H., Dziejman, M., Liu, M. T., Leung, J. H., Lane, T. E., & Luster, A. D. (2002). IFN-γ-Inducible Protein 10 (IP-10; CXCL10)-Deficient Mice Reveal a Role for IP-10 in Effector T Cell Generation and Trafficking. *The Journal of Immunology*, *168*(7), 3195–3204. https://doi.org/10.4049/jimmunol.168.7.3195

Eftimie, R., & Barelle, C. (2021). Mathematical investigation of innate immune responses to lung cancer: The role of macrophages with mixed phenotypes. *Journal of Theoretical Biology*, *524*, 110739. https://doi.org/10.1016/j.jtbi.2021.110739

Helena Mangs, A., & Morris, B. (2007). The Human Pseudoautosomal Region (PAR): Origin, Function and Future. *Current Genomics*, *8*(2), 129–136. https://doi.org/10.2174/138920207780368141

Jalah, R., Rosati, M., Ganneru, B., Pilkington, G. R., Valentin, A., Kulkarni, V., Bergamaschi, C., Chowdhury, B., Zhang, G.-M., Beach, R. K., Alicea, C., Broderick, K. E., Sardesai, N. Y., Pavlakis, G. N., & Felber, B. K. (2013). The p40 Subunit of Interleukin (IL)-12 Promotes Stabilization and Export of the p35 Subunit. *Journal of Biological Chemistry*, *288*(9), 6763–6776. https://doi.org/10.1074/jbc.M112.436675

McKenna, K. A., Hinds, M. T., Sarao, R. C., Wu, P.-C., Maslen, C. L., Glanville, R. W., Babcock, D., & Gregory, K. W. (2012). Mechanical property characterization of electrospun recombinant human tropoelastin for vascular graft biomaterials. *Acta Biomaterialia*, *8*(1), 225–233. https://doi.org/10.1016/j.actbio.2011.08.001

National Library of Medicine. (2023a, September 7). *CD80 CD80 molecule [ Homo sapiens (human) ]*. https://www.ncbi.nlm.nih.gov/gene?Db=gene&Cmd=DetailsSearch&Term=941

National Library of Medicine. (2023b, September 7). *CD86 CD86 molecule [ Homo sapiens (human) ]*. https://www.ncbi.nlm.nih.gov/gene/?term=NM_175862

National Library of Medicine. (2023c, September 7). *CD163 CD163 molecule [ Homo sapiens (human) ]*. https://www.ncbi.nlm.nih.gov/gene/9332

National Library of Medicine. (2023d, September 7). *CXCL10 C-X-C motif chemokine ligand 10 [ Homo sapiens (human) ]*. https://www.ncbi.nlm.nih.gov/gene/3627

National Library of Medicine. (2023e, September 7). *IL12B interleukin 12B [ Homo sapiens (human) ]*. https://www.ncbi.nlm.nih.gov/gene/3593

National Library of Medicine. (2023f, September 7). *MRC1 mannose receptor C-type 1 [ Homo sapiens (human) ]*. https://www.ncbi.nlm.nih.gov/gene/4360

National Library of Medicine. (2023g, September 7). *MT-CO2 mitochondrially encoded cytochrome c oxidase II [ Homo sapiens (human) ]*. https://www.ncbi.nlm.nih.gov/gene/4513

National Library of Medicine. (2023h, September 7). *VCAM1 vascular cell adhesion molecule 1 [ Homo sapiens (human) ]*. https://www.ncbi.nlm.nih.gov/gene/7412

National Library of Medicine. (2023i, September 11). *CD44 CD44 molecule (Indian blood group) [ Homo sapiens (human) ]*. https://www.ncbi.nlm.nih.gov/gene/960

National Library of Medicine. (2023j, September 11). *NOS2 nitric oxide synthase 2 [ Homo sapiens (human) ]*. https://www.ncbi.nlm.nih.gov/gene/4843

National Library of Medicine. (2023k, September 24). *NOS3 nitric oxide synthase 3 [ Homo sapiens (human) ]*. https://www.ncbi.nlm.nih.gov/gene/4846

National Library of Medicine. (2023l, September 24). *VEGFA vascular endothelial growth factor A [ Homo sapiens (human) ]*. https://www.ncbi.nlm.nih.gov/gene/7422

National Library of Medicine. (2023m, September 24). *VWF von Willebrand factor [ Homo sapiens (human) ]*. https://www.ncbi.nlm.nih.gov/gene/7450

National Library of Medicine. (2023n, September 27). *IL1B interleukin 1 beta [ Homo sapiens (human) ]*. https://www.ncbi.nlm.nih.gov/gene?Db=gene&Cmd=DetailsSearch&Term=3553

National Library of Medicine. (2023o, October 2). *ARG1 arginase 1 [ Homo sapiens (human) ]*. https://www.ncbi.nlm.nih.gov/gene/383

National Library of Medicine. (2023p, October 2). *EDN1 endothelin 1 [ Homo sapiens (human) ]*. https://www.ncbi.nlm.nih.gov/gene/?term=1906

National Library of Medicine. (2023q, October 2). *EGFR epidermal growth factor receptor [ Homo sapiens (human) ]*. https://www.ncbi.nlm.nih.gov/gene/1956

National Library of Medicine. (2023r, October 2). *IL1RN interleukin 1 receptor antagonist [ Homo sapiens (human) ]*. https://www.ncbi.nlm.nih.gov/gene/3557

National Library of Medicine. (2023s, October 2). *IL6 interleukin 6 [ Homo sapiens (human) ]*. https://www.ncbi.nlm.nih.gov/gene/3569

National Library of Medicine. (2023t, October 2). *KDR kinase insert domain receptor [ Homo sapiens (human) ]*. https://www.ncbi.nlm.nih.gov/gene/3791

National Library of Medicine. (2023u, October 2). *TNF tumor necrosis factor [ Homo sapiens (human) ]*. https://www.ncbi.nlm.nih.gov/gene/7124

OriGene Technologies Inc. (2023a). *COX2 (PTGS2) Human qPCR Primer Pair (NM_000963)*. https://www.origene.com/catalog/gene-expression/qpcr-primer-pairs/hp200900/cox2-ptgs2-human-qpcr-primer-pair-nm_000963

OriGene Technologies Inc. (2023b). *Endothelin 1 (EDN1) Human qPCR Primer Pair (NM_001955)*. https://www.origene.com/catalog/gene-expression/qpcr-primer-pairs/hp205717/endothelin-1-edn1-human-qpcr-primer-pair-nm_001955

OriGene Technologies Inc. (2023c). *VCAM1 Human qPCR Primer Pair (NM_080682)*. https://www.origene.com/catalog/gene-expression/qpcr-primer-pairs/hp230503/vcam1-human-qpcr-primer-pair-nm_080682

OriGene Technologies Inc. (2023d). *VEGF Receptor 1 (FLT1) Human qPCR Primer Pair (NM_002019)*. https://www.origene.com/catalog/gene-expression/qpcr-primer-pairs/hp205774/vegf-receptor-1-flt1-human-qpcr-primer-pair-nm_002019

OriGene Technologies Inc. (2023e). *VEGFA Human qPCR Primer Pair (NM_001025366)*. https://www.origene.com/catalog/gene-expression/qpcr-primer-pairs/hp202779/vegfa-human-qpcr-primer-pair-nm_001025366

Rodríguez-Soto, M. A., Suárez Vargas, N., Ayala-Velásquez, M., Aragón-Rivera, A. M., Ostos, C., Cruz, J. C., Muñoz Camargo, C., Kim, S., D’Amore, A., Wagner, W. R., & Briceño, J. C. (2023). Polyester urethane urea (PEUU) functionalization for enhanced anti-thrombotic performance: advancing regenerative cardiovascular devices through innovative surface modifications. *Frontiers in Bioengineering and Biotechnology*, *11*, 1–20. https://doi.org/10.3389/fbioe.2023.1257778

Saeki, H., & Tamaki, K. (2006). Thymus and activation regulated chemokine (TARC)/CCL17 and skin diseases. *Journal of Dermatological Science*, *43*(2), 75–84. https://doi.org/10.1016/j.jdermsci.2006.06.002

Shihan, M. H., Novo, S. G., Le Marchand, S. J., Wang, Y., & Duncan, M. K. (2021). A simple method for quantitating confocal fluorescent images. *Biochemistry and Biophysics Reports*, *25*, 100916. https://doi.org/10.1016/j.bbrep.2021.100916

Sun, Y., Ip, P., & Chakrabartty, A. (2017). Simple Elimination of Background Fluorescence in Formalin-Fixed Human Brain Tissue for Immunofluorescence Microscopy. *Journal of Visualized Experiments*, *127*. https://doi.org/10.3791/56188

Tang, C., Chen, S., Qian, H., & Huang, W. (2012). Interleukin‐23: as a drug target for autoimmune inflammatory diseases. *Immunology*, *135*(2), 112–124. https://doi.org/10.1111/j.1365-2567.2011.03522.x
